# Supplementary material for: Does size matter? An analysis of the niche width and vulnerability to climate change of fourteen species of the genus Crotalus from North America
Source: PeerJ. 2022 Apr 5;10:e13154. doi: 10.7717/peerj.13154 (PMC8992643; doi:10.7717/peerj.13154)
Supplement: Supplemental Information 1 — Data show the latitude and longitude for each species. [file peerj-10-13154-s001.docx]

Table S1. Geographic records of the 14 species of *Crotalus* analyzed in this study. Data show the latitude and longitude for each species.

| Species | Latitude (N) | Longitude (W) |
| --- | --- | --- |
| *C. atrox* | 24.215 | -98.481 |
| *C. atrox* | 23.86 | -99.14 |
| *C. atrox* | 32.196255 | -106.7528 |
| *C. atrox* | 23.91 | -99.09 |
| *C. atrox* | 34.031204 | -113.0826 |
| *C. atrox* | 34.491463 | -113.3855 |
| *C. atrox* | 33.83334 | -111.9507 |
| *C. atrox* | 33.827927 | -111.9512 |
| *C. atrox* | 34.469864 | -113.3142 |
| *C. atrox* | 31.539494 | -110.7562 |
| *C. atrox* | 29.33373 | -103.363 |
| *C. atrox* | 32.449986 | -106.0595 |
| *C. atrox* | 33.03738 | -115.6553 |
| *C. atrox* | 35.33194 | -104.0572 |
| *C. atrox* | 32.872776 | -114.4778 |
| *C. atrox* | 32.834167 | -114.4911 |
| *C. atrox* | 32.8275 | -114.6661 |
| *C. atrox* | 32.76428 | -114.8367 |
| *C. atrox* | 32.725346 | -114.6244 |
| *C. atrox* | 33.68028 | -116.1731 |
| *C. atrox* | 28.983334 | -112.3333 |
| *C. atrox* | 34.20783 | -113.0651 |
| *C. atrox* | 34.418354 | -112.9171 |
| *C. atrox* | 33.720554 | -116.2147 |
| *C. atrox* | 34.16313 | -112.8444 |
| *C. atrox* | 34.311115 | -112.9201 |
| *C. atrox* | 33.951534 | -112.7296 |
| *C. atrox* | 33.718613 | -116.3075 |
| *C. atrox* | 33.53429 | -111.4272 |
| *C. atrox* | 22.64 | -102.49 |
| *C. atrox* | 24.73 | -101.2 |
| *C. atrox* | 31.79 | -112.99 |
| *C. atrox* | 34.91341 | -101.5727 |
| *C. atrox* | 33.95754 | -100.8194 |
| *C. atrox* | 33.98543 | -100.4651 |
| *C. atrox* | 31.87 | -112.81 |
| *C. atrox* | 31.03 | -110.89 |
| *C. atrox* | 30.34 | -112.05 |
| *C. atrox* | 30.35 | -112.06 |
| *C. atrox* | 29.91 | -112.68 |
| *C. atrox* | 27.06 | -109.44 |
| *C. atrox* | 35.13976 | -114.5743 |
| *C. atrox* | 26.55 | -108.45 |
| *C. atrox* | 33.023056 | -114.6103 |
| *C. atrox* | 30.61368 | -103.3537 |
| *C. atrox* | 30.61132 | -103.3659 |
| *C. atrox* | 30.55756 | -103.4368 |
| *C. atrox* | 32.25002 | -101.4624 |
| *C. atrox* | 20.708 | -98.835 |
| *C. atrox* | 20.831 | -98.671 |
| *C. atrox* | 31.54 | -109.08 |
| *C. atrox* | 31.300135 | -103.6585 |
| *C. atrox* | 35.866543 | -98.45015 |
| *C. atrox* | 34.0039 | -117.062 |
| *C. atrox* | 31.943165 | -108.8786 |
| *C. atrox* | 31.939898 | -109.137 |
| *C. atrox* | 36.133396 | -95.92297 |
| *C. atrox* | 24.907 | -97.712 |
| *C. atrox* | 24.013 | -98.482 |
| *C. atrox* | 22.681 | -99.968 |
| *C. atrox* | 23.012 | -100.572 |
| *C. atrox* | 22.888 | -100.123 |
| *C. atrox* | 22.72 | -99.971 |
| *C. atrox* | 26.736 | -103.836 |
| *C. atrox* | 22.755 | -102.109 |
| *C. atrox* | 32.799103 | -105.9451 |
| *C. atrox* | 26.5388 | -98.3066 |
| *C. atrox* | 26.4966 | -98.3507 |
| *C. atrox* | 26.85 | -97.9166 |
| *C. atrox* | 31.893532 | -109.0076 |
| *C. atrox* | 29.322487 | -103.2591 |
| *C. atrox* | 29.976004 | -98.42448 |
| *C. atrox* | 32.119522 | -102.7073 |
| *C. atrox* | 28.653559 | -96.07877 |
| *C. atrox* | 32.024055 | -110.2973 |
| *C. atrox* | 30.336334 | -97.89439 |
| *C. atrox* | 31.86 | -113.12 |
| *C. atrox* | 18.819 | -96.319 |
| *C. atrox* | 33.84479 | -111.8864 |
| *C. atrox* | 33.952225 | -112.7086 |
| *C. atrox* | 33.71634 | -115.8084 |
| *C. atrox* | 33.69723 | -115.8014 |
| *C. atrox* | 33.93802 | -115.417 |
| *C. atrox* | 33.94088 | -115.417 |
| *C. atrox* | 33.79997 | -115.4475 |
| *C. atrox* | 33.78882 | -115.4009 |
| *C. atrox* | 33.66243 | -114.5718 |
| *C. atrox* | 33.7638 | -116.194 |
| *C. atrox* | 33.8246 | -116.3201 |
| *C. atrox* | 33.95818 | -116.6465 |
| *C. atrox* | 34.400593 | -114.2779 |
| *C. atrox* | 34.943123 | -114.6291 |
| *C. atrox* | 32.08999 | -106.9775 |
| *C. atrox* | 32.37944 | -104.4728 |
| *C. atrox* | 32.00994 | -104.4955 |
| *C. atrox* | 32.003914 | -104.5176 |
| *C. atrox* | 35.807446 | -103.8386 |
| *C. atrox* | 31.732641 | -108.8067 |
| *C. atrox* | 31.909029 | -107.669 |
| *C. atrox* | 29.32578 | -103.5746 |
| *C. atrox* | 29.8528 | -103.5894 |
| *C. atrox* | 29.57132 | -102.9458 |
| *C. atrox* | 29.27255 | -103.7546 |
| *C. atrox* | 29.29796 | -103.6813 |
| *C. atrox* | 29.26823 | -103.7611 |
| *C. atrox* | 31.38132 | -104.8365 |
| *C. atrox* | 30.48823 | -103.7463 |
| *C. atrox* | 29.28765 | -103.9106 |
| *C. atrox* | 30.92008 | -103.7914 |
| *C. atrox* | 31.72 | -113.1 |
| *C. atrox* | 30.457027 | -111.0898 |
| *C. atrox* | 29.96 | -111.11 |
| *C. atrox* | 30.61 | -111.05 |
| *C. atrox* | 31.14 | -112.44 |
| *C. atrox* | 31.96 | -113.18 |
| *C. atrox* | 30.96 | -112.35 |
| *C. atrox* | 29.02 | -110.96 |
| *C. atrox* | 28.79 | -110.96 |
| *C. atrox* | 28.65 | -111.01 |
| *C. atrox* | 28.61 | -111.02 |
| *C. atrox* | 27.06 | -109.54 |
| *C. atrox* | 27.23 | -109.47 |
| *C. atrox* | 28.06 | -110.96 |
| *C. atrox* | 28.24 | -111.02 |
| *C. atrox* | 28.33 | -111.04 |
| *C. atrox* | 28.44 | -111.05 |
| *C. atrox* | 27.19 | -109.55 |
| *C. atrox* | 27.16 | -109.52 |
| *C. atrox* | 27.39 | -109.42 |
| *C. atrox* | 27.03 | -109.44 |
| *C. atrox* | 26.77 | -109.33 |
| *C. atrox* | 31.313215 | -110.1461 |
| *C. atrox* | 27.07 | -109.04 |
| *C. atrox* | 32.002148 | -106.4907 |
| *C. atrox* | 32.651905 | -105.6377 |
| *C. atrox* | 30.96324 | -104.1696 |
| *C. atrox* | 29.39422 | -104.1513 |
| *C. atrox* | 31.75136 | -105.017 |
| *C. atrox* | 31.75326 | -105.3196 |
| *C. atrox* | 33.28343 | -114.7749 |
| *C. atrox* | 32.08833 | -108.8786 |
| *C. atrox* | 33.72606 | -111.8521 |
| *C. atrox* | 34.86306 | -112.0778 |
| *C. atrox* | 29.88759 | -98.65521 |
| *C. atrox* | 32.24055 | -106.8337 |
| *C. atrox* | 32.25507 | -106.8343 |
| *C. atrox* | 32.255066 | -106.8342 |
| *C. atrox* | 32.31222 | -106.7778 |
| *C. atrox* | 32.435577 | -106.7778 |
| *C. atrox* | 32.414444 | -106.829 |
| *C. atrox* | 32.283314 | -106.7528 |
| *C. atrox* | 32.31221 | -106.8293 |
| *C. atrox* | 31.942627 | -108.8598 |
| *C. atrox* | 31.71 | -113.12 |
| *C. atrox* | 30.02335 | -103.5721 |
| *C. atrox* | 29.16743 | -103.6097 |
| *C. atrox* | 33.64034 | -116.1395 |
| *C. atrox* | 33.66336 | -116.31 |
| *C. atrox* | 33.71864 | -116.3084 |
| *C. atrox* | 33.61033 | -114.5964 |
| *C. atrox* | 33.92628 | -114.5389 |
| *C. atrox* | 33.77978 | -116.4653 |
| *C. atrox* | 33.853046 | -113.2644 |
| *C. atrox* | 33.61174 | -110.6187 |
| *C. atrox* | 35.87583 | -106.1419 |
| *C. atrox* | 34.4237 | -105.3964 |
| *C. atrox* | 33.939354 | -106.8908 |
| *C. atrox* | 33.69374 | -106.6068 |
| *C. atrox* | 33.74514 | -106.545 |
| *C. atrox* | 33.646393 | -106.3714 |
| *C. atrox* | 33.863003 | -106.1877 |
| *C. atrox* | 33.542187 | -106.4402 |
| *C. atrox* | 35.48017 | -104.6154 |
| *C. atrox* | 27.15 | -109.49 |
| *C. atrox* | 33.84782 | -116.3107 |
| *C. atrox* | 32.34345 | -104.0665 |
| *C. atrox* | 32.07715 | -106.7757 |
| *C. atrox* | 31.1473 | -102.1827 |
| *C. atrox* | 33.46588 | -116.0981 |
| *C. atrox* | 33.68032 | -116.1739 |
| *C. atrox* | 33.01678 | -114.6079 |
| *C. atrox* | 33.01078 | -115.0913 |
| *C. atrox* | 33.2812 | -114.781 |
| *C. atrox* | 25.74 | -100.56 |
| *C. atrox* | 28.578775 | -109.5396 |
| *C. atrox* | 30.57 | -111.2 |
| *C. atrox* | 28.633364 | -110.1745 |
| *C. atrox* | 32.65163 | -96.90798 |
| *C. atrox* | 32.861706 | -107.5354 |
| *C. atrox* | 31.43 | -113.47 |
| *C. atrox* | 29.41 | -110.41 |
| *C. atrox* | 30.47 | -111.09 |
| *C. atrox* | 28.7 | -110.99 |
| *C. atrox* | 25.809 | -100.593 |
| *C. atrox* | 23.580364 | -99.75021 |
| *C. atrox* | 31.415 | -103.3338 |
| *C. atrox* | 31.415 | -103.5005 |
| *C. atrox* | 27.59 | -105.29 |
| *C. atrox* | 26.75 | -104.34 |
| *C. atrox* | 33.06272 | -107.298 |
| *C. atrox* | 32.96231 | -107.3121 |
| *C. atrox* | 32.50812 | -106.9569 |
| *C. atrox* | 29.309004 | -103.673 |
| *C. atrox* | 34.83069 | -95.64432 |
| *C. atrox* | 32.738846 | -104.8061 |
| *C. atrox* | 36.58222 | -98.87944 |
| *C. atrox* | 36.52421 | -98.87944 |
| *C. atrox* | 36.50971 | -98.87944 |
| *C. atrox* | 36.21194 | -96.26889 |
| *C. atrox* | 31.41694 | -108.9292 |
| *C. atrox* | 26.5 | -100.428 |
| *C. atrox* | 25.123 | -97.834 |
| *C. atrox* | 24.172 | -98.481 |
| *C. atrox* | 24.193 | -98.481 |
| *C. atrox* | 24.547 | -99.082 |
| *C. atrox* | 35.4 | -103.9 |
| *C. atrox* | 35.21 | -106.51 |
| *C. atrox* | 35.4 | -104.2 |
| *C. atrox* | 33.7 | -105.7 |
| *C. atrox* | 33.34 | -104.33 |
| *C. atrox* | 31.35 | -108.32 |
| *C. atrox* | 34.8 | -106.5 |
| *C. atrox* | 33.27 | -104.33 |
| *C. atrox* | 32.55 | -103.85 |
| *C. atrox* | 32.5 | -104.3 |
| *C. atrox* | 35.12 | -106.74 |
| *C. atrox* | 35.1 | -106.7 |
| *C. atrox* | 33.15 | -107.19 |
| *C. atrox* | 33.2 | -107.2 |
| *C. atrox* | 35.3 | -106.4 |
| *C. atrox* | 35.08 | -104.82 |
| *C. atrox* | 34.26 | -106.89 |
| *C. atrox* | 34.96 | -104.77 |
| *C. atrox* | 34.25 | -106.89 |
| *C. atrox* | 34.3 | -106.9 |
| *C. atrox* | 35.36 | -104.19 |
| *C. atrox* | 35.34 | -103.44 |
| *C. atrox* | 35.83 | -106.16 |
| *C. atrox* | 34.89 | -107.14 |
| *C. atrox* | 33.31 | -104.33 |
| *C. atrox* | 32.48 | -104.24 |
| *C. atrox* | 33.5 | -105.3 |
| *C. atrox* | 34.64 | -107.48 |
| *C. atrox* | 32.35 | -107.63 |
| *C. atrox* | 32.48 | -104.23 |
| *C. atrox* | 34.98 | -105.08 |
| *C. atrox* | 32.4 | -104.3 |
| *C. atrox* | 34.6 | -104.4 |
| *C. atrox* | 34.02 | -106.92 |
| *C. atrox* | 32.47 | -107.65 |
| *C. atrox* | 35.14 | -106.56 |
| *C. atrox* | 34.19 | -107.25 |
| *C. atrox* | 32.09 | -107.62 |
| *C. atrox* | 35.42 | -104.17 |
| *C. atrox* | 35.41 | -104.18 |
| *C. atrox* | 34.3 | -107.2 |
| *C. atrox* | 35.26 | -106.32 |
| *C. atrox* | 33.54 | -107.34 |
| *C. atrox* | 34.26 | -106.88 |
| *C. atrox* | 34.3 | -106.1 |
| *C. atrox* | 35.27 | -106.32 |
| *C. atrox* | 32.89 | -107.56 |
| *C. atrox* | 32.97 | -107.31 |
| *C. atrox* | 34.3 | -107.1 |
| *C. atrox* | 34.33 | -107.09 |
| *C. atrox* | 34.46 | -107.16 |
| *C. atrox* | 33.81 | -106.89 |
| *C. atrox* | 33.8 | -106.9 |
| *C. atrox* | 33.37 | -106.98 |
| *C. atrox* | 32.43 | -104.18 |
| *C. atrox* | 35.77 | -106.22 |
| *C. atrox* | 35.03 | -104.69 |
| *C. atrox* | 34.34 | -107.14 |
| *C. atrox* | 34.43 | -106.92 |
| *C. atrox* | 34.33 | -107.07 |
| *C. atrox* | 34.35 | -107.14 |
| *C. atrox* | 35.2 | -106.4 |
| *C. atrox* | 31.46 | -108.28 |
| *C. atrox* | 35.2 | -106.5 |
| *C. atrox* | 33.3 | -107.3 |
| *C. atrox* | 35.1 | -106.8 |
| *C. atrox* | 31.42 | -108.79 |
| *C. atrox* | 33.4 | -107.6 |
| *C. atrox* | 34.46 | -106.39 |
| *C. atrox* | 31.8 | -108.8 |
| *C. atrox* | 33.76 | -106.92 |
| *C. atrox* | 31.95 | -108.76 |
| *C. atrox* | 32.44 | -104.54 |
| *C. atrox* | 31.91 | -108.81 |
| *C. atrox* | 31.94 | -108.95 |
| *C. atrox* | 31.92 | -109.02 |
| *C. atrox* | 31.82 | -108.06 |
| *C. atrox* | 34.92 | -104.18 |
| *C. atrox* | 32 | -109 |
| *C. atrox* | 35.64 | -106.33 |
| *C. atrox* | 32.66 | -107.18 |
| *C. atrox* | 32 | -105 |
| *C. atrox* | 32.2 | -105.2 |
| *C. atrox* | 31.5 | -108.2 |
| *C. atrox* | 31.81 | -107.82 |
| *C. atrox* | 33.23 | -104.33 |
| *C. atrox* | 32.73 | -108.69 |
| *C. atrox* | 34.81 | -106.77 |
| *C. atrox* | 34.81 | -106.82 |
| *C. atrox* | 33.34 | -106.08 |
| *C. atrox* | 33.33 | -106.08 |
| *C. atrox* | 32.3 | -106.7 |
| *C. atrox* | 32.29 | -106.64 |
| *C. atrox* | 32.29 | -106.72 |
| *C. atrox* | 34.38 | -106.82 |
| *C. atrox* | 33.75 | -106.95 |
| *C. atrox* | 33.35 | -104.03 |
| *C. atrox* | 34.12 | -105.29 |
| *C. atrox* | 32.71 | -108.72 |
| *C. atrox* | 33.73 | -106.99 |
| *C. atrox* | 33.4 | -104.4 |
| *C. atrox* | 33.1 | -103.8 |
| *C. atrox* | 33.94 | -106.87 |
| *C. atrox* | 34.01 | -104.27 |
| *C. atrox* | 32.64 | -108.89 |
| *C. atrox* | 33.34 | -104.03 |
| *C. atrox* | 32.6 | -104 |
| *C. atrox* | 32.49 | -104.25 |
| *C. atrox* | 33.23 | -107.52 |
| *C. atrox* | 31.81 | -107.03 |
| *C. atrox* | 31.87 | -108.17 |
| *C. atrox* | 33.88 | -106.24 |
| *C. atrox* | 34.06 | -107.06 |
| *C. atrox* | 35.45 | -106.67 |
| *C. atrox* | 31.69 | -108.84 |
| *C. atrox* | 31.85 | -107.94 |
| *C. atrox* | 31.54 | -108.52 |
| *C. atrox* | 32.26 | -104.73 |
| *C. atrox* | 35.63 | -106.72 |
| *C. atrox* | 33.74 | -106.96 |
| *C. atrox* | 31.42 | -108.41 |
| *C. atrox* | 34.01 | -106.93 |
| *C. atrox* | 33.12 | -104.33 |
| *C. atrox* | 32.78 | -106.47 |
| *C. atrox* | 32.09 | -104.56 |
| *C. atrox* | 35.57 | -106.72 |
| *C. atrox* | 35.69 | -105.97 |
| *C. atrox* | 35.56 | -106.86 |
| *C. atrox* | 32.61 | -107.88 |
| *C. atrox* | 31.67 | -108.97 |
| *C. atrox* | 32.93 | -107.54 |
| *C. atrox* | 32.96 | -107.33 |
| *C. atrox* | 33.88 | -106.34 |
| *C. atrox* | 34.95 | -104.67 |
| *C. atrox* | 32.96 | -107.46 |
| *C. atrox* | 32.48 | -107.53 |
| *C. atrox* | 33.44 | -103.82 |
| *C. atrox* | 32.58 | -107.93 |
| *C. atrox* | 33.28 | -107.19 |
| *C. atrox* | 33.29 | -107.24 |
| *C. atrox* | 32.28 | -104.25 |
| *C. atrox* | 35.38 | -103.06 |
| *C. atrox* | 33.05 | -107.41 |
| *C. atrox* | 33.2 | -103.8 |
| *C. atrox* | 32.4 | -104.6 |
| *C. atrox* | 32.23 | -104.31 |
| *C. atrox* | 31.38 | -108.67 |
| *C. atrox* | 31.82 | -107.84 |
| *C. atrox* | 31.7 | -108.5 |
| *C. atrox* | 31.8 | -108.4 |
| *C. atrox* | 33.94 | -106.88 |
| *C. atrox* | 31.4 | -108.7 |
| *C. atrox* | 35.3 | -104.3 |
| *C. atrox* | 35.02 | -104.68 |
| *C. atrox* | 31.95 | -108.79 |
| *C. atrox* | 34.23 | -106.98 |
| *C. atrox* | 35.39 | -104.18 |
| *C. atrox* | 33.02 | -103.83 |
| *C. atrox* | 36.07 | -104.33 |
| *C. atrox* | 33.95 | -106.97 |
| *C. atrox* | 34.2 | -106.8 |
| *C. atrox* | 34.68 | -106.47 |
| *C. atrox* | 33.59 | -107.17 |
| *C. atrox* | 34.15 | -106.99 |
| *C. atrox* | 34.12 | -107.15 |
| *C. atrox* | 31.63 | -108.47 |
| *C. atrox* | 34.43 | -106.58 |
| *C. atrox* | 31.57 | -102.87 |
| *C. atrox* | 33.35 | -106.59 |
| *C. atrox* | 33.86 | -106.85 |
| *C. atrox* | 34.39 | -106.99 |
| *C. atrox* | 35.6 | -106.8 |
| *C. atrox* | 33.14 | -107.17 |
| *C. atrox* | 33.13 | -107.25 |
| *C. atrox* | 33.13 | -107.11 |
| *C. atrox* | 35.39 | -106.55 |
| *C. atrox* | 35.46 | -106.93 |
| *C. atrox* | 35.13 | -106.74 |
| *C. atrox* | 35.52 | -106.05 |
| *C. atrox* | 34.67 | -106.53 |
| *C. atrox* | 34.53 | -107.17 |
| *C. atrox* | 35.1 | -106.4 |
| *C. atrox* | 35.1 | -106.5 |
| *C. atrox* | 34.96 | -107.18 |
| *C. atrox* | 34.35 | -106.88 |
| *C. atrox* | 32.33 | -104.32 |
| *C. atrox* | 35.76 | -106.03 |
| *C. atrox* | 31.85 | -107.75 |
| *C. atrox* | 35.4 | -103.91 |
| *C. atrox* | 35.32 | -104.02 |
| *C. atrox* | 34.66 | -106.78 |
| *C. atrox* | 35.4 | -104.19 |
| *C. atrox* | 35.32 | -104.05 |
| *C. atrox* | 33.7 | -105.74 |
| *C. atrox* | 33.24 | -104.34 |
| *C. atrox* | 34.75 | -106.5 |
| *C. atrox* | 33.09 | -105.27 |
| *C. atrox* | 33.33 | -104.34 |
| *C. atrox* | 34.41 | -107.38 |
| *C. atrox* | 33.34 | -104.34 |
| *C. atrox* | 33.39 | -104.34 |
| *C. atrox* | 32.5 | -104.27 |
| *C. atrox* | 35.08 | -106.65 |
| *C. atrox* | 35.13 | -106.7 |
| *C. atrox* | 32.69 | -108.74 |
| *C. atrox* | 33.2 | -107.21 |
| *C. atrox* | 35.13 | -106.78 |
| *C. atrox* | 34.8 | -106.78 |
| *C. atrox* | 32.04 | -108.94 |
| *C. atrox* | 35.31 | -106.4 |
| *C. atrox* | 34.26 | -106.9 |
| *C. atrox* | 33.42 | -104.09 |
| *C. atrox* | 35.11 | -103.87 |
| *C. atrox* | 32.68 | -108.75 |
| *C. atrox* | 33.57 | -105.32 |
| *C. atrox* | 33.5 | -105.31 |
| *C. atrox* | 32.42 | -104.3 |
| *C. atrox* | 34.6 | -104.38 |
| *C. atrox* | 32.63 | -103.87 |
| *C. atrox* | 31.94 | -108.94 |
| *C. atrox* | 31.94 | -108.91 |
| *C. atrox* | 34.51 | -107.04 |
| *C. atrox* | 34.32 | -106.1 |
| *C. atrox* | 34.33 | -107.1 |
| *C. atrox* | 34.82 | -106.8 |
| *C. atrox* | 33.79 | -106.9 |
| *C. atrox* | 35.83 | -106.17 |
| *C. atrox* | 34.31 | -106.8 |
| *C. atrox* | 32.46 | -104.57 |
| *C. atrox* | 35.2 | -106.43 |
| *C. atrox* | 33.59 | -105.19 |
| *C. atrox* | 31.68 | -108.85 |
| *C. atrox* | 35.2 | -106.51 |
| *C. atrox* | 33.3 | -107.31 |
| *C. atrox* | 35.1 | -106.81 |
| *C. atrox* | 35.13 | -106.48 |
| *C. atrox* | 33.35 | -107.6 |
| *C. atrox* | 33.57 | -107.07 |
| *C. atrox* | 32.67 | -108.74 |
| *C. atrox* | 32.41 | -104.34 |
| *C. atrox* | 31.78 | -108.8 |
| *C. atrox* | 34.91 | -106.69 |
| *C. atrox* | 31.93 | -109 |
| *C. atrox* | 35.77 | -106.19 |
| *C. atrox* | 31.82 | -108.8 |
| *C. atrox* | 31.95 | -108.82 |
| *C. atrox* | 32.52 | -106.97 |
| *C. atrox* | 31.57 | -108.78 |
| *C. atrox* | 31.87 | -109.04 |
| *C. atrox* | 32 | -108.53 |
| *C. atrox* | 31.81 | -107.83 |
| *C. atrox* | 32 | -105.11 |
| *C. atrox* | 32.21 | -105.2 |
| *C. atrox* | 31.98 | -109.04 |
| *C. atrox* | 31.5 | -108.24 |
| *C. atrox* | 34.89 | -104.64 |
| *C. atrox* | 32.3 | -106.67 |
| *C. atrox* | 33.38 | -104.35 |
| *C. atrox* | 32.74 | -106.55 |
| *C. atrox* | 32.81 | -106.49 |
| *C. atrox* | 33.39 | -104.4 |
| *C. atrox* | 33.11 | -103.8 |
| *C. atrox* | 32.3 | -106.71 |
| *C. atrox* | 32.52 | -104.35 |
| *C. atrox* | 32.6 | -103.97 |
| *C. atrox* | 34.57 | -106.79 |
| *C. atrox* | 31.36 | -108.65 |
| *C. atrox* | 35.33 | -106.15 |
| *C. atrox* | 31.35 | -109.05 |
| *C. atrox* | 34.81 | -106.8 |
| *C. atrox* | 32.11 | -108.54 |
| *C. atrox* | 31.59 | -108.78 |
| *C. atrox* | 34.45 | -106.83 |
| *C. atrox* | 33.03 | -107.51 |
| *C. atrox* | 33.68 | -107.06 |
| *C. atrox* | 35.99 | -104.35 |
| *C. atrox* | 32.18 | -104.43 |
| *C. atrox* | 33.4 | -104.35 |
| *C. atrox* | 35.09 | -106.4 |
| *C. atrox* | 35.1 | -106.52 |
| *C. atrox* | 34.14 | -106.99 |
| *C. atrox* | 32.33 | -104.31 |
| *C. atrox* | 32.22 | -103.99 |
| *C. atrox* | 32.84703 | -115.6746 |
| *C. atrox* | 30.826155 | -112.6059 |
| *C. atrox* | 30.71556 | -112.5957 |
| *C. atrox* | 32.368332 | -115.8008 |
| *C. atrox* | 32.2517 | -110.7367 |
| *C. atrox* | 30.306 | -97.581 |
| *C. atrox* | 30.267 | -97.743 |
| *C. atrox* | 28.9004 | -100.148 |
| *C. atrox* | 27.506 | -99.507 |
| *C. atrox* | 32.03531 | -106.4649 |
| *C. atrox* | 29.80596 | -101.173 |
| *C. atrox* | 23.99389 | -103.0194 |
| *C. atrox* | 29.80807 | -103.1961 |
| *C. atrox* | 33.76366 | -106.0253 |
| *C. atrox* | 34.925 | -114.6422 |
| *C. atrox* | 31.281961 | -112.5336 |
| *C. atrox* | 33.919514 | -106.747 |
| *C. atrox* | 33.919613 | -106.74 |
| *C. atrox* | 34.294346 | -114.1553 |
| *C. atrox* | 32.7836 | -114.5371 |
| *C. atrox* | 32.8861 | -114.4577 |
| *C. atrox* | 33.990322 | -114.4729 |
| *C. atrox* | 32.2685 | -111.2175 |
| *C. atrox* | 31.8628 | -109.1408 |
| *C. atrox* | 31.5752 | -109.2566 |
| *C. atrox* | 35.04746 | -114.6591 |
| *C. atrox* | 35.255 | -107.985 |
| *C. atrox* | 32.2481 | -111.2005 |
| *C. atrox* | 32.2458 | -111.1774 |
| *C. atrox* | 31.9124 | -109.1418 |
| *C. atrox* | 31.6903 | -109.1317 |
| *C. atrox* | 31.6492 | -109.1797 |
| *C. atrox* | 31.5677 | -109.2616 |
| *C. atrox* | 32.0453 | -112.0207 |
| *C. atrox* | 31.5433 | -109.2839 |
| *C. atrox* | 31.9136 | -109.1068 |
| *C. atrox* | 31.94328 | -108.9077 |
| *C. atrox* | 31.6652 | -109.1496 |
| *C. atrox* | 31.8789 | -109.0621 |
| *C. atrox* | 31.6192 | -109.2607 |
| *C. atrox* | 31.5583 | -109.2641 |
| *C. atrox* | 31.937561 | -108.9583 |
| *C. atrox* | 31.6152 | -109.2153 |
| *C. atrox* | 31.45 | -109.4649 |
| *C. atrox* | 31.9136 | -109.1408 |
| *C. atrox* | 31.9136 | -109.1051 |
| *C. atrox* | 31.9178 | -109.156 |
| *C. atrox* | 31.7051 | -109.1221 |
| *C. atrox* | 31.5444 | -109.2829 |
| *C. atrox* | 31.945368 | -108.9222 |
| *C. atrox* | 32.2019 | -111.2174 |
| *C. atrox* | 31.9142 | -109.1236 |
| *C. atrox* | 32.37853 | -111.3387 |
| *C. atrox* | 33.973804 | -113.5726 |
| *C. atrox* | 32.08385 | -111.2949 |
| *C. atrox* | 31.98915 | -111.093 |
| *C. atrox* | 27.45694 | -105.4 |
| *C. atrox* | 31.9935 | -110.339 |
| *C. atrox* | 31.623667 | -111.4107 |
| *C. atrox* | 31.80092 | -110.8933 |
| *C. atrox* | 31.84184 | -110.9598 |
| *C. atrox* | 32.23756 | -111.0405 |
| *C. atrox* | 31.76704 | -110.8873 |
| *C. atrox* | 33.15333 | -107.2067 |
| *C. atrox* | 31.300554 | -113.5448 |
| *C. atrox* | 29.046183 | -111.1215 |
| *C. atrox* | 28.631573 | -111.3863 |
| *C. atrox* | 28.85028 | -111.2471 |
| *C. atrox* | 30.044 | -99.119 |
| *C. atrox* | 33.791264 | -116.6761 |
| *C. atrox* | 27.052982 | -99.73173 |
| *C. atrox* | 25.854034 | -99.03295 |
| *C. atrox* | 25.799723 | -98.27778 |
| *C. atrox* | 26.008055 | -98.48917 |
| *C. atrox* | 28.778294 | -112.2801 |
| *C. atrox* | 31.443 | -100.83 |
| *C. atrox* | 33.57192 | -116.0727 |
| *C. atrox* | 33.935493 | -116.54 |
| *C. atrox* | 33.60856 | -114.5926 |
| *C. atrox* | 31.4937 | -110.2568 |
| *C. atrox* | 30.6 | -108.0833 |
| *C. atrox* | 29.360853 | -110.9693 |
| *C. atrox* | 29.457218 | -110.9923 |
| *C. atrox* | 29.295 | -99.087 |
| *C. atrox* | 30.475822 | -106.5226 |
| *C. atrox* | 29.880184 | -106.8158 |
| *C. atrox* | 33.642883 | -116.3605 |
| *C. atrox* | 31.8726 | -109.0927 |
| *C. atrox* | 26.64002 | -97.789 |
| *C. atrox* | 31.9136 | -109.0898 |
| *C. atrox* | 31.735134 | -109.0318 |
| *C. atrox* | 29.550228 | -106.3316 |
| *C. atrox* | 29.82389 | -106.7359 |
| *C. atrox* | 32.0476 | -112.0228 |
| *C. atrox* | 31.9949 | -111.9806 |
| *C. atrox* | 32.0141 | -111.9959 |
| *C. atrox* | 31.9058 | -109.1188 |
| *C. atrox* | 31.8969 | -109.0937 |
| *C. atrox* | 26.859291 | -109.3786 |
| *C. atrox* | 31.960777 | -113.0879 |
| *C. atrox* | 31.9945 | -111.5458 |
| *C. atrox* | 32.2217 | -110.9258 |
| *C. atrox* | 31.9575 | -110.9805 |
| *C. atrox* | 32.6478 | -111.0247 |
| *C. atrox* | 30.149874 | -111.0973 |
| *C. atrox* | 35.0431 | -114.6217 |
| *C. atrox* | 35.0258 | -114.2925 |
| *C. atrox* | 33.223316 | -110.2259 |
| *C. atrox* | 32.9002 | -109.2674 |
| *C. atrox* | 30.72 | -112.66 |
| *C. atrox* | 30.77 | -112.84 |
| *C. atrox* | 23.92 | -103.14 |
| *C. atrox* | 31.09 | -112.42 |
| *C. atrox* | 29.77 | -111.06 |
| *C. atrox* | 29.04 | -110.9 |
| *C. atrox* | 27.34 | -105.47 |
| *C. atrox* | 31.31667 | -113.5369 |
| *C. atrox* | 29.1 | -111.21 |
| *C. atrox* | 27.14 | -109.9 |
| *C. atrox* | 28.85 | -111.02 |
| *C. atrox* | 27.266 | -100.112 |
| *C. atrox* | 27.05 | -99.73 |
| *C. atrox* | 25.794 | -99.18 |
| *C. atrox* | 25.83 | -99.01 |
| *C. atrox* | 25.77472 | -98.35333 |
| *C. atrox* | 26.005 | -98.49444 |
| *C. atrox* | 28.8 | -112.32 |
| *C. atrox* | 28.03944 | -105.3106 |
| *C. atrox* | 29.44 | -110.96 |
| *C. atrox* | 29.55 | -110.96 |
| *C. atrox* | 30.48 | -106.53 |
| *C. atrox* | 29.98 | -106.38 |
| *C. atrox* | 29.55 | -106.38 |
| *C. atrox* | 26.83 | -106.79 |
| *C. atrox* | 26.93 | -109.24 |
| *C. atrox* | 31.96 | -113.12 |
| *C. atrox* | 30.13 | -111.12 |
| *C. atrox* | 31.24 | -106.28 |
| *C. atrox* | 31.25 | -106.28 |
| *C. atrox* | 31.1875 | -106.5058 |
| *C. atrox* | 27.218 | -100.1 |
| *C. atrox* | 34.36665 | -95.92992 |
| *C. atrox* | 34.5509 | -95.96767 |
| *C. atrox* | 25.97 | -101.01 |
| *C. atrox* | 26.38 | -101.41 |
| *C. atrox* | 35.0495 | -95.30715 |
| *C. atrox* | 34.9915 | -95.30715 |
| *C. atrox* | 34.66583 | -98.48612 |
| *C. atrox* | 34.701767 | -98.67704 |
| *C. atrox* | 34.71677 | -98.70754 |
| *C. atrox* | 34.947998 | -95.30715 |
| *C. atrox* | 34.823864 | -98.80055 |
| *C. atrox* | 34.781403 | -94.65463 |
| *C. atrox* | 34.767803 | -94.62863 |
| *C. atrox* | 34.6279 | -95.20525 |
| *C. atrox* | 35.968037 | -98.35005 |
| *C. atrox* | 35.84904 | -98.41255 |
| *C. atrox* | 34.7524 | -95.04324 |
| *C. atrox* | 34.866962 | -98.86845 |
| *C. atrox* | 34.431488 | -97.2001 |
| *C. atrox* | 34.902752 | -101.613 |
| *C. atrox* | 34.9685 | -94.72113 |
| *C. atrox* | 36.02504 | -98.43085 |
| *C. atrox* | 36.035236 | -98.41815 |
| *C. atrox* | 34.16379 | -98.59264 |
| *C. atrox* | 36.05584 | -98.39285 |
| *C. atrox* | 34.900616 | -99.7432 |
| *C. atrox* | 34.45199 | -97.1228 |
| *C. atrox* | 36.583633 | -98.87727 |
| *C. atrox* | 34.941956 | -99.64597 |
| *C. atrox* | 29.212204 | -103.0189 |
| *C. atrox* | 30.892355 | -102.8534 |
| *C. atrox* | 34.92946 | -99.73387 |
| *C. atrox* | 34.900063 | -99.17366 |
| *C. atrox* | 34.934853 | -101.7109 |
| *C. atrox* | 25.900314 | -101.1503 |
| *C. atrox* | 34.876762 | -99.29706 |
| *C. atrox* | 35.971043 | -98.44135 |
| *C. atrox* | 34.886456 | -99.70898 |
| *C. atrox* | 26.4903 | -101.3503 |
| *C. atrox* | 34.44959 | -97.2098 |
| *C. atrox* | 34.67597 | -98.64704 |
| *C. atrox* | 34.711067 | -98.44774 |
| *C. atrox* | 34.681667 | -98.67674 |
| *C. atrox* | 34.677967 | -98.64234 |
| *C. atrox* | 34.65267 | -98.56714 |
| *C. atrox* | 32.932407 | -112.804 |
| *C. atrox* | 32.430378 | -110.7053 |
| *C. atrox* | 32.62203 | -110.9969 |
| *C. atrox* | 36.117336 | -98.31706 |
| *C. atrox* | 35.55149 | -94.84643 |
| *C. atrox* | 34.40691 | -95.91202 |
| *C. atrox* | 35.020954 | -99.83278 |
| *C. atrox* | 34.929657 | -99.56686 |
| *C. atrox* | 34.498 | -96.04707 |
| *C. atrox* | 34.54244 | -95.94116 |
| *C. atrox* | 34.52543 | -96.03044 |
| *C. atrox* | 31.99908 | -109.176 |
| *C. atrox* | 35.70019 | -94.82942 |
| *C. atrox* | 36.115864 | -98.31663 |
| *C. atrox* | 36.76973 | -99.11379 |
| *C. atrox* | 34.744568 | -98.53174 |
| *C. atrox* | 34.668804 | -94.60282 |
| *C. atrox* | 34.72297 | -98.72385 |
| *C. atrox* | 34.797665 | -98.77824 |
| *C. atrox* | 34.742466 | -98.66484 |
| *C. atrox* | 34.76197 | -98.58975 |
| *C. atrox* | 34.801266 | -98.77104 |
| *C. atrox* | 34.747963 | -98.76025 |
| *C. atrox* | 34.732563 | -98.71365 |
| *C. atrox* | 36.360435 | -98.34825 |
| *C. atrox* | 34.43649 | -97.1413 |
| *C. atrox* | 34.70987 | -98.62865 |
| *C. atrox* | 36.300236 | -98.53316 |
| *C. atrox* | 29.58 | -98.4 |
| *C. atrox* | 32.32611 | -106.5553 |
| *C. atrox* | 32.31526 | -106.5477 |
| *C. atrox* | 32.38083 | -106.4789 |
| *C. atrox* | 32.380745 | -106.4791 |
| *C. atrox* | 29.21199 | -103.0185 |
| *C. atrox* | 26.68 | -103.75 |
| *C. atrox* | 32.204666 | -112.5673 |
| *C. atrox* | 29.145 | -105.485 |
| *C. atrox* | 25.263327 | -104.0254 |
| *C. atrox* | 25.236 | -103.9197 |
| *C. atrox* | 24.69 | -101.77 |
| *C. atrox* | 24.66 | -101.85 |
| *C. atrox* | 24.7 | -101.77 |
| *C. atrox* | 32.79107 | -115.7651 |
| *C. atrox* | 33.71253 | -115.4022 |
| *C. atrox* | 28.636677 | -110.1998 |
| *C. atrox* | 29.84842 | -101.1545 |
| *C. atrox* | 30.47952 | -102.0921 |
| *C. atrox* | 30.47343 | -101.1281 |
| *C. atrox* | 29.875183 | -103.2523 |
| *C. atrox* | 32.224167 | -111.1586 |
| *C. atrox* | 31.810833 | -109.0492 |
| *C. atrox* | 32.7314 | -114.7492 |
| *C. atrox* | 32.79137 | -115.7685 |
| *C. atrox* | 33.52533 | -114.6561 |
| *C. atrox* | 32.58 | -116.0533 |
| *C. atrox* | 33.66217 | -112.4671 |
| *C. atrox* | 32.7884 | -115.5618 |
| *C. atrox* | 33.96802 | -112.7358 |
| *C. atrox* | 33.57172 | -116.0773 |
| *C. atrox* | 32.10917 | -104.4711 |
| *C. atrox* | 35.9961 | -111.986 |
| *C. atrox* | 32.72535 | -114.6244 |
| *C. atrox* | 34.31728 | -112.9224 |
| *C. atrox* | 32.38815 | -98.97763 |
| *C. atrox* | 31.71 | -113.31 |
| *C. atrox* | 32.1401 | -107.5965 |
| *C. atrox* | 32.17931 | -107.6449 |
| *C. atrox* | 32.16825 | -107.6334 |
| *C. atrox* | 33.126 | -115.513 |
| *C. atrox* | 33.38559 | -111.8772 |
| *C. atrox* | 34.41836 | -112.9171 |
| *C. atrox* | 33.98587 | -112.3596 |
| *C. atrox* | 34.39783 | -112.2363 |
| *C. atrox* | 32.72607 | -114.724 |
| *C. atrox* | 31.23 | -111.7 |
| *C. atrox* | 32.42056 | -104.2283 |
| *C. atrox* | 33.42676 | -111.8252 |
| *C. atrox* | 34.41752 | -112.7121 |
| *C. atrox* | 33.48669 | -111.8252 |
| *C. atrox* | 33.95153 | -112.7296 |
| *C. atrox* | 32.8328 | -114.5561 |
| *C. atrox* | 33.0211 | -114.6889 |
| *C. atrox* | 32.79313 | -115.6911 |
| *C. atrox* | 29.68333 | -110.95 |
| *C. atrox* | 34.2302 | -113.0984 |
| *C. atrox* | 33.04781 | -112.6658 |
| *C. atrox* | 32.9785 | -115.5289 |
| *C. atrox* | 32.84733 | -115.7572 |
| *C. atrox* | 35.99768 | -98.31645 |
| *C. atrox* | 31.61275 | -111.0395 |
| *C. atrox* | 27.94197 | -110.6439 |
| *C. atrox* | 32.9709 | -115.2594 |
| *C. atrox* | 34.77194 | -106.86 |
| *C. atrox* | 34.70641 | -112.3978 |
| *C. atrox* | 35.0827 | -106.7735 |
| *C. atrox* | 33.22825 | -115.6353 |
| *C. atrox* | 31.88367 | -106.4791 |
| *C. atrox* | 34.70167 | -98.67665 |
| *C. atrox* | 34.73254 | -98.71162 |
| *C. atrox* | 34.75059 | -98.74674 |
| *C. atrox* | 35.08444 | -106.6506 |
| *C. atrox* | 34.70681 | -98.70497 |
| *C. atrox* | 34.16253 | -112.8507 |
| *C. atrox* | 35.14714 | -107.8509 |
| *C. atrox* | 32.8111 | -115.379 |
| *C. atrox* | 27.95 | -111.05 |
| *C. atrox* | 31.96558 | -106.3494 |
| *C. atrox* | 32.82543 | -114.4969 |
| *C. atrox* | 32.74014 | -114.7059 |
| *C. atrox* | 33.03727 | -115.6207 |
| *C. atrox* | 30.91235 | -103.1321 |
| *C. atrox* | 33.64167 | -105.8767 |
| *C. atrox* | 33.55407 | -98.64001 |
| *C. atrox* | 33.58523 | -98.63758 |
| *C. atrox* | 33.55407 | -98.64056 |
| *C. atrox* | 33.62627 | -98.6621 |
| *C. atrox* | 35.83194 | -106.1561 |
| *C. atrox* | 28.99417 | -112.3861 |
| *C. atrox* | 32.69376 | -115.4236 |
| *C. atrox* | 32.70177 | -115.2676 |
| *C. atrox* | 29.92093 | -101.9196 |
| *C. atrox* | 33.63671 | -111.6746 |
| *C. atrox* | 32.46976 | -100.0113 |
| *C. atrox* | 27.1 | -109.14 |
| *C. atrox* | 29.18333 | -112.4 |
| *C. atrox* | 33.67623 | -116.1776 |
| *C. atrox* | 29.54613 | -104.3401 |
| *C. atrox* | 29.1 | -110.95 |
| *C. atrox* | 31.801 | -106.4215 |
| *C. atrox* | 33.27596 | -112.1841 |
| *C. atrox* | 27.03 | -102.11 |
| *C. atrox* | 33.42456 | -112.0029 |
| *C. atrox* | 25.61 | -101.4 |
| *C. atrox* | 22.65 | -98.25 |
| *C. atrox* | 32.53706 | -110.8977 |
| *C. atrox* | 35.14721 | -107.9041 |
| *C. atrox* | 33.44834 | -112.3856 |
| *C. atrox* | 23.818 | -99.12 |
| *C. atrox* | 27.262 | -99.636 |
| *C. atrox* | 24.488 | -98.331 |
| *C. atrox* | 24.371 | -98.39 |
| *C. atrox* | 24.018 | -98.836 |
| *C. atrox* | 24.466 | -98.888 |
| *C. atrox* | 23.83 | -99.15 |
| *C. atrox* | 26.4 | -109.22 |
| *C. atrox* | 28.43 | -110.9 |
| *C. atrox* | 27.21 | -99.64 |
| *C. atrox* | 27.06 | -99.72 |
| *C. atrox* | 27.17 | -99.68 |
| *C. atrox* | 24.03 | -98.85 |
| *C. atrox* | 28.23 | -111.29 |
| *C. atrox* | 31.12 | -110.92 |
| *C. atrox* | 20.887 | -99.917 |
| *C. atrox* | 20.89 | -99.93 |
| *C. atrox* | 26.96 | -102.09 |
| *C. atrox* | 27.04 | -102.07 |
| *C. atrox* | 24.5 | -98.89 |
| *C. atrox* | 24.65 | -99.07 |
| *C. atrox* | 24.78 | -98.58 |
| *C. atrox* | 22.00333 | -98.77889 |
| *C. atrox* | 24.15 | -99.15 |
| *C. atrox* | 20.444528 | -98.675 |
| *C. atrox* | 20.662306 | -98.77897 |
| *C. atrox* | 34.98028 | -101.9183 |
| *C. atrox* | 25.782 | -100.186 |
| *C. atrox* | 23.771 | -99.923 |
| *C. atrox* | 27.242 | -100.131 |
| *C. atrox* | 26 | -100.528 |
| *C. atrox* | 25.697 | -99.626 |
| *C. atrox* | 25.719 | -100.431 |
| *C. atrox* | 26.016 | -98.261 |
| *C. atrox* | 25.703 | -99.262 |
| *C. atrox* | 25.564 | -100.158 |
| *C. atrox* | 25.625 | -100.072 |
| *C. atrox* | 25.52 | -100.201 |
| *C. atrox* | 26.01 | -100.168 |
| *C. atrox* | 25.495 | -100.189 |
| *C. atrox* | 24.754 | -99.718 |
| *C. atrox* | 24.85 | -99.583 |
| *C. atrox* | 25.994 | -100.211 |
| *C. atrox* | 25.964 | -100.29 |
| *C. atrox* | 26.952 | -100.504 |
| *C. atrox* | 27.123 | -100.386 |
| *C. atrox* | 27.559 | -99.936 |
| *C. atrox* | 27.088 | -100.331 |
| *C. atrox* | 23.971 | -100.024 |
| *C. atrox* | 26.523 | -99.402 |
| *C. atrox* | 26.239 | -99.722 |
| *C. atrox* | 26.33 | -99.827 |
| *C. atrox* | 24.827 | -100.076 |
| *C. atrox* | 26.404 | -99.097 |
| *C. atrox* | 26.238 | -99.126 |
| *C. atrox* | 22.928 | -98.592 |
| *C. atrox* | 24.723 | -99.366 |
| *C. atrox* | 23.715 | -98.976 |
| *C. atrox* | 25.579 | -100.238 |
| *C. atrox* | 25.172 | -98.606 |
| *C. atrox* | 25.126 | -98.593 |
| *C. atrox* | 25.074 | -98.46 |
| *C. atrox* | 24.622 | -98.312 |
| *C. atrox* | 26.531 | -99.363 |
| *C. atrox* | 26.533 | -99.346 |
| *C. atrox* | 26.594 | -99.292 |
| *C. atrox* | 26.531 | -99.357 |
| *C. atrox* | 23.437 | -99.532 |
| *C. atrox* | 24.708 | -98.472 |
| *C. atrox* | 24.826 | -98.632 |
| *C. atrox* | 23.035 | -98.841 |
| *C. atrox* | 36.117283 | -98.3167 |
| *C. atrox* | 35.866486 | -98.44981 |
| *C. atrox* | 26.89 | -104.92 |
| *C. atrox* | 28.1 | -110.96 |
| *C. atrox* | 28.78 | -112.26 |
| *C. atrox* | 28.98 | -112.34 |
| *C. atrox* | 26.03 | -100.6 |
| *C. atrox* | 30.08 | -111.13 |
| *C. atrox* | 30.26 | -111.12 |
| *C. atrox* | 28.32 | -106.09 |
| *C. atrox* | 26.67 | -105.14 |
| *C. atrox* | 26.35 | -103.96 |
| *C. atrox* | 26.94 | -102.12 |
| *C. atrox* | 26.69 | -102.34 |
| *C. atrox* | 25.38 | -102.87 |
| *C. atrox* | 16.41 | -95.64 |
| *C. atrox* | 22.52 | -99.79 |
| *C. atrox* | 25.24 | -103.65 |
| *C. atrox* | 28.96 | -102.8 |
| *C. atrox* | 29.4 | -105.18 |
| *C. atrox* | 29.58 | -105.15 |
| *C. atrox* | 29 | -111.05 |
| *C. atrox* | 28.01 | -111.03 |
| *C. atrox* | 31.72 | -113.01 |
| *C. atrox* | 23.35 | -99.15 |
| *C. atrox* | 16.32444 | -95.23889 |
| *C. atrox* | 24.8 | -99.56 |
| *C. atrox* | 25.48 | -103.79 |
| *C. atrox* | 24.87 | -103.6958 |
| *C. atrox* | 22.29 | -99.01 |
| *C. atrox* | 22.44 | -99.65 |
| *C. atrox* | 24.15 | -98.57 |
| *C. atrox* | 24.16 | -98.54 |
| *C. atrox* | 30.04 | -111.12 |
| *C. atrox* | 29.26 | -110.96 |
| *C. atrox* | 27.24 | -100.2 |
| *C. atrox* | 27.32 | -109.69 |
| *C. atrox* | 31.42 | -106.48 |
| *C. atrox* | 30.73 | -107.93 |
| *C. atrox* | 27.46 | -110.29 |
| *C. atrox* | 25.04 | -98 |
| *C. atrox* | 16.18 | -95.24 |
| *C. atrox* | 26.7 | -104.27 |
| *C. atrox* | 25.87 | -107.03 |
| *C. atrox* | 25.96 | -107.12 |
| *C. atrox* | 25.98 | -107.16 |
| *C. atrox* | 26 | -107.21 |
| *C. atrox* | 25.84 | -106.98 |
| *C. atrox* | 25.88 | -107.05 |
| *C. atrox* | 25.85 | -107 |
| *C. atrox* | 25.98 | -107.14 |
| *C. atrox* | 27.77 | -101.12 |
| *C. atrox* | 27.75 | -101.15 |
| *C. atrox* | 27.72 | -101.16 |
| *C. atrox* | 27.68 | -101.17 |
| *C. atrox* | 27.63 | -101.18 |
| *C. atrox* | 26.98 | -101.87 |
| *C. atrox* | 28.54 | -105.43 |
| *C. atrox* | 28.8 | -107.98 |
| *C. atrox* | 16.34944 | -95.33528 |
| *C. atrox* | 25.76 | -102.81 |
| *C. atrox* | 27.5 | -108.81 |
| *C. atrox* | 27.02 | -109.64 |
| *C. atrox* | 28.17 | -109.93 |
| *C. atrox* | 27.08 | -109.34 |
| *C. atrox* | 26.28 | -108.99 |
| *C. atrox* | 27.11 | -109.44 |
| *C. atrox* | 24.86 | -99.44 |
| *C. atrox* | 25.17 | -99.56 |
| *C. atrox* | 24.852 | -98.154 |
| *C. atrox* | 24.85 | -98.14 |
| *C. atrox* | 26.45 | -109.04 |
| *C. atrox* | 16.32 | -95.26 |
| *C. atrox* | 23.23 | -100.64 |
| *C. atrox* | 23.84 | -99.159 |
| *C. atrox* | 23.81 | -99.15 |
| *C. atrox* | 23.69 | -100.58 |
| *C. atrox* | 23.64556 | -100.6436 |
| *C. atrox* | 30.5 | -109.47 |
| *C. atrox* | 26.949 | -99.992 |
| *C. atrox* | 26.89 | -99.81 |
| *C. atrox* | 16.32 | -95.47 |
| *C. atrox* | 16.2 | -95.24 |
| *C. atrox* | 16.27 | -95.24 |
| *C. atrox* | 16.31 | -95.47 |
| *C. atrox* | 35.05 | -114.6333 |
| *C. atrox* | 22.247 | -98.273 |
| *C. atrox* | 25.862 | -97.506 |
| *C. atrox* | 23.763 | -98.211 |
| *C. atrox* | 23.27 | -98.77 |
| *C. atrox* | 27.169 | -99.632 |
| *C. atrox* | 27.256 | -99.616 |
| *C. atrox* | 31.76357 | -104.9323 |
| *C. atrox* | 32.8267 | -114.5708 |
| *C. atrox* | 35.167 | -106.5 |
| *C. atrox* | 33.124428 | -115.5137 |
| *C. atrox* | 34.71667 | -98.70721 |
| *C. atrox* | 33.45701 | -114.9199 |
| *C. atrox* | 32.79674 | -114.6314 |
| *C. atrox* | 31.90722 | -109.1089 |
| *C. atrox* | 26.686 | -99.956 |
| *C. atrox* | 27.168 | -100.368 |
| *C. atrox* | 32.019505 | -106.499 |
| *C. atrox* | 28.31621 | -99.96173 |
| *C. atrox* | 31.87562 | -106.5517 |
| *C. atrox* | 32.97356 | -107.3081 |
| *C. atrox* | 30.52215 | -102.9275 |
| *C. atrox* | 30.25548 | -103.572 |
| *C. atrox* | 29.28479 | -103.8718 |
| *C. atrox* | 30.66754 | -100.2439 |
| *C. atrox* | 32.08745 | -106.7134 |
| *C. atrox* | 32.09398 | -108.3235 |
| *C. atrox* | 36.65466 | -98.96629 |
| *C. atrox* | 31.926071 | -107.0893 |
| *C. atrox* | 31.06164 | -105.4972 |
| *C. atrox* | 31.97477 | -110.7217 |
| *C. atrox* | 31.93928 | -106.5833 |
| *C. atrox* | 31.9522 | -106.5823 |
| *C. atrox* | 32.2482 | -111.2387 |
| *C. atrox* | 31.94485 | -106.5824 |
| *C. atrox* | 31.4176 | -108.3411 |
| *C. atrox* | 33.39869 | -104.349 |
| *C. atrox* | 31.94202 | -106.5823 |
| *C. atrox* | 32.001396 | -106.4437 |
| *C. atrox* | 32.615776 | -107.0294 |
| *C. atrox* | 31.82951 | -106.1016 |
| *C. atrox* | 31.9976 | -106.5833 |
| *C. atrox* | 32.08745 | -106.7139 |
| *C. atrox* | 31.93087 | -106.5823 |
| *C. atrox* | 31.94287 | -108.8593 |
| *C. atrox* | 31.93808 | -108.4074 |
| *C. atrox* | 31.89437 | -106.5824 |
| *C. atrox* | 32.24835 | -107.6442 |
| *C. atrox* | 32.01453 | -106.5587 |
| *C. atrox* | 31.97507 | -106.5833 |
| *C. atrox* | 32.24292 | -107.6212 |
| *C. atrox* | 31.93665 | -106.5833 |
| *C. atrox* | 31.90482 | -106.5487 |
| *C. atrox* | 32.01853 | -106.5008 |
| *C. atrox* | 31.90762 | -106.5706 |
| *C. atrox* | 32.11167 | -106.6619 |
| *C. atrox* | 31.843555 | -108.1194 |
| *C. atrox* | 30.73194 | -104.9847 |
| *C. atrox* | 31.19488 | -104.8518 |
| *C. atrox* | 32.22525 | -111.0374 |
| *C. atrox* | 31.926018 | -107.0893 |
| *C. atrox* | 31.943703 | -107.089 |
| *C. atrox* | 33.7434 | -106.9589 |
| *C. atrox* | 31.23354 | -99.69322 |
| *C. atrox* | 31.00507 | -105.5535 |
| *C. atrox* | 32.550697 | -106.9943 |
| *C. atrox* | 31.76813 | -105.4057 |
| *C. atrox* | 31.82557 | -106.1278 |
| *C. atrox* | 31.97129 | -106.9748 |
| *C. atrox* | 29.75813 | -98.50745 |
| *C. atrox* | 32.09154 | -105.0942 |
| *C. atrox* | 31.17931 | -105.3554 |
| *C. atrox* | 32.37111 | -106.5597 |
| *C. atrox* | 32.92925 | -107.5496 |
| *C. atrox* | 28.4962 | -100.1445 |
| *C. atrox* | 30.73 | -104.9883 |
| *C. atrox* | 33.57617 | -107.1883 |
| *C. atrox* | 32.234165 | -107.2505 |
| *C. atrox* | 31.84745 | -105.2009 |
| *C. atrox* | 28.75923 | -98.53999 |
| *C. atrox* | 28.54661 | -98.34244 |
| *C. atrox* | 31.86109 | -106.4301 |
| *C. atrox* | 31.97767 | -106.3379 |
| *C. atrox* | 30.09614 | -104.8173 |
| *C. atrox* | 31.68143 | -106.219 |
| *C. atrox* | 31.69402 | -106 |
| *C. atrox* | 31.86435 | -106.5485 |
| *C. atrox* | 31.48992 | -109.35 |
| *C. atrox* | 31.787672 | -107.2043 |
| *C. atrox* | 30.92844 | -102.6048 |
| *C. atrox* | 31.14781 | -104.8079 |
| *C. atrox* | 32.14806 | -108.9436 |
| *C. atrox* | 32.09504 | -105.5487 |
| *C. atrox* | 27.46376 | -98.95709 |
| *C. atrox* | 27.85013 | -99.72995 |
| *C. atrox* | 29.81659 | -101.5454 |
| *C. atrox* | 30.05152 | -102.1649 |
| *C. atrox* | 31.92206 | -105.9507 |
| *C. atrox* | 33.03492 | -107.3038 |
| *C. atrox* | 33.15569 | -107.2452 |
| *C. atrox* | 30.27907 | -97.52868 |
| *C. atrox* | 31.89852 | -106.4434 |
| *C. atrox* | 31.89615 | -106.5149 |
| *C. atrox* | 31.89305 | -106.5271 |
| *C. atrox* | 31.87172 | -106.0489 |
| *C. atrox* | 31.90673 | -106.0483 |
| *C. atrox* | 33.43481 | -114.7322 |
| *C. atrox* | 31.960928 | -108.1603 |
| *C. atrox* | 31.83554 | -106.0541 |
| *C. atrox* | 30.75593 | -105.0041 |
| *C. atrox* | 31.5089 | -105.3514 |
| *C. atrox* | 31.87858 | -104.8223 |
| *C. atrox* | 31.83114 | -104.8288 |
| *C. atrox* | 28.71289 | -98.247 |
| *C. atrox* | 31.818672 | -108.0149 |
| *C. atrox* | 31.94486 | -108.8381 |
| *C. atrox* | 31.81032 | -110.9068 |
| *C. atrox* | 31.78475 | -106.5851 |
| *C. atrox* | 31.7646 | -106.0306 |
| *C. atrox* | 31.68283 | -106.1667 |
| *C. atrox* | 30.9495 | -103.5895 |
| *C. atrox* | 32.53557 | -106.9908 |
| *C. atrox* | 32.89063 | -109.763 |
| *C. atrox* | 28.21622 | -96.98818 |
| *C. atrox* | 31.90863 | -106.0511 |
| *C. atrox* | 32.0865 | -105.093 |
| *C. atrox* | 30.99847 | -105.5442 |
| *C. atrox* | 28.46013 | -98.43758 |
| *C. atrox* | 32.49197 | -99.54212 |
| *C. atrox* | 34.19557 | -112.8128 |
| *C. atrox* | 30.5388 | -104.2609 |
| *C. atrox* | 31.82941 | -109.0356 |
| *C. atrox* | 31.89422 | -106.0483 |
| *C. atrox* | 31.49905 | -110.7021 |
| *C. atrox* | 30.7768 | -105.0162 |
| *C. atrox* | 32.93917 | -99.20052 |
| *C. atrox* | 33.72123 | -97.53738 |
| *C. atrox* | 30.85525 | -100.572 |
| *C. atrox* | 34.047 | -113.0628 |
| *C. atrox* | 31.9782 | -108.508 |
| *C. atrox* | 31.86858 | -106.0562 |
| *C. atrox* | 32.72228 | -111.1089 |
| *C. atrox* | 33.9757 | -103.141 |
| *C. atrox* | 29.80528 | -104.3054 |
| *C. atrox* | 30.1195 | -103.0817 |
| *C. atrox* | 30.41275 | -103.6898 |
| *C. atrox* | 30.05407 | -103.5799 |
| *C. atrox* | 30.03578 | -103.2814 |
| *C. atrox* | 29.67255 | -103.1281 |
| *C. atrox* | 29.94675 | -103.5821 |
| *C. atrox* | 29.83173 | -103.5767 |
| *C. atrox* | 30.14357 | -104.147 |
| *C. atrox* | 29.87957 | -104.2773 |
| *C. atrox* | 29.89648 | -104.2733 |
| *C. atrox* | 29.59543 | -104.4298 |
| *C. atrox* | 30.92376 | -105.0503 |
| *C. atrox* | 30.01938 | -103.5724 |
| *C. atrox* | 29.6508 | -103.5825 |
| *C. atrox* | 30.92641 | -105.0028 |
| *C. atrox* | 31.54973 | -110.7394 |
| *C. atrox* | 32.69633 | -109.0493 |
| *C. atrox* | 29.81283 | -103.5762 |
| *C. atrox* | 31.79403 | -111.4516 |
| *C. atrox* | 32.21209 | -106.4782 |
| *C. atrox* | 32.40223 | -106.2286 |
| *C. atrox* | 31.89481 | -104.8051 |
| *C. atrox* | 31.9355 | -106.6002 |
| *C. atrox* | 31.77843 | -106.5049 |
| *C. atrox* | 30.09093 | -103.2635 |
| *C. atrox* | 30.69817 | -104.6384 |
| *C. atrox* | 30.49801 | -104.3849 |
| *C. atrox* | 29.66912 | -103.1232 |
| *C. atrox* | 30.52733 | -104.217 |
| *C. atrox* | 31.66607 | -111.2349 |
| *C. atrox* | 31.84207 | -106.0644 |
| *C. atrox* | 31.766666 | -106.5 |
| *C. atrox* | 31.50989 | -105.3521 |
| *C. atrox* | 31.0066 | -101.0273 |
| *C. atrox* | 31.91856 | -106.0437 |
| *C. atrox* | 31.43 | -106.46 |
| *C. atrox* | 31.51967 | -106.1333 |
| *C. atrox* | 31.81363 | -106.2128 |
| *C. atrox* | 31.68692 | -105.9995 |
| *C. atrox* | 31.95311 | -108.7726 |
| *C. atrox* | 31.92522 | -108.3561 |
| *C. atrox* | 31.86594 | -106.4685 |
| *C. atrox* | 31.68429 | -105.9483 |
| *C. atrox* | 31.61685 | -106.0016 |
| *C. atrox* | 30.85726 | -105.3642 |
| *C. atrox* | 31.907 | -106.5722 |
| *C. atrox* | 32.422375 | -104.4471 |
| *C. atrox* | 31.01 | -106.49 |
| *C. atrox* | 31.7432 | -106.0303 |
| *C. atrox* | 31.80857 | -106.0579 |
| *C. atrox* | 31.78875 | -106.0426 |
| *C. atrox* | 29.97 | -106.37 |
| *C. atrox* | 24.21 | -101.45 |
| *C. atrox* | 24.63333 | -103.7 |
| *C. atrox* | 26.24 | -103.82 |
| *C. atrox* | 24.74 | -103.83 |
| *C. atrox* | 24.75 | -103.8 |
| *C. atrox* | 31.78328 | -106.0394 |
| *C. atrox* | 32.5681 | -105.7377 |
| *C. atrox* | 31.90217 | -106.4568 |
| *C. atrox* | 30.82627 | -102.8358 |
| *C. atrox* | 31.8206 | -106.0673 |
| *C. atrox* | 31.83238 | -106.0726 |
| *C. atrox* | 31.83383 | -106.0646 |
| *C. atrox* | 31.31667 | -106.4833 |
| *C. atrox* | 31.81722 | -106.0639 |
| *C. atrox* | 31.73261 | -105.4794 |
| *C. atrox* | 31.78122 | -105.4824 |
| *C. atrox* | 31.8305 | -106.0963 |
| *C. atrox* | 31.72922 | -106.0229 |
| *C. atrox* | 31.90753 | -106.5267 |
| *C. atrox* | 31.90082 | -106.0482 |
| *C. atrox* | 32.43083 | -106.5806 |
| *C. atrox* | 32.011826 | -106.5372 |
| *C. atrox* | 30.6059 | -104.1818 |
| *C. atrox* | 30.93079 | -105.0035 |
| *C. atrox* | 31.77246 | -106.0299 |
| *C. atrox* | 33.400402 | -105.9532 |
| *C. atrox* | 32.06944 | -107.0575 |
| *C. atrox* | 29.43207 | -104.1823 |
| *C. atrox* | 30.98352 | -105.5134 |
| *C. atrox* | 32.995 | -105.989 |
| *C. atrox* | 29.32523 | -103.5761 |
| *C. atrox* | 31.99076 | -106.4412 |
| *C. atrox* | 31.9325 | -104.4967 |
| *C. atrox* | 29.89274 | -98.62479 |
| *C. atrox* | 29.40483 | -98.72624 |
| *C. atrox* | 32.09249 | -108.3231 |
| *C. atrox* | 31.33377 | -104.8222 |
| *C. atrox* | 29.75443 | -100.7418 |
| *C. atrox* | 30.17 | -106.44 |
| *C. atrox* | 31.46 | -106.49 |
| *C. atrox* | 30.99 | -106.48 |
| *C. atrox* | 29.98 | -106.4 |
| *C. atrox* | 24.24 | -101.48 |
| *C. atrox* | 24.63 | -103.69 |
| *C. atrox* | 30.87 | -106.5 |
| *C. atrox* | 26.23 | -103.81 |
| *C. atrox* | 24.64194 | -103.6958 |
| *C. atrox* | 25.66 | -102.23 |
| *C. atrox* | 24.74 | -103.79 |
| *C. atrox* | 31.3 | -106.48 |
| *C. atrox* | 31.42 | -108.35 |
| *C. atrox* | 31.350134 | -108.319 |
| *C. atrox* | 31.45 | -108.23 |
| *C. atrox* | 31.41 | -108.29 |
| *C. atrox* | 32.15 | -107.68 |
| *C. atrox* | 32.03 | -104.06 |
| *C. atrox* | 32.723 | -108.711 |
| *C. atrox* | 32.028275 | -104.0406 |
| *C. atrox* | 33.18578 | -107.1741 |
| *C. atrox* | 32.07 | -108.997 |
| *C. atrox* | 32.13 | -108.94 |
| *C. atrox* | 32.1528 | -107.1269 |
| *C. atrox* | 32.2242 | -111.1586 |
| *C. atrox* | 31.7781 | -110.8183 |
| *C. atrox* | 32.2167 | -110.6667 |
| *C. atrox* | 31.7511 | -103.1594 |
| *C. atrox* | 29.9333 | -103.2442 |
| *C. atrox* | 29.92568 | -103.2604 |
| *C. atrox* | 31.912117 | -109.1308 |
| *C. basiliscus* | 21.57 | -105.23 |
| *C. basiliscus* | 20.51 | -105.3 |
| *C. basiliscus* | 19.19 | -103.82 |
| *C. basiliscus* | 19.29 | -103.65 |
| *C. basiliscus* | 23.19 | -106.19 |
| *C. basiliscus* | 23.08 | -106.15 |
| *C. basiliscus* | 19.716667 | -104.7 |
| *C. basiliscus* | 21.59 | -105.21 |
| *C. basiliscus* | 21.31 | -104.92 |
| *C. basiliscus* | 23.13 | -105.87 |
| *C. basiliscus* | 27.02 | -108.93 |
| *C. basiliscus* | 21.85 | -105.14 |
| *C. basiliscus* | 21.53 | -105.04 |
| *C. basiliscus* | 25.07 | -107.45 |
| *C. basiliscus* | 25.02 | -107.43 |
| *C. basiliscus* | 25.21 | -107.54 |
| *C. basiliscus* | 24.53 | -107.18 |
| *C. basiliscus* | 23.43 | -105.83 |
| *C. basiliscus* | 23.15 | -105.87 |
| *C. basiliscus* | 23.31 | -105.96 |
| *C. basiliscus* | 25.32 | -108.23 |
| *C. basiliscus* | 25.48 | -108.19 |
| *C. basiliscus* | 25.5 | -108.08 |
| *C. basiliscus* | 27.05 | -109.01 |
| *C. basiliscus* | 27.04 | -108.96 |
| *C. basiliscus* | 27.08083 | -109.0811 |
| *C. basiliscus* | 26.93 | -108.88 |
| *C. basiliscus* | 23.26 | -106.41 |
| *C. basiliscus* | 22.76 | -105.77 |
| *C. basiliscus* | 22.95 | -105.84 |
| *C. basiliscus* | 25.009068 | -107.5938 |
| *C. basiliscus* | 25.203102 | -107.8702 |
| *C. basiliscus* | 24.89 | -107.39 |
| *C. basiliscus* | 21.68 | -105.1 |
| *C. basiliscus* | 23.14 | -106.17 |
| *C. basiliscus* | 21.6 | -105.18 |
| *C. basiliscus* | 23.35 | -106.42 |
| *C. basiliscus* | 24.49 | -107.37 |
| *C. basiliscus* | 24.55 | -107.3 |
| *C. basiliscus* | 24.05 | -107.01 |
| *C. basiliscus* | 23.94 | -106.42 |
| *C. basiliscus* | 22.89 | -105.86 |
| *C. basiliscus* | 22.99 | -105.85 |
| *C. basiliscus* | 23.94 | -106.87 |
| *C. basiliscus* | 23.53 | -105.84 |
| *C. basiliscus* | 22.533333 | -105.7333 |
| *C. basiliscus* | 25.56 | -108.46 |
| *C. basiliscus* | 23.43 | -106.34 |
| *C. basiliscus* | 20.148632 | -105.3258 |
| *C. basiliscus* | 21.53944 | -105.2125 |
| *C. basiliscus* | 23.01612 | -105.9009 |
| *C. basiliscus* | 23.748838 | -106.5948 |
| *C. basiliscus* | 26.794298 | -109.3431 |
| *C. basiliscus* | 21.020435 | -104.2248 |
| *C. basiliscus* | 20.04 | -105.32 |
| *C. basiliscus* | 21.54 | -105.21 |
| *C. basiliscus* | 22.86 | -106.05 |
| *C. basiliscus* | 23.77 | -106.6 |
| *C. basiliscus* | 26.88 | -109.2 |
| *C. basiliscus* | 21.06 | -104.19 |
| *C. basiliscus* | 21.2025 | -105.0914 |
| *C. basiliscus* | 21.540596 | -105.2851 |
| *C. basiliscus* | 23.25 | -106.2 |
| *C. basiliscus* | 27.02 | -108.61 |
| *C. basiliscus* | 26.9 | -108.6833 |
| *C. basiliscus* | 19.241 | -103.728 |
| *C. basiliscus* | 24.8 | -107.38 |
| *C. basiliscus* | 24.93 | -107.39 |
| *C. basiliscus* | 22.72 | -105.76 |
| *C. basiliscus* | 26.52368 | -108.5683 |
| *C. basiliscus* | 21.67 | -105.11 |
| *C. basiliscus* | 21.55 | -104.96 |
| *C. basiliscus* | 23.47 | -106.42 |
| *C. basiliscus* | 24.63 | -107.39 |
| *C. basiliscus* | 26.23 | -108.99 |
| *C. basiliscus* | 27.02 | -109.06 |
| *C. basiliscus* | 19.08 | -103.86 |
| *C. basiliscus* | 21.17806 | -105.1367 |
| *C. basiliscus* | 22.35 | -105.31 |
| *C. basiliscus* | 23.29 | -105.95 |
| *C. basiliscus* | 18.80556 | -102.925 |
| *C. basiliscus* | 23.71 | -106.43 |
| *C. basiliscus* | 24.32361 | -107.3678 |
| *C. basiliscus* | 21.54 | -105.02 |
| *C. basiliscus* | 21.54 | -105 |
| *C. basiliscus* | 21.54 | -104.99 |
| *C. basiliscus* | 21.54 | -105.13 |
| *C. basiliscus* | 21.54 | -105.12 |
| *C. basiliscus* | 21.95 | -105.21 |
| *C. basiliscus* | 27 | -108.88 |
| *C. basiliscus* | 25.26 | -107.7 |
| *C. basiliscus* | 25.06 | -107.38 |
| *C. basiliscus* | 25.31 | -107.39 |
| *C. basiliscus* | 25.46 | -107.39 |
| *C. basiliscus* | 25.48 | -107.38 |
| *C. basiliscus* | 25.64 | -107.4 |
| *C. basiliscus* | 25.41 | -108.41 |
| *C. basiliscus* | 24.05 | -106.89 |
| *C. basiliscus* | 25.68 | -108.07 |
| *C. basiliscus* | 23.8 | -106.41 |
| *C. basiliscus* | 23.65 | -106.42 |
| *C. basiliscus* | 24.14 | -107.24 |
| *C. basiliscus* | 25.13 | -108.09 |
| *C. basiliscus* | 22.41 | -105.35 |
| *C. basiliscus* | 22.51 | -105.37 |
| *C. basiliscus* | 21.54 | -105.08 |
| *C. basiliscus* | 18.75 | -103.16 |
| *C. basiliscus* | 18.77306 | -103.1394 |
| *C. basiliscus* | 23.49 | -106.48 |
| *C. basiliscus* | 23.44 | -106.46 |
| *C. basiliscus* | 23.5 | -106.48 |
| *C. basiliscus* | 27.02 | -109.01 |
| *C. basiliscus* | 27.02 | -109.09 |
| *C. basiliscus* | 27 | -108.91 |
| *C. basiliscus* | 21.34 | -104.6 |
| *C. basiliscus* | 19.24306 | -103.7306 |
| *C. basiliscus* | 19.16 | -103.91 |
| *C. basiliscus* | 19.29 | -103.76 |
| *C. basiliscus* | 19.05 | -103.82 |
| *C. basiliscus* | 23.38 | -106.43 |
| *C. basiliscus* | 23.45 | -105.85 |
| *C. basiliscus* | 23.49 | -105.84 |
| *C. basiliscus* | 23.38 | -106.41 |
| *C. basiliscus* | 23.52 | -105.87 |
| *C. basiliscus* | 23.56 | -105.76 |
| *C. cerastes* | 32.969334 | -116.3118 |
| *C. cerastes* | 36.635 | -117.545 |
| *C. cerastes* | 28.82 | -111.94 |
| *C. cerastes* | 34.807 | -115.3312 |
| *C. cerastes* | 33.29582 | -116.3812 |
| *C. cerastes* | 36.621284 | -117.1678 |
| *C. cerastes* | 35.39493 | -116.1273 |
| *C. cerastes* | 30.99542 | -114.8373 |
| *C. cerastes* | 33.296867 | -116.3824 |
| *C. cerastes* | 32.5975 | -115.643 |
| *C. cerastes* | 34.927116 | -114.6519 |
| *C. cerastes* | 34.441246 | -115.7246 |
| *C. cerastes* | 33.15465 | -116.1917 |
| *C. cerastes* | 33.339424 | -116.2495 |
| *C. cerastes* | 32.976734 | -116.4156 |
| *C. cerastes* | 33.300705 | -116.3137 |
| *C. cerastes* | 33.1524 | -116.1801 |
| *C. cerastes* | 33.3007 | -116.3231 |
| *C. cerastes* | 33.303917 | -116.2037 |
| *C. cerastes* | 32.97378 | -115.1845 |
| *C. cerastes* | 35.167282 | -115.8278 |
| *C. cerastes* | 33.3131 | -116.3677 |
| *C. cerastes* | 32.83463 | -114.6389 |
| *C. cerastes* | 34.657665 | -115.6803 |
| *C. cerastes* | 34.588783 | -115.6463 |
| *C. cerastes* | 36.450554 | -116.8508 |
| *C. cerastes* | 36.286385 | -117.1648 |
| *C. cerastes* | 36.078377 | -116.5358 |
| *C. cerastes* | 37.212017 | -118.2392 |
| *C. cerastes* | 33.237667 | -116.1114 |
| *C. cerastes* | 37.638283 | -118.3976 |
| *C. cerastes* | 33.125683 | -116.0438 |
| *C. cerastes* | 33.12575 | -115.9819 |
| *C. cerastes* | 33.125698 | -115.9972 |
| *C. cerastes* | 34.94194 | -114.8232 |
| *C. cerastes* | 34.60685 | -115.4292 |
| *C. cerastes* | 33.234722 | -111.932 |
| *C. cerastes* | 35.21207 | -116.7434 |
| *C. cerastes* | 35.016388 | -116.8969 |
| *C. cerastes* | 34.04685 | -116.5808 |
| *C. cerastes* | 34.738018 | -115.6632 |
| *C. cerastes* | 33.503056 | -114.9229 |
| *C. cerastes* | 37.04933 | -113.974 |
| *C. cerastes* | 32.97447 | -115.3968 |
| *C. cerastes* | 34.30773 | -117.3318 |
| *C. cerastes* | 32.725346 | -114.6244 |
| *C. cerastes* | 32.984818 | -113.3234 |
| *C. cerastes* | 34.948383 | -116.0169 |
| *C. cerastes* | 32.980835 | -116.4258 |
| *C. cerastes* | 34.923035 | -117.8723 |
| *C. cerastes* | 33.76235 | -116.442 |
| *C. cerastes* | 33.062458 | -115.7286 |
| *C. cerastes* | 35.28 | -116.6275 |
| *C. cerastes* | 31.59 | -112.78 |
| *C. cerastes* | 31.55 | -115.0667 |
| *C. cerastes* | 31.71 | -113.31 |
| *C. cerastes* | 35.859 | -116.3084 |
| *C. cerastes* | 36.82921 | -116.8836 |
| *C. cerastes* | 36.712692 | -116.9736 |
| *C. cerastes* | 36.8874 | -116.8087 |
| *C. cerastes* | 33.335163 | -116.2808 |
| *C. cerastes* | 35.972015 | -116.2702 |
| *C. cerastes* | 36.7454 | -117.1373 |
| *C. cerastes* | 28.37 | -111.46 |
| *C. cerastes* | 29.93333 | -112.5833 |
| *C. cerastes* | 28.93 | -111.88 |
| *C. cerastes* | 31.033333 | -115.2333 |
| *C. cerastes* | 31.016666 | -115.2167 |
| *C. cerastes* | 32.705486 | -114.6207 |
| *C. cerastes* | 34.95077 | -114.8263 |
| *C. cerastes* | 31.416668 | -112.85 |
| *C. cerastes* | 31.85 | -114.4333 |
| *C. cerastes* | 34.2451 | -114.6437 |
| *C. cerastes* | 33.84405 | -114.773 |
| *C. cerastes* | 33.74943 | -114.5164 |
| *C. cerastes* | 33.256874 | -116.3998 |
| *C. cerastes* | 33.082333 | -116.1078 |
| *C. cerastes* | 33.26585 | -116.4 |
| *C. cerastes* | 35.809914 | -114.8836 |
| *C. cerastes* | 36.09164 | -114.9087 |
| *C. cerastes* | 35.961132 | -114.896 |
| *C. cerastes* | 36.89972 | -116.7528 |
| *C. cerastes* | 37.154007 | -117.165 |
| *C. cerastes* | 37.120377 | -117.2057 |
| *C. cerastes* | 37.122482 | -117.2031 |
| *C. cerastes* | 37.18485 | -117.144 |
| *C. cerastes* | 37.172142 | -117.151 |
| *C. cerastes* | 34.95221 | -116.644 |
| *C. cerastes* | 33.1442 | -116.1236 |
| *C. cerastes* | 32.943314 | -112.7353 |
| *C. cerastes* | 35.659367 | -116.2958 |
| *C. cerastes* | 36.519337 | -117.9005 |
| *C. cerastes* | 33.37641 | -112.5796 |
| *C. cerastes* | 34.083015 | -114.8506 |
| *C. cerastes* | 32.195 | -115.807 |
| *C. cerastes* | 34.07363 | -115.1286 |
| *C. cerastes* | 34.09068 | -114.9986 |
| *C. cerastes* | 35.14218 | -116.1042 |
| *C. cerastes* | 35.01099 | -115.4734 |
| *C. cerastes* | 31.58 | -113.14 |
| *C. cerastes* | 31.86 | -113.51 |
| *C. cerastes* | 33.672253 | -112.0311 |
| *C. cerastes* | 37.06528 | -113.8886 |
| *C. cerastes* | 32.670303 | -114.477 |
| *C. cerastes* | 33.10887 | -115.835 |
| *C. cerastes* | 33.02545 | -114.9012 |
| *C. cerastes* | 35.05308 | -118.0517 |
| *C. cerastes* | 34.966457 | -118.1594 |
| *C. cerastes* | 33.82763 | -115.9292 |
| *C. cerastes* | 33.76307 | -115.9292 |
| *C. cerastes* | 33.73633 | -115.8093 |
| *C. cerastes* | 33.64967 | -115.8319 |
| *C. cerastes* | 33.78027 | -115.8249 |
| *C. cerastes* | 33.78897 | -115.8249 |
| *C. cerastes* | 33.80789 | -115.773 |
| *C. cerastes* | 34.34013 | -115.7953 |
| *C. cerastes* | 33.79865 | -115.784 |
| *C. cerastes* | 33.67614 | -117.0058 |
| *C. cerastes* | 34.91668 | -115.9175 |
| *C. cerastes* | 33.79752 | -115.7852 |
| *C. cerastes* | 33.87871 | -115.7353 |
| *C. cerastes* | 33.89903 | -115.7264 |
| *C. cerastes* | 33.84948 | -115.7493 |
| *C. cerastes* | 33.85222 | -115.7481 |
| *C. cerastes* | 34.07401 | -116.5448 |
| *C. cerastes* | 34.08296 | -116.531 |
| *C. cerastes* | 33.80412 | -116.1903 |
| *C. cerastes* | 33.70007 | -116.2381 |
| *C. cerastes* | 33.83008 | -116.2234 |
| *C. cerastes* | 33.7723 | -116.1739 |
| *C. cerastes* | 33.71497 | -116.1725 |
| *C. cerastes* | 33.73477 | -116.2119 |
| *C. cerastes* | 33.75103 | -116.2046 |
| *C. cerastes* | 33.692917 | -117.0013 |
| *C. cerastes* | 33.81954 | -116.7335 |
| *C. cerastes* | 33.92318 | -116.6863 |
| *C. cerastes* | 33.92118 | -116.7539 |
| *C. cerastes* | 33.87577 | -116.6732 |
| *C. cerastes* | 33.3334 | -115.8342 |
| *C. cerastes* | 33.59014 | -115.9625 |
| *C. cerastes* | 33.52724 | -115.9419 |
| *C. cerastes* | 33.58838 | -116.9316 |
| *C. cerastes* | 33.61228 | -115.9169 |
| *C. cerastes* | 33.67289 | -115.5871 |
| *C. cerastes* | 33.99601 | -115.2379 |
| *C. cerastes* | 34.0419 | -115.226 |
| *C. cerastes* | 34.172775 | -116.0605 |
| *C. cerastes* | 34.0781 | -116.0359 |
| *C. cerastes* | 34.065617 | -115.3221 |
| *C. cerastes* | 34.092197 | -115.408 |
| *C. cerastes* | 34.096622 | -115.5369 |
| *C. cerastes* | 34.098965 | -115.6308 |
| *C. cerastes* | 34.135536 | -116.2617 |
| *C. cerastes* | 34.25386 | -116.4384 |
| *C. cerastes* | 34.327003 | -116.4831 |
| *C. cerastes* | 34.105965 | -116.1032 |
| *C. cerastes* | 34.061817 | -116.3343 |
| *C. cerastes* | 34.05589 | -116.0254 |
| *C. cerastes* | 34.27024 | -116.3896 |
| *C. cerastes* | 35.557148 | -116.1934 |
| *C. cerastes* | 34.60213 | -117.6496 |
| *C. cerastes* | 34.146446 | -116.417 |
| *C. cerastes* | 34.475277 | -116.9796 |
| *C. cerastes* | 34.86507 | -117.1964 |
| *C. cerastes* | 35.02738 | -117.1008 |
| *C. cerastes* | 33.28272 | -116.1336 |
| *C. cerastes* | 33.28554 | -116.3578 |
| *C. cerastes* | 33.27704 | -116.3995 |
| *C. cerastes* | 33.13638 | -116.2177 |
| *C. cerastes* | 32.87568 | -116.1328 |
| *C. cerastes* | 37.043278 | -116.0455 |
| *C. cerastes* | 37.11658 | -113.574 |
| *C. cerastes* | 31.016666 | -114.8333 |
| *C. cerastes* | 30.75 | -114.7833 |
| *C. cerastes* | 30.87637 | -114.7401 |
| *C. cerastes* | 31.04249 | -114.8355 |
| *C. cerastes* | 29.78333 | -114.3833 |
| *C. cerastes* | 32.47 | -114.74 |
| *C. cerastes* | 32.46 | -114.72 |
| *C. cerastes* | 32.07 | -113.71 |
| *C. cerastes* | 32.44 | -114.64 |
| *C. cerastes* | 32.43 | -114.62 |
| *C. cerastes* | 32.39 | -114.47 |
| *C. cerastes* | 32.38 | -114.44 |
| *C. cerastes* | 32.37 | -114.42 |
| *C. cerastes* | 32.36 | -114.41 |
| *C. cerastes* | 32.29 | -114.16 |
| *C. cerastes* | 32.42 | -114.6 |
| *C. cerastes* | 32.41 | -114.55 |
| *C. cerastes* | 31.99 | -113.32 |
| *C. cerastes* | 32.24 | -114.06 |
| *C. cerastes* | 28.83 | -111.82 |
| *C. cerastes* | 34.096664 | -115.0301 |
| *C. cerastes* | 33.86443 | -116.2156 |
| *C. cerastes* | 34.602013 | -117.6104 |
| *C. cerastes* | 33.30344 | -116.248 |
| *C. cerastes* | 34.99211 | -115.6903 |
| *C. cerastes* | 34.394524 | -116.7078 |
| *C. cerastes* | 36.7987 | -116.3006 |
| *C. cerastes* | 36.84402 | -116.2249 |
| *C. cerastes* | 36.78249 | -116.1486 |
| *C. cerastes* | 36.65943 | -116.0858 |
| *C. cerastes* | 36.81325 | -116.1867 |
| *C. cerastes* | 36.2576 | -117.9933 |
| *C. cerastes* | 37.10415 | -113.5841 |
| *C. cerastes* | 34.60558 | -118.0397 |
| *C. cerastes* | 34.60168 | -117.5251 |
| *C. cerastes* | 34.43276 | -117.5615 |
| *C. cerastes* | 35.00655 | -117.198 |
| *C. cerastes* | 34.13796 | -114.5279 |
| *C. cerastes* | 33.58467 | -115.9487 |
| *C. cerastes* | 33.71522 | -116.5454 |
| *C. cerastes* | 33.6746 | -115.8018 |
| *C. cerastes* | 35.05308 | -118.0729 |
| *C. cerastes* | 33.33281 | -116.2924 |
| *C. cerastes* | 35.9689 | -116.6298 |
| *C. cerastes* | 35.56709 | -116.2042 |
| *C. cerastes* | 33.25325 | -115.944 |
| *C. cerastes* | 33.6853 | -116.2504 |
| *C. cerastes* | 36.03979 | -114.7666 |
| *C. cerastes* | 32.55489 | -115.762 |
| *C. cerastes* | 34.92997 | -116.6618 |
| *C. cerastes* | 31.67 | -113.32 |
| *C. cerastes* | 32.87281 | -114.3521 |
| *C. cerastes* | 34.8879 | -114.5249 |
| *C. cerastes* | 33.6552 | -115.8024 |
| *C. cerastes* | 34.93402 | -117.198 |
| *C. cerastes* | 36.65943 | -115.9958 |
| *C. cerastes* | 37.23954 | -115.9958 |
| *C. cerastes* | 34.52863 | -117.2872 |
| *C. cerastes* | 34.323193 | -116.5601 |
| *C. cerastes* | 34.365902 | -117.3387 |
| *C. cerastes* | 34.60031 | -117.8483 |
| *C. cerastes* | 34.579155 | -118.1151 |
| *C. cerastes* | 33.84229 | -114.5351 |
| *C. cerastes* | 34.21832 | -116.0492 |
| *C. cerastes* | 32.7487 | -114.7657 |
| *C. cerastes* | 33.13477 | -116.2999 |
| *C. cerastes* | 32.34 | -114.3 |
| *C. cerastes* | 32.98302 | -115.0711 |
| *C. cerastes* | 34.5565 | -117.7217 |
| *C. cerastes* | 34.02455 | -116.6363 |
| *C. cerastes* | 33.74244 | -116.1723 |
| *C. cerastes* | 33.44834 | -112.074 |
| *C. cerastes* | 35.47863 | -117.5809 |
| *C. cerastes* | 35.50039 | -117.5898 |
| *C. cerastes* | 35.34082 | -117.4481 |
| *C. cerastes* | 35.55824 | -117.7777 |
| *C. cerastes* | 35.12562 | -118.1611 |
| *C. cerastes* | 34.135067 | -116.2108 |
| *C. cerastes* | 34.13659 | -115.9311 |
| *C. cerastes* | 35.32082 | -114.9147 |
| *C. cerastes* | 36.3379 | -116.1647 |
| *C. cerastes* | 32.03 | -113.47 |
| *C. cerastes* | 34.44378 | -116.9451 |
| *C. cerastes* | 32.00528 | -113.3672 |
| *C. cerastes* | 32.44 | -114.66 |
| *C. cerastes* | 32.42 | -114.58 |
| *C. cerastes* | 32.45 | -114.69 |
| *C. cerastes* | 29.18 | -111.66 |
| *C. cerastes* | 29.15 | -111.58 |
| *C. cerastes* | 32.45 | -114.67 |
| *C. cerastes* | 29.18 | -111.69 |
| *C. cerastes* | 32.39 | -114.49 |
| *C. cerastes* | 32.46 | -114.7 |
| *C. cerastes* | 32.38 | -114.45 |
| *C. cerastes* | 31.85 | -112.89 |
| *C. cerastes* | 31.71 | -113.14 |
| *C. cerastes* | 31.78 | -113 |
| *C. cerastes* | 31.84 | -112.92 |
| *C. cerastes* | 31.81 | -112.96 |
| *C. cerastes* | 31.75 | -113.04 |
| *C. cerastes* | 31.71 | -113.16 |
| *C. cerastes* | 31.76 | -113.02 |
| *C. cerastes* | 32.12 | -113.81 |
| *C. cerastes* | 31.72 | -113.09 |
| *C. cerastes* | 32.12 | -113.83 |
| *C. cerastes* | 33.30958 | -114.8784 |
| *C. cerastes* | 33.03464 | -114.8361 |
| *C. cerastes* | 30.61 | -111.58 |
| *C. cerastes* | 31.64 | -113.33 |
| *C. cerastes* | 33.71864 | -116.3084 |
| *C. cerastes* | 31.94 | -113.33 |
| *C. cerastes* | 31.9 | -113 |
| *C. cerastes* | 32.29 | -114.15 |
| *C. cerastes* | 32.06 | -113.61 |
| *C. cerastes* | 31.83333 | -112.8667 |
| *C. cerastes* | 35.92465 | -115.6655 |
| *C. cerastes* | 33.58331 | -113.2634 |
| *C. cerastes* | 31.6727 | -110.8867 |
| *C. cerastes* | 33.1114 | -115.6617 |
| *C. cerastes* | 35.31384 | -118.0481 |
| *C. cerastes* | 34.84806 | -114.6133 |
| *C. cerastes* | 34.631237 | -117.8618 |
| *C. cerastes* | 35.612495 | -117.9148 |
| *C. cerastes* | 33.4526 | -112.9854 |
| *C. cerastes* | 34.64601 | -117.8595 |
| *C. cerastes* | 36.376488 | -117.5913 |
| *C. cerastes* | 32.9739 | -115.1812 |
| *C. cerastes* | 33.0575 | -115.7498 |
| *C. cerastes* | 33.0731 | -113.0111 |
| *C. cerastes* | 32.7091 | -115.0818 |
| *C. cerastes* | 35.65 | -117.97 |
| *C. cerastes* | 35.586494 | -118.0643 |
| *C. cerastes* | 35.532475 | -115.7056 |
| *C. cerastes* | 34.92753 | -115.4494 |
| *C. cerastes* | 35.80119 | -116.0937 |
| *C. cerastes* | 36.352535 | -117.294 |
| *C. cerastes* | 32.2452 | -112.7275 |
| *C. cerastes* | 33.1897 | -116.2957 |
| *C. cerastes* | 36.66056 | -116.1376 |
| *C. cerastes* | 36.057415 | -114.7455 |
| *C. cerastes* | 32.8671 | -113.1493 |
| *C. cerastes* | 32.7396 | -115.8232 |
| *C. cerastes* | 32.6793 | -115.712 |
| *C. cerastes* | 33.723206 | -116.2226 |
| *C. cerastes* | 36.293114 | -117.3829 |
| *C. cerastes* | 36.350334 | -117.5492 |
| *C. cerastes* | 36.358276 | -117.9018 |
| *C. cerastes* | 34.369373 | -116.6042 |
| *C. cerastes* | 34.36855 | -116.543 |
| *C. cerastes* | 34.30182 | -115.7061 |
| *C. cerastes* | 34.208668 | -115.7191 |
| *C. cerastes* | 34.80858 | -116.5647 |
| *C. cerastes* | 34.815212 | -116.604 |
| *C. cerastes* | 34.56947 | -115.8124 |
| *C. cerastes* | 33.714947 | -116.1723 |
| *C. cerastes* | 35.04233 | -114.6529 |
| *C. cerastes* | 36.51222 | -114.375 |
| *C. cerastes* | 36.72403 | -116.5285 |
| *C. cerastes* | 36.45467 | -114.3839 |
| *C. cerastes* | 36.67978 | -114.5683 |
| *C. cerastes* | 32.2092 | -111.2174 |
| *C. cerastes* | 34.766106 | -115.6334 |
| *C. cerastes* | 32.0021 | -111.9863 |
| *C. cerastes* | 37.18244 | -118.2592 |
| *C. cerastes* | 37.185688 | -118.251 |
| *C. cerastes* | 37.454227 | -117.897 |
| *C. cerastes* | 37.43657 | -117.8875 |
| *C. cerastes* | 36.046867 | -116.2935 |
| *C. cerastes* | 37.05515 | -118.2107 |
| *C. cerastes* | 36.57371 | -117.9945 |
| *C. cerastes* | 36.51952 | -117.8996 |
| *C. cerastes* | 37.682743 | -118.3857 |
| *C. cerastes* | 34.235485 | -115.7214 |
| *C. cerastes* | 35.768173 | -116.3225 |
| *C. cerastes* | 34.80335 | -115.3637 |
| *C. cerastes* | 34.720394 | -115.69 |
| *C. cerastes* | 36.80556 | -114.0664 |
| *C. cerastes* | 36.14 | -114.89 |
| *C. cerastes* | 36.08 | -115.45 |
| *C. cerastes* | 33.709435 | -115.2298 |
| *C. cerastes* | 34.561184 | -115.5815 |
| *C. cerastes* | 34.81118 | -115.6091 |
| *C. cerastes* | 34.5735 | -115.8313 |
| *C. cerastes* | 37.43394 | -117.8826 |
| *C. cerastes* | 34.58839 | -116.7861 |
| *C. cerastes* | 34.802307 | -116.8423 |
| *C. cerastes* | 32.32139 | -111.3214 |
| *C. cerastes* | 33.011806 | -112.3979 |
| *C. cerastes* | 33.004055 | -112.4189 |
| *C. cerastes* | 33.924194 | -113.237 |
| *C. cerastes* | 33.51433 | -113.8356 |
| *C. cerastes* | 33.531776 | -113.5825 |
| *C. cerastes* | 33.53753 | -113.5006 |
| *C. cerastes* | 34.120693 | -114.0654 |
| *C. cerastes* | 34.60603 | -116.7488 |
| *C. cerastes* | 34.60603 | -116.748 |
| *C. cerastes* | 33.263916 | -112.8332 |
| *C. cerastes* | 33.92636 | -113.2303 |
| *C. cerastes* | 33.85986 | -113.4226 |
| *C. cerastes* | 33.83439 | -113.4971 |
| *C. cerastes* | 33.98886 | -113.5827 |
| *C. cerastes* | 33.514416 | -113.7492 |
| *C. cerastes* | 34.557083 | -116.6889 |
| *C. cerastes* | 34.64672 | -116.7211 |
| *C. cerastes* | 34.530556 | -116.823 |
| *C. cerastes* | 34.540585 | -116.7172 |
| *C. cerastes* | 34.515278 | -116.776 |
| *C. cerastes* | 33.273224 | -113.0898 |
| *C. cerastes* | 33.288555 | -112.9489 |
| *C. cerastes* | 34.119167 | -113.9158 |
| *C. cerastes* | 35.085796 | -114.5234 |
| *C. cerastes* | 33.3058 | -115.8336 |
| *C. cerastes* | 33.6421 | -112.6903 |
| *C. cerastes* | 35.5272 | -115.7675 |
| *C. cerastes* | 34.98171 | -115.6902 |
| *C. cerastes* | 33.756187 | -116.4778 |
| *C. cerastes* | 34.99444 | -117.585 |
| *C. cerastes* | 33.592014 | -116.226 |
| *C. cerastes* | 36.65225 | -116.4391 |
| *C. cerastes* | 33.2506 | -115.8773 |
| *C. cerastes* | 33.93553 | -115.6895 |
| *C. cerastes* | 33.768833 | -115.8104 |
| *C. cerastes* | 34.050205 | -115.7536 |
| *C. cerastes* | 33.723255 | -116.1131 |
| *C. cerastes* | 32.811 | -115.379 |
| *C. cerastes* | 31.05 | -114.85 |
| *C. cerastes* | 36.87761 | -113.9325 |
| *C. cerastes* | 34.080124 | -114.9346 |
| *C. cerastes* | 33.93222 | -117.0295 |
| *C. cerastes* | 32.7757 | -114.3731 |
| *C. cerastes* | 34.12299 | -114.5092 |
| *C. cerastes* | 36.08273 | -115.0325 |
| *C. cerastes* | 35.69737 | -114.9189 |
| *C. cerastes* | 34.347893 | -117.4714 |
| *C. cerastes* | 33.69157 | -116.3994 |
| *C. cerastes* | 33.7648 | -116.3608 |
| *C. cerastes* | 32.25 | -112.7158 |
| *C. cerastes* | 36.01963 | -114.7812 |
| *C. cerastes* | 35.90682 | -114.92 |
| *C. cerastes* | 36.54333 | -114.2664 |
| *C. cerastes* | 33.1544 | -115.8788 |
| *C. cerastes* | 35.743507 | -117.9448 |
| *C. cerastes* | 33.92993 | -114.5402 |
| *C. cerastes* | 33.85796 | -114.593 |
| *C. cerastes* | 32.7966 | -114.3802 |
| *C. cerastes* | 32.6817 | -114.6236 |
| *C. cerastes* | 32.7261 | -114.7604 |
| *C. cerastes* | 35.64863 | -117.65 |
| *C. cerastes* | 33.70972 | -116.4005 |
| *C. cerastes* | 34.54389 | -115.7903 |
| *C. cerastes* | 32.4733 | -112.86 |
| *C. cerastes* | 34.34062 | -116.6628 |
| *C. cerastes* | 34.38836 | -116.802 |
| *C. cerastes* | 37.405216 | -118.3138 |
| *C. cerastes* | 36.45679 | -116.8656 |
| *C. cerastes* | 36.435104 | -116.7252 |
| *C. cerastes* | 36.200535 | -116.8666 |
| *C. cerastes* | 36.4478 | -116.8003 |
| *C. cerastes* | 36.56051 | -118.0508 |
| *C. cerastes* | 37.51008 | -118.3633 |
| *C. cerastes* | 34.114044 | -116.6059 |
| *C. cerastes* | 33.2162 | -112.0469 |
| *C. cerastes* | 33.77248 | -116.1731 |
| *C. cerastes* | 33.791264 | -116.6761 |
| *C. cerastes* | 34.59889 | -117.3246 |
| *C. cerastes* | 32.9144 | -115.072 |
| *C. cerastes* | 31.90763 | -112.9113 |
| *C. cerastes* | 31.8887 | -112.8117 |
| *C. cerastes* | 31.99535 | -113.3372 |
| *C. cerastes* | 36.83991 | -114.8237 |
| *C. cerastes* | 31 | -114.8667 |
| *C. cerastes* | 33.837944 | -116.2325 |
| *C. cerastes* | 34.783928 | -115.2045 |
| *C. cerastes* | 31.0275 | -114.8353 |
| *C. cerastes* | 31.89 | -112.87 |
| *C. cerastes* | 28.82278 | -111.9408 |
| *C. cerastes* | 31.91 | -112.95 |
| *C. cerastes* | 32.956535 | -112.9751 |
| *C. cerastes* | 32.7431 | -114.58 |
| *C. cerastes* | 34.77123 | -114.1943 |
| *C. cerastes* | 32.932407 | -112.804 |
| *C. cerastes* | 34.54363 | -115.5291 |
| *C. cerastes* | 28.83 | -111.89 |
| *C. cerastes* | 32.22442 | -112.7005 |
| *C. cerastes* | 32.324276 | -112.774 |
| *C. cerastes* | 32.289455 | -112.7506 |
| *C. cerastes* | 32.686775 | -112.8502 |
| *C. cerastes* | 34.62912 | -115.3973 |
| *C. cerastes* | 32.67661 | -115.7717 |
| *C. cerastes* | 32.67524 | -115.7955 |
| *C. cerastes* | 32.66903 | -115.8838 |
| *C. cerastes* | 34.579445 | -118.1156 |
| *C. cerastes* | 33.22889 | -116.2603 |
| *C. cerastes* | 32.7226 | -114.9146 |
| *C. cerastes* | 32.98995 | -116.4551 |
| *C. cerastes* | 33.31971 | -116.3502 |
| *C. cerastes* | 36.80523 | -114.0673 |
| *C. cerastes* | 32.81146 | -115.2777 |
| *C. cerastes* | 32.89343 | -112.5632 |
| *C. cerastes* | 31.44015 | -115.0524 |
| *C. cerastes* | 31.1444 | -114.9134 |
| *C. cerastes* | 31.09307 | -114.8906 |
| *C. cerastes* | 31.03333 | -114.85 |
| *C. cerastes* | 32.70808 | -115.0452 |
| *C. cerastes* | 32.70921 | -114.9426 |
| *C. cerastes* | 30.93365 | -114.7359 |
| *C. cerastes* | 29.77667 | -114.3733 |
| *C. cerastes* | 35.56679 | -117.7264 |
| *C. cerastes* | 32.70246 | -115.2718 |
| *C. cerastes* | 35.72384 | -117.6444 |
| *C. cerastes* | 32.7314 | -114.7492 |
| *C. cerastes* | 33.61228 | -114.9956 |
| *C. cerastes* | 33.38559 | -111.8772 |
| *C. cerastes* | 33.38892 | -116.4589 |
| *C. cerastes* | 33.66336 | -116.2931 |
| *C. cerastes* | 32.68389 | -114.6252 |
| *C. cerastes* | 34.81599 | -117.1908 |
| *C. cerastes* | 33.13531 | -116.2985 |
| *C. cerastes* | 33.42676 | -111.8252 |
| *C. cerastes* | 33.13892 | -116.3862 |
| *C. cerastes* | 34.60031 | -117.8483 |
| *C. cerastes* | 32.75344 | -114.7922 |
| *C. cerastes* | 32.62579 | -114.5915 |
| *C. cerastes* | 35.00704 | -117.7021 |
| *C. cerastes* | 36.11273 | -115.2301 |
| *C. cerastes* | 34.69342 | -117.4501 |
| *C. cerastes* | 32.75544 | -114.8584 |
| *C. cerastes* | 33.48669 | -111.8252 |
| *C. cerastes* | 32.74929 | -115.0346 |
| *C. cerastes* | 36.15232 | -114.4515 |
| *C. cerastes* | 34.91151 | -117.8388 |
| *C. cerastes* | 32.79313 | -115.6911 |
| *C. cerastes* | 33.84219 | -112.6232 |
| *C. cerastes* | 33.68917 | -115.2667 |
| *C. cerastes* | 32.69606 | -116.1839 |
| *C. cerastes* | 32.70926 | -115.0802 |
| *C. cerastes* | 34.53991 | -117.2622 |
| *C. cerastes* | 33.71585 | -116.6823 |
| *C. cerastes* | 33.10921 | -115.8353 |
| *C. cerastes* | 35.77454 | -117.8501 |
| *C. cerastes* | 32.84733 | -115.7572 |
| *C. cerastes* | 35.65174 | -117.6723 |
| *C. cerastes* | 32.90906 | -116.2333 |
| *C. cerastes* | 33.76898 | -116.0568 |
| *C. cerastes* | 34.85657 | -116.8408 |
| *C. cerastes* | 32.70989 | -115.1308 |
| *C. cerastes* | 32.81102 | -115.2087 |
| *C. cerastes* | 33.13794 | -116.0988 |
| *C. cerastes* | 33.88345 | -116.1688 |
| *C. cerastes* | 34.30565 | -117.0637 |
| *C. cerastes* | 34.52535 | -117.3987 |
| *C. cerastes* | 34.74245 | -117.326 |
| *C. cerastes* | 34.97831 | -117.5368 |
| *C. cerastes* | 33.129 | -116.4311 |
| *C. cerastes* | 36.08732 | -117.9618 |
| *C. cerastes* | 32.66557 | -114.3918 |
| *C. cerastes* | 33.34904 | -116.4006 |
| *C. cerastes* | 32.69827 | -114.5985 |
| *C. cerastes* | 33.13272 | -116.0827 |
| *C. cerastes* | 33.13374 | -116.3393 |
| *C. cerastes* | 34.1234 | -116.4132 |
| *C. cerastes* | 34.13487 | -116.3423 |
| *C. cerastes* | 34.22956 | -116.0589 |
| *C. cerastes* | 33.7205 | -116.1289 |
| *C. cerastes* | 30.84085 | -114.7436 |
| *C. cerastes* | 31.02883 | -114.8372 |
| *C. cerastes* | 31.04205 | -114.8353 |
| *C. cerastes* | 31.01667 | -114.8333 |
| *C. cerastes* | 32.9525 | -113.1739 |
| *C. cerastes* | 33.14293 | -116.2696 |
| *C. cerastes* | 38.01057 | -117.2556 |
| *C. cerastes* | 33.25657 | -116.2776 |
| *C. cerastes* | 32.79547 | -116.1092 |
| *C. cerastes* | 33.25681 | -116.2678 |
| *C. cerastes* | 32.70945 | -115.1145 |
| *C. cerastes* | 33.14664 | -116.1409 |
| *C. cerastes* | 32.89811 | -116.2298 |
| *C. cerastes* | 32.67493 | -114.4949 |
| *C. cerastes* | 31.97 | -113.33 |
| *C. cerastes* | 33.09646 | -116.44 |
| *C. cerastes* | 33.51879 | -112.0228 |
| *C. cerastes* | 32.98044 | -111.463 |
| *C. cerastes* | 35.01236 | -117.9154 |
| *C. cerastes* | 33.22089 | -116.3793 |
| *C. cerastes* | 33.37436 | -116.4284 |
| *C. cerastes* | 28.68 | -110.99 |
| *C. cerastes* | 28.89 | -110.13 |
| *C. cerastes* | 28.72 | -111.28 |
| *C. cerastes* | 28.84 | -111.18 |
| *C. cerastes* | 28.98334 | -112.2879 |
| *C. cerastes* | 34.13535 | -116.0711 |
| *C. cerastes* | 34.82703 | -116.6876 |
| *C. cerastes* | 34.01774 | -113.0997 |
| *C. cerastes* | 36.17419 | -115.1539 |
| *C. cerastes* | 33.19924 | -116.5987 |
| *C. cerastes* | 31.11116 | -114.8992 |
| *C. cerastes* | 32.70304 | -112.8476 |
| *C. cerastes* | 32.72385 | -112.7206 |
| *C. cerastes* | 32.66628 | -115.9924 |
| *C. cerastes* | 32.99773 | -115.0701 |
| *C. cerastes* | 32.34 | -114.32 |
| *C. cerastes* | 32.06667 | -113.6833 |
| *C. cerastes* | 32.46 | -114.77 |
| *C. cerastes* | 32.41 | -114.53 |
| *C. cerastes* | 32.35 | -114.3833 |
| *C. cerastes* | 32.9506 | -116.2126 |
| *C. cerastes* | 32.73857 | -116.0465 |
| *C. cerastes* | 32.65139 | -115.8009 |
| *C. cerastes* | 33.01087 | -116.1676 |
| *C. cerastes* | 32.76013 | -116.0654 |
| *C. cerastes* | 32.74928 | -116.0339 |
| *C. cerastes* | 32.78228 | -116.1035 |
| *C. cerastes* | 33.23369 | -115.7326 |
| *C. cerastes* | 32.81913 | -116.1491 |
| *C. cerastes* | 32.85872 | -116.2079 |
| *C. cerastes* | 33.09533 | -116.1135 |
| *C. cerastes* | 30.35 | -114.64 |
| *C. cerastes* | 33.1795 | -111.3513 |
| *C. cerastes* | 32.1476 | -112.7657 |
| *C. cerastes* | 35.998 | -114.5073 |
| *C. cerastes* | 32.836 | -114.3669 |
| *C. cerastes* | 32.8565 | -114.3609 |
| *C. cerastes* | 33.3205 | -114.2169 |
| *C. cerastes* | 33.6183 | -114.217 |
| *C. cerastes* | 32.7828 | -116.0948 |
| *C. cerastes* | 32.7487 | -116.032 |
| *C. cerastes* | 32.8312 | -116.1749 |
| *C. cerastes* | 33.31962 | -116.0581 |
| *C. cerastes* | 32.79223 | -116.1069 |
| *C. cerastes* | 33.28614 | -116.2986 |
| *C. cerastes* | 33.32266 | -116.0176 |
| *C. cerastes* | 36.75977 | -114.3649 |
| *C. cerastes* | 33.60828 | -114.8911 |
| *C. cerastes* | 32.73257 | -114.902 |
| *C. cerastes* | 37.36152 | -118.3943 |
| *C. cerastes* | 33.44834 | -112.3856 |
| *C. cerastes* | 31.37 | -113.5 |
| *C. cerastes* | 31.39 | -113.48 |
| *C. cerastes* | 31.44 | -113.46 |
| *C. cerastes* | 31.5 | -113.43 |
| *C. cerastes* | 31.63 | -113.14 |
| *C. cerastes* | 28.84 | -111.86 |
| *C. cerastes* | 28.83 | -111.84 |
| *C. cerastes* | 28.83 | -111.79 |
| *C. cerastes* | 28.85 | -111.76 |
| *C. cerastes* | 28.84 | -111.87 |
| *C. cerastes* | 36.75822 | -116.5734 |
| *C. cerastes* | 32.69366 | -115.3838 |
| *C. cerastes* | 31.84 | -113.29 |
| *C. cerastes* | 32.32 | -114.29 |
| *C. cerastes* | 31.88 | -113.55 |
| *C. cerastes* | 31.5 | -113.55 |
| *C. cerastes* | 31.57 | -113.38 |
| *C. cerastes* | 32.41 | -114.57 |
| *C. cerastes* | 31.8 | -112.86 |
| *C. cerastes* | 30.7 | -111.83 |
| *C. cerastes* | 29.09 | -111.11 |
| *C. cerastes* | 28.93 | -110.91 |
| *C. cerastes* | 29.09 | -111.06 |
| *C. cerastes* | 36.4333 | -114.55 |
| *C. cerastes* | 36.4333 | -114.483 |
| *C. cerastes* | 36.25 | -115.05 |
| *C. cerastes* | 35.65 | -114.9666 |
| *C. cerastes* | 36.7333 | -114.2166 |
| *C. cerastes* | 36.2166 | -116 |
| *C. cerastes* | 35.75 | -114.75 |
| *C. cerastes* | 36.45 | -114.5 |
| *C. cerastes* | 37.566 | -117.2 |
| *C. cerastes* | 35.2 | -114.566 |
| *C. cerastes* | 35.066 | -114.65 |
| *C. cerastes* | 35.05 | -114.6833 |
| *C. cerastes* | 35.7166 | -114.816 |
| *C. cerastes* | 35.866 | -115.1833 |
| *C. cerastes* | 36.733 | -116.633 |
| *C. cerastes* | 37 | -115.6 |
| *C. cerastes* | 35.012123 | -117.9159 |
| *C. cerastes* | 36.230324 | -115.9962 |
| *C. cerastes* | 36.71373 | -115.634 |
| *C. cerastes* | 36.419132 | -116.3131 |
| *C. cerastes* | 36.7334 | -116.7372 |
| *C. cerastes* | 36.5523 | -116.5266 |
| *C. cerastes* | 36.16612 | -116.8612 |
| *C. cerastes* | 36.60532 | -118.0616 |
| *C. cerastes* | 36.35158 | -117.5552 |
| *C. cerastes* | 34.83938 | -114.6091 |
| *C. cerastes* | 35.82527 | -115.7355 |
| *C. cerastes* | 32.17727 | -111.1406 |
| *C. cerastes* | 32.69806 | -114.6216 |
| *C. cerastes* | 32.93574 | -114.8606 |
| *C. cerastes* | 32.34455 | -112.8076 |
| *C. cerastes* | 33.49378 | -112.8784 |
| *C. cerastes* | 33.461483 | -112.4167 |
| *C. cerastes* | 35.8149 | -115.2315 |
| *C. cerastes* | 34.12283 | -114.2404 |
| *C. cerastes* | 34.0703 | -114.2597 |
| *C. cerastes* | 32.85452 | -114.3619 |
| *C. cerastes* | 32.25 | -111.2873 |
| *C. cerastes* | 33.605648 | -112.3139 |
| *C. cerastes* | 32.4471 | -112.8703 |
| *C. cerastes* | 32.33507 | -111.3212 |
| *C. cerastes* | 32.85036 | -116.2026 |
| *C. cerastes* | 32.91581 | -116.2416 |
| *C. cerastes* | 32.81247 | -116.1383 |
| *C. enyo* | 27.030903 | -112.0843 |
| *C. enyo* | 26.9 | -111.9833 |
| *C. enyo* | 28.6058 | -114.0496 |
| *C. enyo* | 30.07237 | -115.7331 |
| *C. enyo* | 26.05583 | -111.3675 |
| *C. enyo* | 28.90658 | -114.149 |
| *C. enyo* | 23.066668 | -109.7 |
| *C. enyo* | 26.994354 | -112.0649 |
| *C. enyo* | 25.8 | -111.334 |
| *C. enyo* | 27.95967 | -114.0564 |
| *C. enyo* | 30.07501 | -115.6384 |
| *C. enyo* | 30.13144 | -115.7589 |
| *C. enyo* | 30.34689 | -115.8183 |
| *C. enyo* | 30.09491 | -115.6647 |
| *C. enyo* | 30.06182 | -115.7277 |
| *C. enyo* | 24.39608 | -110.7241 |
| *C. enyo* | 23.80809 | -110.0536 |
| *C. enyo* | 28.17397 | -114.0026 |
| *C. enyo* | 30.08294 | -115.6907 |
| *C. enyo* | 29.03833 | -113.9113 |
| *C. enyo* | 30.08683 | -115.6861 |
| *C. enyo* | 24.17831 | -110.9407 |
| *C. enyo* | 27.28209 | -112.8958 |
| *C. enyo* | 27.383333 | -112.3167 |
| *C. enyo* | 23.4736 | -109.6937 |
| *C. enyo* | 28.58151 | -114.0491 |
| *C. enyo* | 28.96891 | -113.6415 |
| *C. enyo* | 24.142221 | -110.3108 |
| *C. enyo* | 24.56149 | -110.379 |
| *C. enyo* | 27.686111 | -113.7833 |
| *C. enyo* | 23.45 | -110.2167 |
| *C. enyo* | 23.6 | -109.6 |
| *C. enyo* | 30.7 | -116 |
| *C. enyo* | 27.281944 | -112.8958 |
| *C. enyo* | 24.454166 | -111.7667 |
| *C. enyo* | 28.981735 | -113.776 |
| *C. enyo* | 26.46312 | -111.6579 |
| *C. enyo* | 29.866667 | -114.95 |
| *C. enyo* | 27.338888 | -112.2669 |
| *C. enyo* | 27.833332 | -113.7 |
| *C. enyo* | 27.86 | -113.72 |
| *C. enyo* | 27.89 | -113.87 |
| *C. enyo* | 27.92 | -113.9 |
| *C. enyo* | 29.018888 | -113.8 |
| *C. enyo* | 23.768333 | -110.0558 |
| *C. enyo* | 26.17 | -111.44 |
| *C. enyo* | 29.701668 | -114.6844 |
| *C. enyo* | 29.65 | -114.6344 |
| *C. enyo* | 29.6 | -114.6172 |
| *C. enyo* | 29.401667 | -114.3844 |
| *C. enyo* | 29.4 | -114.3689 |
| *C. enyo* | 29.35 | -114.3189 |
| *C. enyo* | 29.284445 | -114.2672 |
| *C. enyo* | 29.28389 | -114.25 |
| *C. enyo* | 23.817778 | -110.2708 |
| *C. enyo* | 23.847221 | -110.2106 |
| *C. enyo* | 23.925 | -110.262 |
| *C. enyo* | 23.798332 | -110.2514 |
| *C. enyo* | 23.469444 | -110.2175 |
| *C. enyo* | 23.989445 | -110.1544 |
| *C. enyo* | 23.7975 | -110.2506 |
| *C. enyo* | 23.8125 | -110.0569 |
| *C. enyo* | 23.8425 | -110.2789 |
| *C. enyo* | 23.81361 | -110.2667 |
| *C. enyo* | 23.776388 | -110.2322 |
| *C. enyo* | 24.90889 | -112.2217 |
| *C. enyo* | 26.732779 | -112.1231 |
| *C. enyo* | 27.79 | -113.63 |
| *C. enyo* | 23.44861 | -110.2233 |
| *C. enyo* | 23.36694 | -109.7744 |
| *C. enyo* | 27.28361 | -112.8975 |
| *C. enyo* | 24.45 | -111.83 |
| *C. enyo* | 28.98 | -113.68 |
| *C. enyo* | 26.39 | -111.6 |
| *C. enyo* | 29.47 | -114.46 |
| *C. enyo* | 26.89 | -111.9783 |
| *C. enyo* | 27.82 | -113.69 |
| *C. enyo* | 27.82 | -113.7 |
| *C. enyo* | 27.89 | -113.86 |
| *C. enyo* | 27.89 | -113.91 |
| *C. enyo* | 29.15 | -113.71 |
| *C. enyo* | 23.77 | -110.06 |
| *C. enyo* | 29.71 | -114.68 |
| *C. enyo* | 29.67 | -114.63 |
| *C. enyo* | 29.62 | -114.6 |
| *C. enyo* | 29.4 | -114.4 |
| *C. enyo* | 29.38 | -114.38 |
| *C. enyo* | 29.32 | -114.31 |
| *C. enyo* | 29.3 | -114.23 |
| *C. enyo* | 29.27 | -114.22 |
| *C. enyo* | 23.93 | -110.11 |
| *C. enyo* | 23.96 | -110.11 |
| *C. enyo* | 23.92 | -110.26 |
| *C. enyo* | 23.29 | -109.78 |
| *C. enyo* | 23.9 | -110.11 |
| *C. enyo* | 23.41833 | -110.0183 |
| *C. enyo* | 23.98 | -110.26 |
| *C. enyo* | 23.8 | -110.06 |
| *C. enyo* | 23.94 | -110.11 |
| *C. enyo* | 23.92 | -110.11 |
| *C. enyo* | 23.87 | -110.1 |
| *C. enyo* | 24.58 | -112.07 |
| *C. enyo* | 25.07 | -112.17 |
| *C. enyo* | 26.98976 | -112.0628 |
| *C. enyo* | 27.18452 | -112.2002 |
| *C. enyo* | 27.67263 | -113.1964 |
| *C. enyo* | 27.415663 | -112.6126 |
| *C. enyo* | 28.98708 | -114.1561 |
| *C. enyo* | 28.96784 | -113.6617 |
| *C. enyo* | 23.2253 | -109.7324 |
| *C. enyo* | 29.04691 | -114.1359 |
| *C. enyo* | 22.88833 | -109.915 |
| *C. enyo* | 29.63167 | -114.6033 |
| *C. enyo* | 29.84018 | -114.8202 |
| *C. enyo* | 28.92498 | -113.5833 |
| *C. enyo* | 22.88821 | -109.9152 |
| *C. enyo* | 23.6 | -109.5833 |
| *C. enyo* | 24.82955 | -110.5738 |
| *C. enyo* | 25.95043 | -111.1674 |
| *C. enyo* | 23.30238 | -109.7662 |
| *C. enyo* | 28.96529 | -113.6933 |
| *C. enyo* | 30.08275 | -115.7374 |
| *C. enyo* | 28.95989 | -113.5587 |
| *C. enyo* | 28.9454 | -113.5591 |
| *C. enyo* | 24.88223 | -110.5283 |
| *C. enyo* | 29.96667 | -115.55 |
| *C. enyo* | 24.81667 | -110.7667 |
| *C. enyo* | 31.10025 | -116.1802 |
| *C. enyo* | 30.80019 | -116.0312 |
| *C. enyo* | 30.84167 | -116.0583 |
| *C. enyo* | 28.85018 | -114.128 |
| *C. enyo* | 28.92381 | -114.1601 |
| *C. enyo* | 24.22629 | -109.8727 |
| *C. enyo* | 23.58 | -110.21 |
| *C. enyo* | 23.501389 | -110.067 |
| *C. intermedius* | 17.551 | -96.721 |
| *C. intermedius* | 17.553 | -96.736 |
| *C. intermedius* | 17.836 | -97.569 |
| *C. intermedius* | 17.836 | -97.569 |
| *C. intermedius* | 17.606 | -96.764 |
| *C. intermedius* | 18.67 | -97.469 |
| *C. intermedius* | 18.67 | -97.469 |
| *C. intermedius* | 18.769 | -97.536 |
| *C. intermedius* | 19.599 | -97.099 |
| *C. intermedius* | 17.542 | -99.692 |
| *C. intermedius* | 17.558 | -99.675 |
| *C. intermedius* | 17.558 | -99.692 |
| *C. intermedius* | 19.506 | -97.342 |
| *C. intermedius* | 19.613 | -97.042 |
| *C. intermedius* | 19.478 | -97.372 |
| *C. intermedius* | 19.624 | -97.069 |
| *C. intermedius* | 19.637 | -97.1 |
| *C. intermedius* | 17.23 | -97 |
| *C. intermedius* | 20.14 | -98.67 |
| *C. intermedius* | 17.330555 | -96.48722 |
| *C. intermedius* | 17.163889 | -96.58083 |
| *C. intermedius* | 17.55139 | -99.50083 |
| *C. intermedius* | 17.536 | -96.735 |
| *C. intermedius* | 17.838 | -96.801 |
| *C. intermedius* | 17.17 | -97.64 |
| *C. intermedius* | 17.6 | -99.73 |
| *C. intermedius* | 17.56 | -99.66 |
| *C. intermedius* | 17.55 | -99.68 |
| *C. intermedius* | 17.55667 | -99.68556 |
| *C. intermedius* | 19.65 | -97.08 |
| *C. intermedius* | 17.53111 | -96.8275 |
| *C. intermedius* | 17.79 | -96.92 |
| *C. intermedius* | 19.595 | -97.1 |
| *C. intermedius* | 18.465 | -97.562 |
| *C. intermedius* | 17.553 | -96.751 |
| *C. intermedius* | 18.3 | -97.5 |
| *C. intermedius* | 19.4 | -96.96667 |
| *C. intermedius* | 18.553 | -97.586 |
| *C. intermedius* | 17.19 | -96.58 |
| *C. intermedius* | 17.44 | -99.57 |
| *C. intermedius* | 19.5 | -97.333 |
| *C. lepidus* | 32.47186 | -106.7778 |
| *C. lepidus* | 23.93 | -105.63 |
| *C. lepidus* | 30.53243 | -103.8071 |
| *C. lepidus* | 30.60156 | -103.9122 |
| *C. lepidus* | 23.82 | -104.93 |
| *C. lepidus* | 23.55 | -103.65 |
| *C. lepidus* | 29.237803 | -103.0403 |
| *C. lepidus* | 22.82 | -103.733 |
| *C. lepidus* | 22.3 | -102.498 |
| *C. lepidus* | 22.226 | -102.185 |
| *C. lepidus* | 26.736 | -103.836 |
| *C. lepidus* | 23.411 | -104.205 |
| *C. lepidus* | 24.876 | -100.22 |
| *C. lepidus* | 30.569344 | -104.6327 |
| *C. lepidus* | 30.66295 | -104.0269 |
| *C. lepidus* | 31.84545 | -106.4777 |
| *C. lepidus* | 29.72137 | -99.67412 |
| *C. lepidus* | 32.512897 | -106.8202 |
| *C. lepidus* | 32.755756 | -106.5826 |
| *C. lepidus* | 31.724789 | -110.88 |
| *C. lepidus* | 23.39 | -105.81 |
| *C. lepidus* | 32.86333 | -108.2208 |
| *C. lepidus* | 30.81413 | -104.0196 |
| *C. lepidus* | 25.86 | -100.53 |
| *C. lepidus* | 30.97 | -108.14 |
| *C. lepidus* | 25.46 | -104.6 |
| *C. lepidus* | 25.809 | -100.593 |
| *C. lepidus* | 31.291666 | -107.3 |
| *C. lepidus* | 31.435 | -110.4268 |
| *C. lepidus* | 31.538979 | -108.7096 |
| *C. lepidus* | 24.827 | -100.076 |
| *C. lepidus* | 23.972 | -99.77 |
| *C. lepidus* | 32.92 | -106.64 |
| *C. lepidus* | 33.38 | -108.46 |
| *C. lepidus* | 33.6 | -107.4 |
| *C. lepidus* | 34.02 | -107.13 |
| *C. lepidus* | 33.81 | -107.41 |
| *C. lepidus* | 32.18 | -104.47 |
| *C. lepidus* | 31.54 | -108.66 |
| *C. lepidus* | 34.1 | -107.3 |
| *C. lepidus* | 31.34 | -108.98 |
| *C. lepidus* | 33.5 | -107.4 |
| *C. lepidus* | 31.57 | -108.32 |
| *C. lepidus* | 34.05 | -107.19 |
| *C. lepidus* | 32.18 | -104.38 |
| *C. lepidus* | 31.57 | -108.79 |
| *C. lepidus* | 33.16 | -106.55 |
| *C. lepidus* | 31.52 | -109.04 |
| *C. lepidus* | 32.34 | -106.57 |
| *C. lepidus* | 32.57 | -106.51 |
| *C. lepidus* | 31.34 | -108.74 |
| *C. lepidus* | 34.03 | -107.13 |
| *C. lepidus* | 33.2 | -106.5 |
| *C. lepidus* | 32.74 | -108.06 |
| *C. lepidus* | 32.22 | -104.63 |
| *C. lepidus* | 32.26 | -104.72 |
| *C. lepidus* | 33.53 | -106.49 |
| *C. lepidus* | 32.18 | -104.44 |
| *C. lepidus* | 32.89 | -107.84 |
| *C. lepidus* | 33.04 | -107.56 |
| *C. lepidus* | 34.15 | -106.99 |
| *C. lepidus* | 34.14 | -106.99 |
| *C. lepidus* | 31.59 | -108.77 |
| *C. lepidus* | 32.91 | -108.22 |
| *C. lepidus* | 33.08 | -107.69 |
| *C. lepidus* | 32.54 | -107.21 |
| *C. lepidus* | 33.87 | -107.12 |
| *C. lepidus* | 33.58 | -107.4 |
| *C. lepidus* | 34.1 | -107.26 |
| *C. lepidus* | 33.5 | -107.42 |
| *C. lepidus* | 31.57 | -108.78 |
| *C. lepidus* | 31.34 | -108.78 |
| *C. lepidus* | 33.23 | -106.5 |
| *C. lepidus* | 31.61 | -108.78 |
| *C. lepidus* | 29.93373 | -101.173 |
| *C. lepidus* | 32.0074 | -109.3227 |
| *C. lepidus* | 31.8793 | -109.3481 |
| *C. lepidus* | 31.8961 | -109.1631 |
| *C. lepidus* | 31.839 | -106.643 |
| *C. lepidus* | 31.997 | -106.449 |
| *C. lepidus* | 31.4162 | -110.2714 |
| *C. lepidus* | 30.014645 | -108.4168 |
| *C. lepidus* | 31.9297 | -109.3817 |
| *C. lepidus* | 28.977964 | -102.5518 |
| *C. lepidus* | 29.06028 | -102.5422 |
| *C. lepidus* | 23.630964 | -105.8415 |
| *C. lepidus* | 31.8722 | -109.2154 |
| *C. lepidus* | 31.8748 | -109.2124 |
| *C. lepidus* | 31.748518 | -108.9859 |
| *C. lepidus* | 31.727777 | -108.9128 |
| *C. lepidus* | 31.754107 | -108.9356 |
| *C. lepidus* | 31.8427 | -109.2118 |
| *C. lepidus* | 30.08899 | -100.896 |
| *C. lepidus* | 29.44306 | -106.8167 |
| *C. lepidus* | 29.392097 | -106.8987 |
| *C. lepidus* | 29.3226 | -106.45 |
| *C. lepidus* | 31.8898 | -109.2033 |
| *C. lepidus* | 29.433773 | -106.871 |
| *C. lepidus* | 29.266 | -103.301 |
| *C. lepidus* | 30.03 | -108.43 |
| *C. lepidus* | 25.58 | -101.97 |
| *C. lepidus* | 25.66 | -101.96 |
| *C. lepidus* | 29.44 | -107.05 |
| *C. lepidus* | 29.45 | -106.87 |
| *C. lepidus* | 29.35 | -106.36 |
| *C. lepidus* | 21.84 | -102.88 |
| *C. lepidus* | 31.51 | -110.36 |
| *C. lepidus* | 28.953333 | -107.825 |
| *C. lepidus* | 30.7233 | -103.93 |
| *C. lepidus* | 32.99582 | -108.2814 |
| *C. lepidus* | 32.91416 | -107.9792 |
| *C. lepidus* | 29.85 | -108.72 |
| *C. lepidus* | 29.76897 | -99.89088 |
| *C. lepidus* | 23.64 | -105.84 |
| *C. lepidus* | 31.7909 | -106.3947 |
| *C. lepidus* | 32.91618 | -107.5999 |
| *C. lepidus* | 31.71313 | -110.8798 |
| *C. lepidus* | 23.58 | -106.03 |
| *C. lepidus* | 23.55 | -105.98 |
| *C. lepidus* | 23.57 | -105.87 |
| *C. lepidus* | 22.65 | -105.07 |
| *C. lepidus* | 22.44472 | -104.7972 |
| *C. lepidus* | 27.31 | -102.46 |
| *C. lepidus* | 24.86 | -99.28 |
| *C. lepidus* | 23.048 | -99.221 |
| *C. lepidus* | 23.15 | -99.31 |
| *C. lepidus* | 24.592 | -99.995 |
| *C. lepidus* | 25.604 | -100.398 |
| *C. lepidus* | 23.908 | -99.797 |
| *C. lepidus* | 25.578 | -100.306 |
| *C. lepidus* | 24.846 | -100.32 |
| *C. lepidus* | 25.587 | -100.369 |
| *C. lepidus* | 25.298 | -100.162 |
| *C. lepidus* | 25.35 | -100.16 |
| *C. lepidus* | 25.643 | -100.352 |
| *C. lepidus* | 25.691 | -100.168 |
| *C. lepidus* | 23.601 | -99.706 |
| *C. lepidus* | 23.553 | -99.6 |
| *C. lepidus* | 23.646 | -99.596 |
| *C. lepidus* | 23.47 | -99.596 |
| *C. lepidus* | 25.65 | -100.458 |
| *C. lepidus* | 23.72 | -105.86 |
| *C. lepidus* | 27.02 | -104.33 |
| *C. lepidus* | 28.10361 | -108.1108 |
| *C. lepidus* | 24.73 | -103.88 |
| *C. lepidus* | 24.61194 | -100.1478 |
| *C. lepidus* | 23.186 | -99.174 |
| *C. lepidus* | 23.15 | -99.04 |
| *C. lepidus* | 23.106 | -99.171 |
| *C. lepidus* | 23.04 | -99.23 |
| *C. lepidus* | 23.176 | -99.31 |
| *C. lepidus* | 23.19 | -99.3 |
| *C. lepidus* | 23.11 | -99.164 |
| *C. lepidus* | 23.07 | -99.27 |
| *C. lepidus* | 23.088 | -99.166 |
| *C. lepidus* | 23.12 | -99.21 |
| *C. lepidus* | 23.077 | -98.949 |
| *C. lepidus* | 23.11 | -99.23 |
| *C. lepidus* | 23.113 | -99.169 |
| *C. lepidus* | 24.003 | -98.894 |
| *C. lepidus* | 23.95 | -99.39 |
| *C. lepidus* | 27.85806 | -107.9939 |
| *C. lepidus* | 23.106 | -99.199 |
| *C. lepidus* | 23.13 | -101.1156 |
| *C. lepidus* | 31.67569 | -105.9168 |
| *C. lepidus* | 23.001 | -99.153 |
| *C. lepidus* | 25.71 | -100.373 |
| *C. lepidus* | 24.704 | -100.03 |
| *C. lepidus* | 22.25 | -99.12 |
| *C. lepidus* | 22.41 | -101.43 |
| *C. lepidus* | 29.33941 | -104.0617 |
| *C. lepidus* | 32.6276 | -107.8672 |
| *C. lepidus* | 32.46224 | -106.803 |
| *C. lepidus* | 31.8943 | -106.4729 |
| *C. lepidus* | 31.89423 | -106.4696 |
| *C. lepidus* | 30.78042 | -104.9946 |
| *C. lepidus* | 31.960293 | -108.1603 |
| *C. lepidus* | 30.7768 | -105.0162 |
| *C. lepidus* | 31.89791 | -106.4902 |
| *C. lepidus* | 32.90428 | -108.2361 |
| *C. lepidus* | 30.32045 | -100.7376 |
| *C. lepidus* | 31.47352 | -108.4349 |
| *C. lepidus* | 30.54055 | -103.8376 |
| *C. lepidus* | 32.203815 | -104.4904 |
| *C. lepidus* | 31.68958 | -104.7746 |
| *C. lepidus* | 31.87962 | -106.4989 |
| *C. lepidus* | 31.5421 | -110.5107 |
| *C. lepidus* | 31.8904 | -106.4894 |
| *C. lepidus* | 30.79737 | -105.0105 |
| *C. lepidus* | 29.26815 | -103.7584 |
| *C. lepidus* | 32.118233 | -107.6385 |
| *C. lepidus* | 31.89345 | -106.4701 |
| *C. lepidus* | 31.96093 | -108.1603 |
| *C. lepidus* | 32.48519 | -107.1241 |
| *C. lepidus* | 31.88095 | -106.5017 |
| *C. lepidus* | 31.91856 | -106.0437 |
| *C. lepidus* | 31.88183 | -106.4905 |
| *C. lepidus* | 32.49308 | -107.1393 |
| *C. lepidus* | 30.29283 | -100.8775 |
| *C. lepidus* | 31.86435 | -106.4905 |
| *C. lepidus* | 31.88361 | -106.5034 |
| *C. lepidus* | 29.79143 | -104.276 |
| *C. lepidus* | 30.93812 | -105.0487 |
| *C. lepidus* | 31.8457 | -106.5094 |
| *C. lepidus* | 32.36937 | -106.5648 |
| *C. lepidus* | 32.75002 | -107.8934 |
| *C. lepidus* | 30.67287 | -100.8606 |
| *C. lepidus* | 31.92946 | -108.5252 |
| *C. lepidus* | 31.83489 | -106.4779 |
| *C. lepidus* | 31.90286 | -106.4931 |
| *C. lepidus* | 30.5497 | -104.0969 |
| *C. lepidus* | 31.68794 | -105.9677 |
| *C. lepidus* | 31.8857 | -106.484 |
| *C. lepidus* | 29.00428 | -107.8523 |
| *C. lepidus* | 31.91753 | -104.8891 |
| *C. lepidus* | 29.32039 | -103.6148 |
| *C. lepidus* | 29.54 | -106.78 |
| *C. lepidus* | 30.7348 | -103.7775 |
| *C. lepidus* | 23.81 | -105.16 |
| *C. lepidus* | 22.86 | -103.73 |
| *C. lepidus* | 21.71 | -103.07 |
| *C. lepidus* | 22.41 | -101.42 |
| *C. lepidus* | 24.7 | -101.25 |
| *C. lepidus* | 23.79 | -105.14 |
| *C. lepidus* | 23.81 | -105.71 |
| *C. lepidus* | 31.71438 | -105.9974 |
| *C. lepidus* | 22.41 | -102.44 |
| *C. lepidus* | 29.01 | -107.85 |
| *C. lepidus* | 28.76 | -107.26 |
| *C. lepidus* | 23.81 | -105.13 |
| *C. lepidus* | 22.92 | -103.77 |
| *C. lepidus* | 21.65 | -103.12 |
| *C. lepidus* | 24.71 | -101.25 |
| *C. lepidus* | 23.75 | -105.18 |
| *C. lepidus* | 23.88 | -105.89 |
| *C. lepidus* | 33.35 | -108.39 |
| *C. lepidus* | 31.55 | -108.29 |
| *C. lepidus* | 32.888885 | -108.2281 |
| *C. lepidus* | 30.84549 | -103.8223 |
| *C. molossus* | 17.65 | -97.134 |
| *C. molossus* | 23.75 | -105.5333 |
| *C. molossus* | 19.66 | -100.8 |
| *C. molossus* | 23.6 | -99.71 |
| *C. molossus* | 29.27851 | -103.8369 |
| *C. molossus* | 25.44 | -104.6 |
| *C. molossus* | 27.43333 | -107.5667 |
| *C. molossus* | 33.83334 | -111.9507 |
| *C. molossus* | 23.82 | -104.93 |
| *C. molossus* | 30.625 | -103.8648 |
| *C. molossus* | 31.539494 | -110.7562 |
| *C. molossus* | 31.616268 | -110.8083 |
| *C. molossus* | 19.46667 | -101.6 |
| *C. molossus* | 31.365377 | -110.774 |
| *C. molossus* | 30.61959 | -104.0014 |
| *C. molossus* | 20.131 | -98.527 |
| *C. molossus* | 19.973 | -98.862 |
| *C. molossus* | 31.8831 | -109.2053 |
| *C. molossus* | 29.237803 | -103.0403 |
| *C. molossus* | 31.9 | -109.1 |
| *C. molossus* | 17.607 | -96.783 |
| *C. molossus* | 18.465 | -97.562 |
| *C. molossus* | 18.333 | -97.453 |
| *C. molossus* | 17.735 | -96.967 |
| *C. molossus* | 18.219 | -97.333 |
| *C. molossus* | 22.137 | -100.837 |
| *C. molossus* | 21.929 | -102.093 |
| *C. molossus* | 20.767 | -99.399 |
| *C. molossus* | 19.57 | -97.491 |
| *C. molossus* | 19.268 | -99.207 |
| *C. molossus* | 19.003 | -99.204 |
| *C. molossus* | 19.292 | -99.172 |
| *C. molossus* | 22.279 | -100.849 |
| *C. molossus* | 19.062 | -97.873 |
| *C. molossus* | 25.095 | -106 |
| *C. molossus* | 23.419 | -104.261 |
| *C. molossus* | 23.433 | -104.25 |
| *C. molossus* | 23.418 | -104.24 |
| *C. molossus* | 19.5 | -97.333 |
| *C. molossus* | 25.543 | -99.842 |
| *C. molossus* | 24.876 | -100.22 |
| *C. molossus* | 32.747498 | -105.9147 |
| *C. molossus* | 31.67196 | -110.9424 |
| *C. molossus* | 23.66725 | -100.8809 |
| *C. molossus* | 29.272167 | -103.2983 |
| *C. molossus* | 31.4538 | -110.3762 |
| *C. molossus* | 29.317743 | -103.6538 |
| *C. molossus* | 30.549955 | -104.6612 |
| *C. molossus* | 33.704678 | -111.5108 |
| *C. molossus* | 31.721085 | -110.8798 |
| *C. molossus* | 34.202522 | -112.8599 |
| *C. molossus* | 33.785465 | -108.7689 |
| *C. molossus* | 32.194683 | -104.3401 |
| *C. molossus* | 33.733 | -105.9245 |
| *C. molossus* | 29.54857 | -102.935 |
| *C. molossus* | 29.20063 | -102.9919 |
| *C. molossus* | 29.32824 | -103.2053 |
| *C. molossus* | 29.27406 | -103.2854 |
| *C. molossus* | 19.69 | -101.6 |
| *C. molossus* | 28.64 | -111.01 |
| *C. molossus* | 30.97 | -112.36 |
| *C. molossus* | 27.19 | -109.55 |
| *C. molossus* | 27.58 | -109.4 |
| *C. molossus* | 28.02 | -111.18 |
| *C. molossus* | 23.91 | -105.41 |
| *C. molossus* | 25.66 | -105.01 |
| *C. molossus* | 30.34 | -109.52 |
| *C. molossus* | 32.002148 | -106.4907 |
| *C. molossus* | 29.27233 | -103.8329 |
| *C. molossus* | 31.82694 | -105.9375 |
| *C. molossus* | 26.92 | -102.14 |
| *C. molossus* | 33.115818 | -109.0052 |
| *C. molossus* | 29.74823 | -99.5912 |
| *C. molossus* | 30.04763 | -103.5756 |
| *C. molossus* | 30.36 | -109.65 |
| *C. molossus* | 30.15122 | -102.5351 |
| *C. molossus* | 30.96 | -112.37 |
| *C. molossus* | 32.3365 | -110.9103 |
| *C. molossus* | 33.646187 | -106.3715 |
| *C. molossus* | 33.782894 | -106.4441 |
| *C. molossus* | 33.542187 | -106.4402 |
| *C. molossus* | 33.141132 | -107.5382 |
| *C. molossus* | 28.7 | -110.99 |
| *C. molossus* | 30.59353 | -103.8895 |
| *C. molossus* | 31.82715 | -105.8922 |
| *C. molossus* | 31.72139 | -108.4219 |
| *C. molossus* | 31.651848 | -108.4036 |
| *C. molossus* | 32.19806 | -106.6 |
| *C. molossus* | 32.10675 | -107.0206 |
| *C. molossus* | 33.15711 | -107.1494 |
| *C. molossus* | 31.713129 | -110.8798 |
| *C. molossus* | 23.65 | -105.68 |
| *C. molossus* | 32.899384 | -108.2467 |
| *C. molossus* | 30.81413 | -104.0196 |
| *C. molossus* | 30.44138 | -103.7069 |
| *C. molossus* | 30.08333 | -108.35 |
| *C. molossus* | 23.51111 | -100.6206 |
| *C. molossus* | 27.66667 | -108.7167 |
| *C. molossus* | 22.03778 | -100.6381 |
| *C. molossus* | 31.291666 | -107.3 |
| *C. molossus* | 31.57226 | -109.3333 |
| *C. molossus* | 31.6792 | -110.8797 |
| *C. molossus* | 31.2333 | -111.0298 |
| *C. molossus* | 32.62581 | -107.0366 |
| *C. molossus* | 33.316666 | -108.9172 |
| *C. molossus* | 32.75 | -105.92 |
| *C. molossus* | 35.12 | -106.54 |
| *C. molossus* | 35.47 | -107.12 |
| *C. molossus* | 35.21 | -106.51 |
| *C. molossus* | 33.54 | -105.49 |
| *C. molossus* | 35.2 | -106.4 |
| *C. molossus* | 32.18 | -104.44 |
| *C. molossus* | 33.6 | -107.4 |
| *C. molossus* | 34.02 | -107.13 |
| *C. molossus* | 32.88 | -107.55 |
| *C. molossus* | 34.33 | -107.09 |
| *C. molossus* | 33.78 | -107.32 |
| *C. molossus* | 35.02 | -106.35 |
| *C. molossus* | 33.71 | -105.93 |
| *C. molossus* | 32.36 | -104.42 |
| *C. molossus* | 32.9 | -107.8 |
| *C. molossus* | 31.5 | -108.7 |
| *C. molossus* | 34.02 | -107.15 |
| *C. molossus* | 33.68 | -105.57 |
| *C. molossus* | 33.2 | -108.8 |
| *C. molossus* | 32.69 | -107.84 |
| *C. molossus* | 33.2 | -108.9 |
| *C. molossus* | 31.94 | -108.95 |
| *C. molossus* | 32.73 | -107.57 |
| *C. molossus* | 32.36 | -109.02 |
| *C. molossus* | 32.47 | -104.44 |
| *C. molossus* | 32.3 | -104.7 |
| *C. molossus* | 33.22 | -107.32 |
| *C. molossus* | 32.96 | -105.92 |
| *C. molossus* | 32.6 | -106.5 |
| *C. molossus* | 32.78 | -106.59 |
| *C. molossus* | 32.92 | -107.66 |
| *C. molossus* | 33.72 | -108.78 |
| *C. molossus* | 33.27 | -108.87 |
| *C. molossus* | 31.34 | -108.78 |
| *C. molossus* | 34.31 | -106.64 |
| *C. molossus* | 32.26 | -104.72 |
| *C. molossus* | 32.26 | -104.68 |
| *C. molossus* | 32.21 | -104.63 |
| *C. molossus* | 32.2 | -104.6 |
| *C. molossus* | 33.06 | -107.54 |
| *C. molossus* | 32.46 | -106.11 |
| *C. molossus* | 33.81 | -106.23 |
| *C. molossus* | 32.74 | -106.46 |
| *C. molossus* | 32.92 | -107.57 |
| *C. molossus* | 32.92 | -107.69 |
| *C. molossus* | 32.8 | -107.9 |
| *C. molossus* | 31.77 | -108.98 |
| *C. molossus* | 31.73 | -108.98 |
| *C. molossus* | 34.14 | -106.99 |
| *C. molossus* | 33.47 | -107.25 |
| *C. molossus* | 33.2 | -107.2 |
| *C. molossus* | 34.34 | -107.28 |
| *C. molossus* | 33.19 | -107.54 |
| *C. molossus* | 32.94 | -107.51 |
| *C. molossus* | 33.02 | -108.72 |
| *C. molossus* | 33.44 | -107.43 |
| *C. molossus* | 34.06 | -107.08 |
| *C. molossus* | 32.71 | -108.71 |
| *C. molossus* | 35.08 | -106.65 |
| *C. molossus* | 34.42 | -106.97 |
| *C. molossus* | 32.62 | -108.41 |
| *C. molossus* | 35.2 | -106.43 |
| *C. molossus* | 35.29 | -106.43 |
| *C. molossus* | 31.59 | -108.77 |
| *C. molossus* | 33.35 | -108.08 |
| *C. molossus* | 35.13 | -106.49 |
| *C. molossus* | 33.58 | -107.4 |
| *C. molossus* | 33.81 | -106.24 |
| *C. molossus* | 31.57 | -108.32 |
| *C. molossus* | 32.91 | -107.8 |
| *C. molossus* | 31.5 | -108.71 |
| *C. molossus* | 31.34 | -108.71 |
| *C. molossus* | 35.22 | -106.48 |
| *C. molossus* | 31.49 | -108.79 |
| *C. molossus* | 33.2 | -108.83 |
| *C. molossus* | 32.77 | -108.62 |
| *C. molossus* | 33.2 | -108.86 |
| *C. molossus* | 32.85 | -108.59 |
| *C. molossus* | 33.08 | -108.49 |
| *C. molossus* | 32.26 | -104.7 |
| *C. molossus* | 35.07 | -106.49 |
| *C. molossus* | 32.56 | -106.5 |
| *C. molossus* | 32.85 | -107.88 |
| *C. molossus* | 30.61 | -104 |
| *C. molossus* | 34.56 | -106.43 |
| *C. molossus* | 32.2 | -104.63 |
| *C. molossus* | 32.87 | -107.98 |
| *C. molossus* | 31.43 | -108.35 |
| *C. molossus* | 35.14 | -106.56 |
| *C. molossus* | 33.77 | -108.68 |
| *C. molossus* | 29.16266 | -110.9097 |
| *C. molossus* | 33.325035 | -106.3195 |
| *C. molossus* | 31.4316 | -109.894 |
| *C. molossus* | 31.8996 | -109.1605 |
| *C. molossus* | 32.0031 | -109.3253 |
| *C. molossus* | 31.8901 | -109.1686 |
| *C. molossus* | 31.9572 | -109.1408 |
| *C. molossus* | 31.4899 | -109.4311 |
| *C. molossus* | 31.8861 | -109.1708 |
| *C. molossus* | 31.9164 | -109.2643 |
| *C. molossus* | 31.9077 | -109.1088 |
| *C. molossus* | 31.9064 | -109.1531 |
| *C. molossus* | 31.9332 | -109.1794 |
| *C. molossus* | 31.8817 | -109.1822 |
| *C. molossus* | 31.9148 | -109.2683 |
| *C. molossus* | 31.9136 | -109.1408 |
| *C. molossus* | 31.858793 | -109.0326 |
| *C. molossus* | 31.89 | -109.1132 |
| *C. molossus* | 31.937 | -109.2169 |
| *C. molossus* | 32.082294 | -108.9773 |
| *C. molossus* | 31.8809 | -109.1948 |
| *C. molossus* | 31.7099 | -110.7595 |
| *C. molossus* | 31.9308 | -109.1918 |
| *C. molossus* | 31.8756 | -109.1853 |
| *C. molossus* | 31.918 | -109.1565 |
| *C. molossus* | 31.8767 | -109.2219 |
| *C. molossus* | 31.9335 | -109.181 |
| *C. molossus* | 31.927 | -109.1688 |
| *C. molossus* | 31.9311 | -109.1752 |
| *C. molossus* | 31.8825 | -109.2033 |
| *C. molossus* | 31.4657 | -109.3834 |
| *C. molossus* | 31.8833 | -109.1742 |
| *C. molossus* | 32.08861 | -108.9731 |
| *C. molossus* | 31.9316 | -109.1767 |
| *C. molossus* | 31.8726 | -109.189 |
| *C. molossus* | 31.9426 | -109.1408 |
| *C. molossus* | 31.4956 | -109.3328 |
| *C. molossus* | 32.101353 | -109.0301 |
| *C. molossus* | 27.45694 | -105.4 |
| *C. molossus* | 29.255 | -103.216 |
| *C. molossus* | 31.08323 | -112.4142 |
| *C. molossus* | 32.847446 | -108.3243 |
| *C. molossus* | 31.4567 | -110.2389 |
| *C. molossus* | 30.014645 | -108.4168 |
| *C. molossus* | 31.3886 | -111.1255 |
| *C. molossus* | 29.06028 | -102.5422 |
| *C. molossus* | 31.8992 | -109.1611 |
| *C. molossus* | 31.8732 | -109.1846 |
| *C. molossus* | 31.8825 | -109.1863 |
| *C. molossus* | 29.44306 | -106.8167 |
| *C. molossus* | 29.485098 | -106.7704 |
| *C. molossus* | 29.494436 | -106.7871 |
| *C. molossus* | 29.392097 | -106.8987 |
| *C. molossus* | 29.3226 | -106.45 |
| *C. molossus* | 22.285 | -102.1 |
| *C. molossus* | 31.8998 | -109.2592 |
| *C. molossus* | 33.6279 | -111.5584 |
| *C. molossus* | 32.2222 | -111.1003 |
| *C. molossus* | 31.9297 | -109.3817 |
| *C. molossus* | 31.9588 | -109.3137 |
| *C. molossus* | 29.23 | -110.95 |
| *C. molossus* | 27.34 | -105.47 |
| *C. molossus* | 31.08 | -112.48 |
| *C. molossus* | 30.03 | -108.43 |
| *C. molossus* | 25.66 | -101.96 |
| *C. molossus* | 26.42 | -106.48 |
| *C. molossus* | 29.44 | -107.05 |
| *C. molossus* | 29.32 | -107.08 |
| *C. molossus* | 29.45 | -106.87 |
| *C. molossus* | 29.35 | -106.36 |
| *C. molossus* | 20.914 | -99.769 |
| *C. molossus* | 20.681 | -99.556 |
| *C. molossus* | 20.656 | -99.586 |
| *C. molossus* | 31.24 | -106.28 |
| *C. molossus* | 21.05 | -101.33 |
| *C. molossus* | 19.4 | -99.2 |
| *C. molossus* | 19.23 | -99.22 |
| *C. molossus* | 19.24333 | -99.23722 |
| *C. molossus* | 19.35 | -99.16167 |
| *C. molossus* | 20.6825 | -99.5625 |
| *C. molossus* | 20.71 | -99.61 |
| *C. molossus* | 20.91556 | -99.76694 |
| *C. molossus* | 18.317 | -97.45 |
| *C. molossus* | 18.363 | -97.486 |
| *C. molossus* | 18.317 | -97.55 |
| *C. molossus* | 24.248 | -100.052 |
| *C. molossus* | 25.973 | -100.566 |
| *C. molossus* | 27.02 | -100.84 |
| *C. molossus* | 24.08 | -101.41 |
| *C. molossus* | 27.020296 | -100.8303 |
| *C. molossus* | 24.110336 | -101.3803 |
| *C. molossus* | 32.632504 | -106.7776 |
| *C. molossus* | 32.425274 | -106.5667 |
| *C. molossus* | 32.380745 | -106.4791 |
| *C. molossus* | 31.32 | -108.71 |
| *C. molossus* | 25.18 | -103.72 |
| *C. molossus* | 30.97469 | -110.0194 |
| *C. molossus* | 18.3 | -97.5 |
| *C. molossus* | 32.71913 | -109.8798 |
| *C. molossus* | 31.723333 | -110.8803 |
| *C. molossus* | 34.98141 | -112.3768 |
| *C. molossus* | 34.60697 | -112.4041 |
| *C. molossus* | 34.54002 | -112.4685 |
| *C. molossus* | 34.39783 | -112.2363 |
| *C. molossus* | 23.8 | -102.4667 |
| *C. molossus* | 32.9285 | -107.9809 |
| *C. molossus* | 33.07111 | -108.365 |
| *C. molossus* | 32.91861 | -108.0314 |
| *C. molossus* | 32.91416 | -107.9792 |
| *C. molossus* | 32.92834 | -107.9809 |
| *C. molossus* | 35.03665 | -107.3823 |
| *C. molossus* | 28.05 | -110.95 |
| *C. molossus* | 34.16253 | -112.8507 |
| *C. molossus* | 34.31728 | -112.9224 |
| *C. molossus* | 27.98333 | -110.9667 |
| *C. molossus* | 34.41836 | -112.9171 |
| *C. molossus* | 33.64639 | -106.3714 |
| *C. molossus* | 24.32 | -105.83 |
| *C. molossus* | 31.7909 | -106.3947 |
| *C. molossus* | 19.04333 | -98.19667 |
| *C. molossus* | 31.54344 | -110.7826 |
| *C. molossus* | 30.42534 | -103.6972 |
| *C. molossus* | 24.81 | -103.82 |
| *C. molossus* | 34.16283 | -112.8449 |
| *C. molossus* | 26.34 | -101.36 |
| *C. molossus* | 30.6059 | -103.9743 |
| *C. molossus* | 36.27368 | -112.3353 |
| *C. molossus* | 31.93629 | -108.9371 |
| *C. molossus* | 23.85 | -105.31 |
| *C. molossus* | 27.3 | -107.5417 |
| *C. molossus* | 29.28836 | -103.2894 |
| *C. molossus* | 18 | -97.77 |
| *C. molossus* | 25.25 | -100.77 |
| *C. molossus* | 21.295 | -99.634 |
| *C. molossus* | 21.14 | -99.52 |
| *C. molossus* | 25.69 | -100.99 |
| *C. molossus* | 26.76 | -105.86 |
| *C. molossus* | 23.905 | -99.794 |
| *C. molossus* | 25.372 | -100.221 |
| *C. molossus* | 25.35 | -100.16 |
| *C. molossus* | 24.744 | -99.941 |
| *C. molossus* | 24.019 | -99.966 |
| *C. molossus* | 25.3 | -100.141 |
| *C. molossus* | 25.49 | -101 |
| *C. molossus* | 28.01 | -111.03 |
| *C. molossus* | 24.78 | -103.8 |
| *C. molossus* | 23.38306 | -103.7561 |
| *C. molossus* | 23.39 | -103.82 |
| *C. molossus* | 20.67 | -99.19 |
| *C. molossus* | 18.6425 | -97.39806 |
| *C. molossus* | 23.43 | -103.19 |
| *C. molossus* | 18.85 | -97.103 |
| *C. molossus* | 21.136 | -99.625 |
| *C. molossus* | 20.738 | -99.942 |
| *C. molossus* | 20.694 | -99.815 |
| *C. molossus* | 24.07 | -105.55 |
| *C. molossus* | 23.99 | -105.14 |
| *C. molossus* | 24.09556 | -105.5492 |
| *C. molossus* | 21.13417 | -99.62528 |
| *C. molossus* | 23.17056 | -99.29917 |
| *C. molossus* | 23.176 | -99.31 |
| *C. molossus* | 21.21 | -99.19 |
| *C. molossus* | 27.85806 | -107.9939 |
| *C. molossus* | 28.10361 | -108.1108 |
| *C. molossus* | 20.75 | -99.96 |
| *C. molossus* | 29 | -110.85 |
| *C. molossus* | 21.46 | -101.21 |
| *C. molossus* | 23.15028 | -101.2286 |
| *C. molossus* | 23.13 | -101.1156 |
| *C. molossus* | 30.04 | -110.8 |
| *C. molossus* | 19.41 | -102.12 |
| *C. molossus* | 20.69333 | -99.81333 |
| *C. molossus* | 32.083 | -113.25 |
| *C. molossus* | 26.101 | -99.824 |
| *C. molossus* | 24.676 | -100.3 |
| *C. molossus* | 18.312 | -97.51 |
| *C. molossus* | 24.827 | -100.076 |
| *C. molossus* | 24.327 | -99.912 |
| *C. molossus* | 18.285 | -97.486 |
| *C. molossus* | 18.32 | -97.518 |
| *C. molossus* | 30.79063 | -105.0185 |
| *C. molossus* | 29.35888 | -104.0875 |
| *C. molossus* | 31.14871 | -104.817 |
| *C. molossus* | 32.898144 | -107.2693 |
| *C. molossus* | 32.9168 | -107.7098 |
| *C. molossus* | 31.89202 | -106.4776 |
| *C. molossus* | 31.80967 | -106.5058 |
| *C. molossus* | 32.233196 | -106.5709 |
| *C. molossus* | 30.75667 | -105.0119 |
| *C. molossus* | 31.2104 | -104.8513 |
| *C. molossus* | 31.39692 | -108.3484 |
| *C. molossus* | 29.51085 | -103.3812 |
| *C. molossus* | 33.731926 | -111.3976 |
| *C. molossus* | 31.99583 | -106.0402 |
| *C. molossus* | 32.191345 | -106.564 |
| *C. molossus* | 31.90293 | -106.4491 |
| *C. molossus* | 31.1671 | -104.8434 |
| *C. molossus* | 31.15957 | -104.8365 |
| *C. molossus* | 31.83013 | -106.5065 |
| *C. molossus* | 32.3108 | -110.7167 |
| *C. molossus* | 32.925125 | -107.55 |
| *C. molossus* | 32.009624 | -106.5238 |
| *C. molossus* | 32.897987 | -107.2693 |
| *C. molossus* | 31.90409 | -106.5207 |
| *C. molossus* | 29.44266 | -103.5082 |
| *C. molossus* | 31.39663 | -108.3488 |
| *C. molossus* | 31.88425 | -106.504 |
| *C. molossus* | 31.91477 | -106.5117 |
| *C. molossus* | 29.45074 | -103.3888 |
| *C. molossus* | 32.57178 | -108.47 |
| *C. molossus* | 31.79076 | -106.4755 |
| *C. molossus* | 31.514704 | -109.0252 |
| *C. molossus* | 31.46662 | -111.2326 |
| *C. molossus* | 33.655567 | -112.5092 |
| *C. molossus* | 31.83963 | -106.4846 |
| *C. molossus* | 30.79713 | -105.0113 |
| *C. molossus* | 30.77842 | -105.0146 |
| *C. molossus* | 30.95528 | -98.17438 |
| *C. molossus* | 32.18334 | -109.6422 |
| *C. molossus* | 31.80995 | -106.5056 |
| *C. molossus* | 32.191345 | -106.546 |
| *C. molossus* | 33.731926 | -111.3976 |
| *C. molossus* | 32.2201 | -106.573 |
| *C. molossus* | 32.233177 | -105.571 |
| *C. molossus* | 30.7768 | -105.0162 |
| *C. molossus* | 31.31529 | -104.5273 |
| *C. molossus* | 32.071938 | -105.0921 |
| *C. molossus* | 31.13116 | -104.8073 |
| *C. molossus* | 31.066668 | -105.4667 |
| *C. molossus* | 31.99708 | -105.9045 |
| *C. molossus* | 30.7786 | -105.0178 |
| *C. molossus* | 30.79579 | -105.0155 |
| *C. molossus* | 31.87562 | -106.5517 |
| *C. molossus* | 33.08176 | -112.4935 |
| *C. molossus* | 33.02385 | -108.1306 |
| *C. molossus* | 30.775 | -105.0153 |
| *C. molossus* | 33.46326 | -107.2467 |
| *C. molossus* | 32.4501 | -107.6913 |
| *C. molossus* | 31.4353 | -110.7231 |
| *C. molossus* | 31.53429 | -110.5024 |
| *C. molossus* | 31.89812 | -106.5169 |
| *C. molossus* | 30.76063 | -105.0219 |
| *C. molossus* | 31.61203 | -111.3906 |
| *C. molossus* | 30.44155 | -98.66517 |
| *C. molossus* | 31.892624 | -106.5093 |
| *C. molossus* | 30.68422 | -102.1898 |
| *C. molossus* | 30.52148 | -103.7706 |
| *C. molossus* | 31.9281 | -106.4457 |
| *C. molossus* | 31.83418 | -106.0572 |
| *C. molossus* | 31.90557 | -106.5217 |
| *C. molossus* | 31.91856 | -106.0437 |
| *C. molossus* | 31.89305 | -106.5271 |
| *C. molossus* | 33.18106 | -108.2055 |
| *C. molossus* | 32.40797 | -107.0659 |
| *C. molossus* | 30.78626 | -105.0183 |
| *C. molossus* | 31.48328 | -110.4836 |
| *C. molossus* | 31.4989 | -110.7045 |
| *C. molossus* | 34.46701 | -112.8209 |
| *C. molossus* | 31.55477 | -110.5019 |
| *C. molossus* | 30.86022 | -111.7119 |
| *C. molossus* | 29.79273 | -104.2788 |
| *C. molossus* | 29.79577 | -104.2753 |
| *C. molossus* | 30.03087 | -104.4684 |
| *C. molossus* | 30.88967 | -105.0577 |
| *C. molossus* | 30.90417 | -105.0697 |
| *C. molossus* | 30.92376 | -105.0503 |
| *C. molossus* | 30.29703 | -103.4543 |
| *C. molossus* | 33.0355 | -108.1847 |
| *C. molossus* | 31.91662 | -108.5033 |
| *C. molossus* | 31.13883 | -102.3961 |
| *C. molossus* | 32.75303 | -108.1361 |
| *C. molossus* | 32.82657 | -108.5022 |
| *C. molossus* | 29.9833 | -104.0194 |
| *C. molossus* | 31.91981 | -106.4792 |
| *C. molossus* | 31.83696 | -106.4749 |
| *C. molossus* | 31.88645 | -106.5061 |
| *C. molossus* | 31.77246 | -106.0299 |
| *C. molossus* | 31.83383 | -106.0646 |
| *C. molossus* | 31.8857 | -106.484 |
| *C. molossus* | 32.06944 | -107.0575 |
| *C. molossus* | 31.91477 | -106.5034 |
| *C. molossus* | 31.6917 | -106.0001 |
| *C. molossus* | 29.51667 | -106.8 |
| *C. molossus* | 31.87287 | -106.4979 |
| *C. molossus* | 31.73531 | -106.008 |
| *C. molossus* | 31.78894 | -106.0214 |
| *C. molossus* | 31.70003 | -105.9939 |
| *C. molossus* | 33.125526 | -108.2679 |
| *C. molossus* | 23.81 | -105.16 |
| *C. molossus* | 23.13 | -102.48 |
| *C. molossus* | 23.88 | -101.73 |
| *C. molossus* | 31.83529 | -106.4773 |
| *C. molossus* | 32.5681 | -105.7377 |
| *C. molossus* | 32.5824 | -105.7206 |
| *C. molossus* | 31.89371 | -106.4718 |
| *C. molossus* | 31.45951 | -109.3702 |
| *C. molossus* | 31.7957 | -106.0491 |
| *C. molossus* | 31.95707 | -106.4896 |
| *C. molossus* | 30.84309 | -104.1509 |
| *C. molossus* | 30.6059 | -103.9738 |
| *C. molossus* | 31.90753 | -106.5267 |
| *C. molossus* | 31.88343 | -106.4882 |
| *C. molossus* | 31.94225 | -104.7185 |
| *C. molossus* | 30.97316 | -105.499 |
| *C. molossus* | 31.90702 | -106.5239 |
| *C. molossus* | 31.876947 | -107.0115 |
| *C. molossus* | 31.84385 | -106.5149 |
| *C. molossus* | 23.9 | -103.45 |
| *C. molossus* | 23.85 | -101.73 |
| *C. molossus* | 32.83 | -108.24 |
| *C. molossus* | 32.55 | -108.52 |
| *C. molossus* | 31.63 | -108.35 |
| *C. molossus* | 32.188408 | -104.3493 |
| *C. molossus* | 32.6253 | -108.6218 |
| *C. molossus* | 32.653793 | -108.5192 |
| *C. molossus* | 32.65 | -108.849 |
| *C. molossus* | 32.119 | -104.929 |
| *C. molossus* | 31.7781 | -110.8183 |
| *C. molossus* | 29.8083 | -101.4667 |
| *C. molossus* | 30.725904 | -104.2145 |
| *C. pricei* | 23.68 | -105.63 |
| *C. pricei* | 23.7 | -105.72 |
| *C. pricei* | 23.68 | -105.73 |
| *C. pricei* | 23.91 | -105.03 |
| *C. pricei* | 23.76 | -105.74 |
| *C. pricei* | 31.70007 | -110.8475 |
| *C. pricei* | 23.75 | -105.5333 |
| *C. pricei* | 31.725088 | -110.88 |
| *C. pricei* | 31.9151 | -109.2845 |
| *C. pricei* | 23.391 | -104.247 |
| *C. pricei* | 22.59 | -104.585 |
| *C. pricei* | 23.437 | -104.279 |
| *C. pricei* | 23.916666 | -104.9667 |
| *C. pricei* | 23.88 | -105.08 |
| *C. pricei* | 29.96667 | -108.3333 |
| *C. pricei* | 30.97471 | -110.0194 |
| *C. pricei* | 23.89 | -105.08 |
| *C. pricei* | 30.08333 | -108.35 |
| *C. pricei* | 28.63333 | -106.0667 |
| *C. pricei* | 31.291666 | -107.3 |
| *C. pricei* | 24.573359 | -100.007 |
| *C. pricei* | 31.9164 | -109.2643 |
| *C. pricei* | 31.927 | -109.271 |
| *C. pricei* | 26.82155 | -107.0805 |
| *C. pricei* | 31.4567 | -110.2389 |
| *C. pricei* | 23.694082 | -105.7271 |
| *C. pricei* | 31.915 | -109.2839 |
| *C. pricei* | 31.9588 | -109.3137 |
| *C. pricei* | 31.8961 | -109.275 |
| *C. pricei* | 31.9033 | -109.275 |
| *C. pricei* | 29.392097 | -106.8987 |
| *C. pricei* | 31.9333 | -109.2628 |
| *C. pricei* | 31.9208 | -109.28 |
| *C. pricei* | 24.56 | -100.02 |
| *C. pricei* | 26.91 | -107.07 |
| *C. pricei* | 26.42 | -106.48 |
| *C. pricei* | 23.7 | -105.59 |
| *C. pricei* | 23.67 | -105.74 |
| *C. pricei* | 29.45 | -106.87 |
| *C. pricei* | 23.73 | -105.69 |
| *C. pricei* | 23.87 | -99.85 |
| *C. pricei* | 31.51 | -110.36 |
| *C. pricei* | 24.59 | -100 |
| *C. pricei* | 23.67 | -104.82 |
| *C. pricei* | 23.64 | -105.84 |
| *C. pricei* | 31.90361 | -109.275 |
| *C. pricei* | 27.3 | -107.5417 |
| *C. pricei* | 25.27 | -100.43 |
| *C. pricei* | 25.27 | -100.41 |
| *C. pricei* | 25.43 | -104.3 |
| *C. pricei* | 31.916666 | -109.3667 |
| *C. pricei* | 24.827 | -100.076 |
| *C. pricei* | 24.327 | -99.912 |
| *C. pricei* | 24.876 | -100.22 |
| *C. pricei* | 27.42 | -107.35 |
| *C. pricei* | 28.10361 | -108.1108 |
| *C. pricei* | 27.85806 | -107.9939 |
| *C. pricei* | 23.82056 | -105.3372 |
| *C. pricei* | 24.09556 | -105.5492 |
| *C. pricei* | 24.12 | -105.56 |
| *C. pricei* | 24.21 | -105.95 |
| *C. pricei* | 23.572 | -99.722 |
| *C. pricei* | 31.916515 | -109.2767 |
| *C. pricei* | 23.38 | -104.96 |
| *C. pricei* | 26.27 | -106.51 |
| *C. pricei* | 31.89078 | -109.2821 |
| *C. pricei* | 23.81 | -105.16 |
| *C. pricei* | 22.62 | -104.26 |
| *C. pricei* | 29.95 | -108.4 |
| *C. pricei* | 25.63 | -106.93 |
| *C. pricei* | 25.58 | -106.93 |
| *C. pricei* | 23.8 | -105.15 |
| *C. pricei* | 26.12 | -106.75 |
| *C. pricei* | 23.81 | -105.13 |
| *C. pricei* | 29.96 | -108.42 |
| *C. pricei* | 25.56 | -106.88 |
| *C. ravus* | 16.56 | -96.7 |
| *C. ravus* | 18.716667 | -97.3 |
| *C. ravus* | 19.351667 | -98.67028 |
| *C. ravus* | 19.127222 | -98.74389 |
| *C. ravus* | 16.96 | -96.48 |
| *C. ravus* | 19.333334 | -98.195 |
| *C. ravus* | 17.97 | -96.47 |
| *C. ravus* | 19.29 | -98.33 |
| *C. ravus* | 18.565966 | -97.19573 |
| *C. ravus* | 19.195278 | -98.10278 |
| *C. ravus* | 17.4025 | -96.51417 |
| *C. ravus* | 17.55139 | -99.50083 |
| *C. ravus* | 19.19 | -98.1 |
| *C. ravus* | 17.46 | -96.49 |
| *C. ravus* | 17.57 | -99.56 |
| *C. ravus* | 18.351 | -97.443 |
| *C. ravus* | 19.135 | -99.29167 |
| *C. ravus* | 19.125 | -98.77361 |
| *C. ravus* | 17.55667 | -99.68556 |
| *C. ravus* | 17.56 | -99.64 |
| *C. ravus* | 19.11 | -98.04 |
| *C. ravus* | 19.75 | -98.03 |
| *C. ravus* | 19.01 | -99.37 |
| *C. ravus* | 19 | -99.19 |
| *C. ravus* | 19.12778 | -98.76278 |
| *C. ravus* | 19.02 | -99.27 |
| *C. ravus* | 18.3 | -97.5 |
| *C. ravus* | 19.05 | -99.31 |
| *C. ravus* | 18.78333 | -98.71667 |
| *C. ravus* | 19.03 | -99.28 |
| *C. ravus* | 19.12 | -98.63 |
| *C. ravus* | 19.121944 | -98.73111 |
| *C. ravus* | 19.02 | -99.25 |
| *C. ravus* | 19.27 | -98.3 |
| *C. ravus* | 19.33 | -98.64 |
| *C. ravus* | 19.135556 | -98.69056 |
| *C. ravus* | 19.40611 | -97.39611 |
| *C. ravus* | 19.06 | -99.46 |
| *C. ravus* | 19.063889 | -99.84583 |
| *C. ravus* | 18.71972 | -97.29667 |
| *C. ravus* | 19.41556 | -98.14 |
| *C. ravus* | 19.21 | -99.2 |
| *C. ravus* | 19.34 | -98.66 |
| *C. ravus* | 18.97583 | -98.23056 |
| *C. ravus* | 19.33333 | -99.21389 |
| *C. ravus* | 19.666667 | -99.555 |
| *C. ravus* | 19.31 | -98.88167 |
| *C. ravus* | 18.957778 | -99.42417 |
| *C. ravus* | 19.761667 | -98.64833 |
| *C. ravus* | 19.035556 | -99.38 |
| *C. ravus* | 18.33 | -97.508 |
| *C. ravus* | 19.4 | -97.6667 |
| *C. ruber* | 29.24467 | -113.3456 |
| *C. ruber* | 24.208332 | -110.085 |
| *C. ruber* | 28.63107 | -112.8173 |
| *C. ruber* | 31.86359 | -116.6108 |
| *C. ruber* | 25.79 | -111.25 |
| *C. ruber* | 24.333334 | -110.9833 |
| *C. ruber* | 24.1 | -110.6833 |
| *C. ruber* | 26.63348 | -111.8295 |
| *C. ruber* | 23.8 | -109.9333 |
| *C. ruber* | 23.85429 | -110.2516 |
| *C. ruber* | 23.91 | -110.26 |
| *C. ruber* | 23.84871 | -110.2512 |
| *C. ruber* | 23.83 | -110.18 |
| *C. ruber* | 23.82 | -110.17 |
| *C. ruber* | 26.18836 | -111.4391 |
| *C. ruber* | 26.84154 | -111.9054 |
| *C. ruber* | 29.983334 | -115.6 |
| *C. ruber* | 28.02724 | -113.4653 |
| *C. ruber* | 29.27582 | -114.2107 |
| *C. ruber* | 29.95364 | -115.1562 |
| *C. ruber* | 23.73998 | -110.0578 |
| *C. ruber* | 26.058332 | -111.8233 |
| *C. ruber* | 24.02448 | -110.2963 |
| *C. ruber* | 33.114666 | -116.4429 |
| *C. ruber* | 30.95212 | -114.7707 |
| *C. ruber* | 30.91452 | -114.7193 |
| *C. ruber* | 33.139732 | -116.5343 |
| *C. ruber* | 30.0404 | -115.7873 |
| *C. ruber* | 30.02327 | -115.75 |
| *C. ruber* | 31.88865 | -116.4882 |
| *C. ruber* | 23.68351 | -109.7237 |
| *C. ruber* | 32.813503 | -117.2406 |
| *C. ruber* | 32.828117 | -117.2573 |
| *C. ruber* | 33.162933 | -116.3365 |
| *C. ruber* | 33.353 | -117.0269 |
| *C. ruber* | 32.67925 | -116.6701 |
| *C. ruber* | 33.008434 | -116.4555 |
| *C. ruber* | 33.063423 | -117.1243 |
| *C. ruber* | 33.135235 | -116.3515 |
| *C. ruber* | 32.88365 | -116.8222 |
| *C. ruber* | 33.944168 | -116.6422 |
| *C. ruber* | 33.95532 | -116.6455 |
| *C. ruber* | 33.67222 | -117.064 |
| *C. ruber* | 33.370678 | -117.3232 |
| *C. ruber* | 33.1015 | -116.4653 |
| *C. ruber* | 32.97203 | -116.3907 |
| *C. ruber* | 33.203335 | -116.9758 |
| *C. ruber* | 33.205555 | -116.9778 |
| *C. ruber* | 32.663887 | -116.9775 |
| *C. ruber* | 23.44 | -109.78 |
| *C. ruber* | 28.64 | -112.83 |
| *C. ruber* | 27.22271 | -112.0727 |
| *C. ruber* | 32.962776 | -117.035 |
| *C. ruber* | 25.68335 | -111.0342 |
| *C. ruber* | 24.98 | -110.615 |
| *C. ruber* | 24.1424 | -110.3109 |
| *C. ruber* | 25.48879 | -111.9185 |
| *C. ruber* | 30.468332 | -115.965 |
| *C. ruber* | 27.69167 | -114.8957 |
| *C. ruber* | 24.43412 | -111.8211 |
| *C. ruber* | 28.19511 | -115.2102 |
| *C. ruber* | 32.10854 | -116.884 |
| *C. ruber* | 33.74338 | -117.1077 |
| *C. ruber* | 33.13748 | -116.3563 |
| *C. ruber* | 33.2539 | -117.2425 |
| *C. ruber* | 32.959446 | -117.2644 |
| *C. ruber* | 32.597065 | -116.6783 |
| *C. ruber* | 33.03449 | -116.6991 |
| *C. ruber* | 32.836784 | -117.0076 |
| *C. ruber* | 27.2 | -113.22 |
| *C. ruber* | 23.44289 | -109.7067 |
| *C. ruber* | 23.44857 | -110.2233 |
| *C. ruber* | 23.97293 | -109.9382 |
| *C. ruber* | 33.894444 | -117.4181 |
| *C. ruber* | 29.89485 | -114.8927 |
| *C. ruber* | 30.29213 | -115.8041 |
| *C. ruber* | 30.533333 | -115.9333 |
| *C. ruber* | 25.68153 | -111.0479 |
| *C. ruber* | 28.033333 | -113.4 |
| *C. ruber* | 31.033333 | -115.2333 |
| *C. ruber* | 23.63 | -109.66 |
| *C. ruber* | 33.1192 | -116.435 |
| *C. ruber* | 33.131485 | -116.4041 |
| *C. ruber* | 32.71575 | -116.7668 |
| *C. ruber* | 32.714077 | -116.7586 |
| *C. ruber* | 32.71463 | -116.7698 |
| *C. ruber* | 33.336666 | -116.2783 |
| *C. ruber* | 33.287643 | -116.4011 |
| *C. ruber* | 32.811295 | -117.0216 |
| *C. ruber* | 23.686666 | -109.815 |
| *C. ruber* | 33.436977 | -117.3245 |
| *C. ruber* | 33.810276 | -116.675 |
| *C. ruber* | 23.066668 | -109.7 |
| *C. ruber* | 30.74333 | -115.9636 |
| *C. ruber* | 30.816668 | -115.6667 |
| *C. ruber* | 33.360584 | -116.4022 |
| *C. ruber* | 32.7592 | -117.0328 |
| *C. ruber* | 27.501 | -112.669 |
| *C. ruber* | 31.07 | -116.211 |
| *C. ruber* | 25.8 | -111.334 |
| *C. ruber* | 33.352444 | -117.1636 |
| *C. ruber* | 32.927856 | -117.2563 |
| *C. ruber* | 33.92343 | -117.8017 |
| *C. ruber* | 33.54234 | -117.7495 |
| *C. ruber* | 33.66829 | -117.7886 |
| *C. ruber* | 33.67531 | -117.5214 |
| *C. ruber* | 33.66254 | -117.5904 |
| *C. ruber* | 33.75703 | -117.6984 |
| *C. ruber* | 33.73281 | -117.3621 |
| *C. ruber* | 33.71165 | -117.3621 |
| *C. ruber* | 33.53956 | -117.214 |
| *C. ruber* | 33.48487 | -117.0439 |
| *C. ruber* | 33.98193 | -116.3632 |
| *C. ruber* | 33.84498 | -116.5852 |
| *C. ruber* | 33.99083 | -116.5812 |
| *C. ruber* | 33.97338 | -116.7207 |
| *C. ruber* | 34.0247 | -116.4254 |
| *C. ruber* | 33.90085 | -116.6785 |
| *C. ruber* | 33.94347 | -116.6418 |
| *C. ruber* | 33.95747 | -116.6454 |
| *C. ruber* | 33.95202 | -116.6443 |
| *C. ruber* | 33.96162 | -116.65 |
| *C. ruber* | 33.97876 | -116.653 |
| *C. ruber* | 33.95627 | -116.6452 |
| *C. ruber* | 33.90461 | -116.6449 |
| *C. ruber* | 33.95837 | -116.6472 |
| *C. ruber* | 33.95818 | -116.6465 |
| *C. ruber* | 33.93882 | -116.6417 |
| *C. ruber* | 33.96878 | -116.652 |
| *C. ruber* | 33.92641 | -116.6417 |
| *C. ruber* | 33.93181 | -116.6417 |
| *C. ruber* | 33.93306 | -116.6414 |
| *C. ruber* | 33.93945 | -116.6417 |
| *C. ruber* | 33.95339 | -116.6449 |
| *C. ruber* | 33.95896 | -116.648 |
| *C. ruber* | 33.96536 | -116.6515 |
| *C. ruber* | 33.98019 | -116.6537 |
| *C. ruber* | 34.05065 | -116.6886 |
| *C. ruber* | 33.10754 | -116.451 |
| *C. ruber* | 33.13325 | -116.3361 |
| *C. ruber* | 31.75442 | -116.5854 |
| *C. ruber* | 31.718332 | -116.6617 |
| *C. ruber* | 30.616667 | -114.7167 |
| *C. ruber* | 31.388332 | -115.78 |
| *C. ruber* | 30.21 | -115.7133 |
| *C. ruber* | 26.67218 | -111.8967 |
| *C. ruber* | 26.02674 | -111.8215 |
| *C. ruber* | 23.55 | -109.9667 |
| *C. ruber* | 25.750935 | -112.0061 |
| *C. ruber* | 22.97073 | -110.0181 |
| *C. ruber* | 24.07468 | -110.2022 |
| *C. ruber* | 23.83527 | -110.1886 |
| *C. ruber* | 33.86204 | -116.689 |
| *C. ruber* | 33.82753 | -117.486 |
| *C. ruber* | 33.81448 | -117.3583 |
| *C. ruber* | 33.94728 | -116.6041 |
| *C. ruber* | 33.13531 | -116.2985 |
| *C. ruber* | 34.00534 | -117.2465 |
| *C. ruber* | 33.47895 | -116.8482 |
| *C. ruber* | 33.8094 | -117.4095 |
| *C. ruber* | 23.85596 | -110.2524 |
| *C. ruber* | 33.95139 | -116.6441 |
| *C. ruber* | 33.94997 | -116.644 |
| *C. ruber* | 33.67145 | -117.7504 |
| *C. ruber* | 33.32692 | -117.0747 |
| *C. ruber* | 33.93232 | -117.5001 |
| *C. ruber* | 34.004066 | -117.1673 |
| *C. ruber* | 33.33257 | -117.3277 |
| *C. ruber* | 33.15285 | -116.1812 |
| *C. ruber* | 33.20104 | -116.3303 |
| *C. ruber* | 32.88665 | -116.9328 |
| *C. ruber* | 33.15613 | -117.1043 |
| *C. ruber* | 33.32998 | -117.1601 |
| *C. ruber* | 33.77978 | -116.4654 |
| *C. ruber* | 33.97045 | -116.5874 |
| *C. ruber* | 33.6681 | -117.3273 |
| *C. ruber* | 34.41925 | -116.7494 |
| *C. ruber* | 31.2953 | -116.2213 |
| *C. ruber* | 30.40118 | -115.9114 |
| *C. ruber* | 23.48333 | -109.7167 |
| *C. ruber* | 33.14618 | -116.3776 |
| *C. ruber* | 33.57492 | -117.5402 |
| *C. ruber* | 33.96626 | -116.6491 |
| *C. ruber* | 33.944 | -116.5012 |
| *C. ruber* | 33.79435 | -117.6556 |
| *C. ruber* | 33.74752 | -116.972 |
| *C. ruber* | 33.66585 | -116.4044 |
| *C. ruber* | 33.87577 | -116.6732 |
| *C. ruber* | 33.92504 | -116.6384 |
| *C. ruber* | 34.09178 | -116.6111 |
| *C. ruber* | 32.61116 | -116.4689 |
| *C. ruber* | 30.37633 | -115.8455 |
| *C. ruber* | 27.683332 | -114.9 |
| *C. ruber* | 27.765 | -114.8955 |
| *C. ruber* | 27.72243 | -113.3957 |
| *C. ruber* | 31.361668 | -115.72 |
| *C. ruber* | 31.555 | -116.21 |
| *C. ruber* | 32.71 | -115.35 |
| *C. ruber* | 31.33364 | -116.3229 |
| *C. ruber* | 32.268333 | -117.02 |
| *C. ruber* | 23.9936 | -109.8256 |
| *C. ruber* | 33.1115 | -116.4466 |
| *C. ruber* | 33.97079 | -117.2032 |
| *C. ruber* | 33.93227 | -116.6416 |
| *C. ruber* | 33.73535 | -117.3452 |
| *C. ruber* | 33.72228 | -117.5939 |
| *C. ruber* | 33.71446 | -117.624 |
| *C. ruber* | 33.10055 | -116.4605 |
| *C. ruber* | 33.10398 | -116.4534 |
| *C. ruber* | 22.98082 | -109.7668 |
| *C. ruber* | 23.272156 | -109.7454 |
| *C. ruber* | 32.6401 | -117.0842 |
| *C. ruber* | 32.78687 | -116.7851 |
| *C. ruber* | 33.95339 | -117.3962 |
| *C. ruber* | 34.12701 | -116.8932 |
| *C. ruber* | 34.117012 | -117.2743 |
| *C. ruber* | 33.9854 | -116.6533 |
| *C. ruber* | 27.281944 | -112.8958 |
| *C. ruber* | 23.45 | -110.2167 |
| *C. ruber* | 23.178333 | -109.7011 |
| *C. ruber* | 23.441944 | -109.7747 |
| *C. ruber* | 23.6 | -109.6 |
| *C. ruber* | 25.34361 | -111.9375 |
| *C. ruber* | 28.316668 | -113.5089 |
| *C. ruber* | 33.879467 | -116.6869 |
| *C. ruber* | 31.651667 | -116.2672 |
| *C. ruber* | 33.30349 | -116.2603 |
| *C. ruber* | 33.9417 | -116.6408 |
| *C. ruber* | 27.409166 | -112.5931 |
| *C. ruber* | 31.31639 | -115.2203 |
| *C. ruber* | 29.434444 | -114.4172 |
| *C. ruber* | 27.506111 | -112.7264 |
| *C. ruber* | 23.845 | -110.2033 |
| *C. ruber* | 23.835833 | -110.19 |
| *C. ruber* | 23.809168 | -110.2678 |
| *C. ruber* | 23.19 | -109.72 |
| *C. ruber* | 23.787222 | -110.2403 |
| *C. ruber* | 23.871668 | -110.1544 |
| *C. ruber* | 23.952778 | -110.2836 |
| *C. ruber* | 23.901667 | -110.25 |
| *C. ruber* | 23.98 | -110.2867 |
| *C. ruber* | 23.8 | -110.25 |
| *C. ruber* | 23.773056 | -110.2261 |
| *C. ruber* | 23.469444 | -110.2175 |
| *C. ruber* | 23.89 | -110.26 |
| *C. ruber* | 23.6 | -110.11 |
| *C. ruber* | 23.958332 | -110.2889 |
| *C. ruber* | 23.935556 | -110.2695 |
| *C. ruber* | 23.895555 | -110.27 |
| *C. ruber* | 23.806667 | -110.2597 |
| *C. ruber* | 23.998056 | -110.1544 |
| *C. ruber* | 23.789167 | -110.2422 |
| *C. ruber* | 23.941668 | -110.2597 |
| *C. ruber* | 23.78 | -110.25 |
| *C. ruber* | 23.866667 | -110.1111 |
| *C. ruber* | 23.872223 | -110.1097 |
| *C. ruber* | 23.88889 | -110.1544 |
| *C. ruber* | 23.77 | -110.0558 |
| *C. ruber* | 24.06 | -110.1544 |
| *C. ruber* | 27.756945 | -113.4422 |
| *C. ruber* | 27.755 | -113.5233 |
| *C. ruber* | 24.24 | -110.29 |
| *C. ruber* | 33.716976 | -116.4011 |
| *C. ruber* | 33.61934 | -116.4293 |
| *C. ruber* | 33.956882 | -116.6446 |
| *C. ruber* | 32.720695 | -116.8324 |
| *C. ruber* | 32.775 | -116.7263 |
| *C. ruber* | 32.97078 | -116.369 |
| *C. ruber* | 32.64 | -116.78 |
| *C. ruber* | 28.080833 | -115.1956 |
| *C. ruber* | 28.95389 | -114.1544 |
| *C. ruber* | 30.147223 | -115.7781 |
| *C. ruber* | 23.140278 | -109.6942 |
| *C. ruber* | 28.1175 | -115.1914 |
| *C. ruber* | 33.67756 | -116.4018 |
| *C. ruber* | 32.97 | -116.35 |
| *C. ruber* | 32.975174 | -116.4098 |
| *C. ruber* | 33.23478 | -116.746 |
| *C. ruber* | 29.966667 | -115.1167 |
| *C. ruber* | 24.09 | -110.45 |
| *C. ruber* | 24.074444 | -110.6139 |
| *C. ruber* | 24 | -110.45 |
| *C. ruber* | 25.883333 | -112.0345 |
| *C. ruber* | 28.621756 | -112.8035 |
| *C. ruber* | 31.901667 | -116.6844 |
| *C. ruber* | 33.934254 | -116.8721 |
| *C. ruber* | 33.09057 | -116.9513 |
| *C. ruber* | 33.451374 | -116.923 |
| *C. ruber* | 33.860977 | -117.0128 |
| *C. ruber* | 33.884808 | -116.7849 |
| *C. ruber* | 33.119 | -117.086 |
| *C. ruber* | 30.85 | -115.2111 |
| *C. ruber* | 29.316668 | -113.4167 |
| *C. ruber* | 30.966667 | -116.1 |
| *C. ruber* | 33.14962 | -116.2584 |
| *C. ruber* | 31.734444 | -116.3689 |
| *C. ruber* | 29.73278 | -114.7181 |
| *C. ruber* | 33.2823 | -116.6337 |
| *C. ruber* | 27.67246 | -112.8884 |
| *C. ruber* | 29.4019 | -114.4031 |
| *C. ruber* | 33.78252 | -117.2286 |
| *C. ruber* | 33.36268 | -117.1629 |
| *C. ruber* | 33.75103 | -116.2046 |
| *C. ruber* | 33.84094 | -117.4332 |
| *C. ruber* | 33.83125 | -117.3938 |
| *C. ruber* | 33.270832 | -116.4125 |
| *C. ruber* | 32.82636 | -117.0534 |
| *C. ruber* | 22.88833 | -109.915 |
| *C. ruber* | 32.78496 | -117.1106 |
| *C. ruber* | 33.22118 | -116.3342 |
| *C. ruber* | 33.13892 | -116.3862 |
| *C. ruber* | 32.6727 | -117.0236 |
| *C. ruber* | 33.10922 | -116.6722 |
| *C. ruber* | 33.91751 | -117.1578 |
| *C. ruber* | 32.73164 | -117.0034 |
| *C. ruber* | 32.67778 | -116.7271 |
| *C. ruber* | 32.73057 | -116.4937 |
| *C. ruber* | 32.87304 | -117.15 |
| *C. ruber* | 32.83252 | -116.9165 |
| *C. ruber* | 32.94942 | -116.8775 |
| *C. ruber* | 33.25095 | -117.131 |
| *C. ruber* | 30.02602 | -115.5323 |
| *C. ruber* | 30.07501 | -115.6384 |
| *C. ruber* | 32.35167 | -117 |
| *C. ruber* | 33.14124 | -117.1633 |
| *C. ruber* | 31.38967 | -115.7759 |
| *C. ruber* | 32.7768 | -115.9047 |
| *C. ruber* | 33.29137 | -116.4884 |
| *C. ruber* | 32.82314 | -117.1698 |
| *C. ruber* | 33.12724 | -116.5276 |
| *C. ruber* | 24.35 | -111.01 |
| *C. ruber* | 26.78824 | -111.8905 |
| *C. ruber* | 28.3087 | -112.8751 |
| *C. ruber* | 33.37045 | -117.0747 |
| *C. ruber* | 33.37643 | -117.2503 |
| *C. ruber* | 33.04895 | -116.4147 |
| *C. ruber* | 31.95324 | -116.2924 |
| *C. ruber* | 32.89665 | -116.2623 |
| *C. ruber* | 31.52026 | -116.327 |
| *C. ruber* | 29.97056 | -115.2364 |
| *C. ruber* | 30.67589 | -115.9787 |
| *C. ruber* | 31.71833 | -116.6617 |
| *C. ruber* | 30.24642 | -115.7909 |
| *C. ruber* | 29.03165 | -113.8962 |
| *C. ruber* | 33.02035 | -117.2029 |
| *C. ruber* | 32.96282 | -117.0359 |
| *C. ruber* | 31.38833 | -115.78 |
| *C. ruber* | 22.88821 | -109.9152 |
| *C. ruber* | 30.97167 | -116.09 |
| *C. ruber* | 32.67983 | -116.857 |
| *C. ruber* | 30.96667 | -115.75 |
| *C. ruber* | 33.75002 | -116.4804 |
| *C. ruber* | 32.75442 | -117.0294 |
| *C. ruber* | 32.97284 | -116.9169 |
| *C. ruber* | 32.88433 | -116.8168 |
| *C. ruber* | 32.80515 | -116.949 |
| *C. ruber* | 33.0334 | -117.1129 |
| *C. ruber* | 32.75442 | -116.9951 |
| *C. ruber* | 23.6 | -109.5833 |
| *C. ruber* | 33.04308 | -117.2586 |
| *C. ruber* | 33.59251 | -117.4962 |
| *C. ruber* | 32.98218 | -116.9788 |
| *C. ruber* | 32.85326 | -116.617 |
| *C. ruber* | 33.02177 | -117.2813 |
| *C. ruber* | 33.1465 | -116.3518 |
| *C. ruber* | 33.23587 | -117.3859 |
| *C. ruber* | 32.6791 | -116.6705 |
| *C. ruber* | 33.00867 | -116.9707 |
| *C. ruber* | 32.90838 | -116.9262 |
| *C. ruber* | 32.78174 | -117.0459 |
| *C. ruber* | 32.75867 | -116.9006 |
| *C. ruber* | 32.59278 | -117.0111 |
| *C. ruber* | 32.62988 | -117.0016 |
| *C. ruber* | 32.80428 | -116.8646 |
| *C. ruber* | 32.56844 | -117.0035 |
| *C. ruber* | 33.11859 | -116.7884 |
| *C. ruber* | 32.83845 | -116.9732 |
| *C. ruber* | 26.30167 | -111.775 |
| *C. ruber* | 23.80313 | -110.1062 |
| *C. ruber* | 33.11458 | -116.443 |
| *C. ruber* | 32.85505 | -116.8526 |
| *C. ruber* | 32.9509 | -117.2386 |
| *C. ruber* | 32.77333 | -117.1405 |
| *C. ruber* | 32.80403 | -117.2482 |
| *C. ruber* | 32.96998 | -116.3789 |
| *C. ruber* | 33.38922 | -117.1926 |
| *C. ruber* | 27.15962 | -112.156 |
| *C. ruber* | 27.28194 | -112.8958 |
| *C. ruber* | 25.93465 | -111.4328 |
| *C. ruber* | 32.64425 | -116.7814 |
| *C. ruber* | 33.11921 | -117.0864 |
| *C. ruber* | 32.62925 | -116.4606 |
| *C. ruber* | 33.94669 | -117.2965 |
| *C. ruber* | 32.9506 | -116.3029 |
| *C. ruber* | 33.05442 | -117.0901 |
| *C. ruber* | 32.60481 | -116.6131 |
| *C. ruber* | 32.59474 | -117.0644 |
| *C. ruber* | 23.99 | -109.83 |
| *C. ruber* | 32.67318 | -116.1098 |
| *C. ruber* | 32.16272 | -116.8982 |
| *C. ruber* | 33.14269 | -116.2779 |
| *C. ruber* | 32.81541 | -116.8358 |
| *C. ruber* | 32.83257 | -117.2686 |
| *C. ruber* | 32.82358 | -116.8532 |
| *C. ruber* | 33.13276 | -116.3308 |
| *C. ruber* | 33.04542 | -117.1285 |
| *C. ruber* | 33.13512 | -116.3805 |
| *C. ruber* | 32.85319 | -117.0074 |
| *C. ruber* | 33.14313 | -116.3608 |
| *C. ruber* | 32.31 | -116.945 |
| *C. ruber* | 33.58283 | -116.9697 |
| *C. ruber* | 33.13311 | -116.3136 |
| *C. ruber* | 31.02948 | -115.8164 |
| *C. ruber* | 33.00872 | -116.9706 |
| *C. ruber* | 33.21839 | -117.0342 |
| *C. ruber* | 31.14924 | -116.1356 |
| *C. ruber* | 30.92298 | -114.7242 |
| *C. ruber* | 32.63769 | -116.6888 |
| *C. ruber* | 28.9454 | -113.5591 |
| *C. ruber* | 33.30776 | -117.1206 |
| *C. ruber* | 32.85838 | -116.9127 |
| *C. ruber* | 33.96443 | -117.3365 |
| *C. ruber* | 32.78179 | -116.8938 |
| *C. ruber* | 32.78435 | -117.1727 |
| *C. ruber* | 33.06693 | -117.0001 |
| *C. ruber* | 32.60647 | -116.4689 |
| *C. ruber* | 27.84833 | -115.0767 |
| *C. ruber* | 31.325 | -116.25 |
| *C. ruber* | 31.97593 | -116.7753 |
| *C. ruber* | 32.77817 | -117.2527 |
| *C. ruber* | 33.13604 | -116.3703 |
| *C. ruber* | 32.77402 | -117.0735 |
| *C. ruber* | 32.57223 | -116.6403 |
| *C. ruber* | 30.92608 | -116.1288 |
| *C. ruber* | 32.3554 | -116.6144 |
| *C. ruber* | 33.23667 | -117.0241 |
| *C. ruber* | 31.43377 | -116.3129 |
| *C. ruber* | 30.48333 | -115.9167 |
| *C. ruber* | 33.1889 | -116.3878 |
| *C. ruber* | 31.08 | -116.2333 |
| *C. ruber* | 31.35816 | -116.2707 |
| *C. ruber* | 31.63846 | -116.5118 |
| *C. ruber* | 31.07382 | -116.2087 |
| *C. ruber* | 31.35 | -116.2667 |
| *C. ruber* | 32.77299 | -117.0382 |
| *C. ruber* | 30.94691 | -116.144 |
| *C. ruber* | 30.73333 | -115.9833 |
| *C. ruber* | 30.07061 | -115.6174 |
| *C. ruber* | 26.25 | -112.4833 |
| *C. ruber* | 30.05 | -115.5667 |
| *C. ruber* | 32.90471 | -117.1474 |
| *C. ruber* | 31.31247 | -116.4289 |
| *C. ruber* | 33.09171 | -116.9539 |
| *C. ruber* | 32.83091 | -117.2636 |
| *C. ruber* | 28.24871 | -113.8063 |
| *C. ruber* | 34.03745 | -117.2215 |
| *C. ruber* | 32.73325 | -116.9407 |
| *C. ruber* | 32.66747 | -116.8224 |
| *C. ruber* | 30.17407 | -115.7909 |
| *C. ruber* | 30.7978 | -116.029 |
| *C. ruber* | 30.14505 | -115.7715 |
| *C. ruber* | 29.94884 | -114.6318 |
| *C. ruber* | 31.32485 | -116.2826 |
| *C. ruber* | 30.96905 | -115.9092 |
| *C. ruber* | 33.13399 | -116.3648 |
| *C. ruber* | 32.79073 | -117.0138 |
| *C. ruber* | 32.61145 | -116.7072 |
| *C. ruber* | 33.11918 | -116.4341 |
| *C. ruber* | 30.46288 | -115.9464 |
| *C. ruber* | 31.15118 | -116.1364 |
| *C. ruber* | 32.82837 | -116.1678 |
| *C. ruber* | 33.20282 | -117.067 |
| *C. ruber* | 33.13379 | -116.34 |
| *C. ruber* | 32.81116 | -116.8634 |
| *C. ruber* | 32.9573 | -116.8643 |
| *C. ruber* | 33.14254 | -117.3276 |
| *C. ruber* | 32.97617 | -116.3552 |
| *C. ruber* | 32.69621 | -116.9361 |
| *C. ruber* | 33.95764 | -117.3088 |
| *C. ruber* | 31.57333 | -116.4267 |
| *C. ruber* | 32.81478 | -117.2113 |
| *C. ruber* | 32.78144 | -116.8531 |
| *C. ruber* | 33.35435 | -117.4235 |
| *C. ruber* | 26.0203 | -112.1095 |
| *C. ruber* | 33.25642 | -116.3847 |
| *C. ruber* | 33.31971 | -116.3502 |
| *C. ruber* | 33.21272 | -116.2126 |
| *C. ruber* | 25.81667 | -111.3167 |
| *C. ruber* | 26.51667 | -111.4467 |
| *C. ruber* | 23.24139 | -109.6998 |
| *C. ruber* | 29.78333 | -115.5333 |
| *C. ruber* | 29.96833 | -115.23 |
| *C. ruber* | 29.96667 | -115.55 |
| *C. ruber* | 28.53333 | -114.0833 |
| *C. ruber* | 28.21667 | -114.0333 |
| *C. ruber* | 29.95644 | -115.8082 |
| *C. ruber* | 29.58333 | -115.2333 |
| *C. ruber* | 29.55 | -115.2667 |
| *C. ruber* | 32.84181 | -116.6953 |
| *C. ruber* | 30.47863 | -115.9165 |
| *C. ruber* | 23.70167 | -110.48 |
| *C. ruber* | 33.16053 | -116.5018 |
| *C. ruber* | 32.6529 | -117.0514 |
| *C. ruber* | 33.48832 | -117.3612 |
| *C. ruber* | 31.12667 | -116.1317 |
| *C. ruber* | 30.97325 | -116.28 |
| *C. ruber* | 26.85396 | -112.06 |
| *C. ruber* | 26.84729 | -112.0748 |
| *C. ruber* | 23.44837 | -110.034 |
| *C. ruber* | 23.49225 | -109.7174 |
| *C. ruber* | 33.11185 | -116.523 |
| *C. ruber* | 33.30051 | -116.9134 |
| *C. ruber* | 33.12968 | -117.2668 |
| *C. ruber* | 33.04904 | -117.1255 |
| *C. ruber* | 33.08746 | -117.2893 |
| *C. ruber* | 33.24866 | -116.9625 |
| *C. ruber* | 33.07993 | -117.1278 |
| *C. ruber* | 32.35431 | -117.0545 |
| *C. ruber* | 30.56 | -116.03 |
| *C. ruber* | 29.49686 | -113.4921 |
| *C. ruber* | 29.47643 | -113.4684 |
| *C. ruber* | 32.67519 | -116.1125 |
| *C. ruber* | 32.98995 | -116.4551 |
| *C. ruber* | 23.85677 | -110.2519 |
| *C. ruber* | 22.96737 | -110.0148 |
| *C. ruber* | 29.27167 | -113.39 |
| *C. ruber* | 33.19924 | -116.5987 |
| *C. ruber* | 33.1789 | -117.1581 |
| *C. ruber* | 32.67504 | -116.8368 |
| *C. ruber* | 33.33486 | -116.9554 |
| *C. ruber* | 30.95 | -115.3333 |
| *C. ruber* | 33.07334 | -116.4332 |
| *C. ruber* | 33.37778 | -117.082 |
| *C. ruber* | 32.66019 | -116.0037 |
| *C. ruber* | 33.61253 | -117.7129 |
| *C. ruber* | 30.97167 | -115.74 |
| *C. ruber* | 32.70906 | -117.0126 |
| *C. ruber* | 23.73421 | -109.8079 |
| *C. ruber* | 30.18333 | -115.25 |
| *C. ruber* | 26.01667 | -111.35 |
| *C. ruber* | 33.13809 | -116.3745 |
| *C. ruber* | 32.85466 | -117.2009 |
| *C. ruber* | 33.0819 | -116.7955 |
| *C. ruber* | 32.6678 | -116.8239 |
| *C. ruber* | 32.7091 | -116.7861 |
| *C. ruber* | 33.1331 | -116.32 |
| *C. ruber* | 32.7266 | -116.9354 |
| *C. ruber* | 33.4445 | -117.177 |
| *C. ruber* | 32.66867 | -116.8137 |
| *C. ruber* | 32.82496 | -116.1632 |
| *C. ruber* | 33.22056 | -116.4562 |
| *C. ruber* | 33.70217 | -117.6026 |
| *C. ruber* | 33.4538 | -116.8588 |
| *C. ruber* | 30.31374 | -115.8187 |
| *C. ruber* | 32.97465 | -116.9193 |
| *C. ruber* | 32.80759 | -116.9365 |
| *C. ruber* | 24.98 | -110.62 |
| *C. ruber* | 28.59 | -112.77 |
| *C. ruber* | 27.83 | -113.68 |
| *C. ruber* | 26.87 | -112.54 |
| *C. ruber* | 28.67 | -114.23 |
| *C. ruber* | 23.21 | -109.7 |
| *C. ruber* | 28.84 | -113.78 |
| *C. ruber* | 22.88972 | -109.9156 |
| *C. ruber* | 23.501389 | -110.067 |
| *C. ruber* | 33.090336 | -116.9823 |
| *C. ruber* | 33.91052 | -117.5001 |
| *C. ruber* | 33.57645 | -117.0175 |
| *C. scutulatus* | 31.533333 | -110.1333 |
| *C. scutulatus* | 33.871864 | -111.8583 |
| *C. scutulatus* | 24.35 | -104.39 |
| *C. scutulatus* | 35.510796 | -115.0695 |
| *C. scutulatus* | 34.043316 | -113.0674 |
| *C. scutulatus* | 34.10698 | -112.9912 |
| *C. scutulatus* | 34.18905 | -112.8192 |
| *C. scutulatus* | 35.003265 | -117.7711 |
| *C. scutulatus* | 31.948849 | -108.9093 |
| *C. scutulatus* | 22.75 | -101.08 |
| *C. scutulatus* | 32.826454 | -110.3212 |
| *C. scutulatus* | 32.92377 | -109.9649 |
| *C. scutulatus* | 30.61302 | -104.53 |
| *C. scutulatus* | 34.382874 | -118.3354 |
| *C. scutulatus* | 34.506832 | -117.4827 |
| *C. scutulatus* | 35.219917 | -117.8624 |
| *C. scutulatus* | 35.563473 | -115.7128 |
| *C. scutulatus* | 34.331486 | -116.8301 |
| *C. scutulatus* | 35.101467 | -115.6735 |
| *C. scutulatus* | 34.9081 | -115.6498 |
| *C. scutulatus* | 34.733284 | -118.1133 |
| *C. scutulatus* | 29.53095 | -103.1242 |
| *C. scutulatus* | 29.54775 | -103.5455 |
| *C. scutulatus* | 37.253323 | -115.1036 |
| *C. scutulatus* | 35.21207 | -116.7434 |
| *C. scutulatus* | 34.998936 | -116.9391 |
| *C. scutulatus* | 34.5575 | -114.645 |
| *C. scutulatus* | 34.37693 | -116.8663 |
| *C. scutulatus* | 34.799393 | -117.0219 |
| *C. scutulatus* | 34.20783 | -113.0651 |
| *C. scutulatus* | 34.981407 | -112.3768 |
| *C. scutulatus* | 34.418354 | -112.9171 |
| *C. scutulatus* | 34.426388 | -117.3 |
| *C. scutulatus* | 33.951534 | -112.7296 |
| *C. scutulatus* | 34.5053 | -112.6855 |
| *C. scutulatus* | 31.688208 | -110.7826 |
| *C. scutulatus* | 22.05 | -102.2667 |
| *C. scutulatus* | 22.03333 | -102.2833 |
| *C. scutulatus* | 35.102154 | -118.187 |
| *C. scutulatus* | 22.65 | -102.89 |
| *C. scutulatus* | 34.601616 | -117.4545 |
| *C. scutulatus* | 22.05 | -102.2833 |
| *C. scutulatus* | 22.1 | -102.25 |
| *C. scutulatus* | 35.179855 | -114.8038 |
| *C. scutulatus* | 35.46612 | -114.9043 |
| *C. scutulatus* | 36.007175 | -115.6596 |
| *C. scutulatus* | 25.29 | -101.09 |
| *C. scutulatus* | 20.192 | -99.178 |
| *C. scutulatus* | 20.856 | -99.519 |
| *C. scutulatus* | 31.52 | -109.03 |
| *C. scutulatus* | 29.327696 | -103.5396 |
| *C. scutulatus* | 31.795717 | -109.0596 |
| *C. scutulatus* | 31.870832 | -109.0433 |
| *C. scutulatus* | 31.7025 | -109.1244 |
| *C. scutulatus* | 31.848541 | -109.0493 |
| *C. scutulatus* | 31.9 | -109.1 |
| *C. scutulatus* | 31.791945 | -109.0633 |
| *C. scutulatus* | 33.8303 | -116.5453 |
| *C. scutulatus* | 18.461 | -97.392 |
| *C. scutulatus* | 22.219 | -102.195 |
| *C. scutulatus* | 24.536 | -104.313 |
| *C. scutulatus* | 22.694 | -101.671 |
| *C. scutulatus* | 19.359 | -97.597 |
| *C. scutulatus* | 19.5 | -97.333 |
| *C. scutulatus* | 18.453 | -97.386 |
| *C. scutulatus* | 18.57 | -97.583 |
| *C. scutulatus* | 30.558363 | -104.6495 |
| *C. scutulatus* | 32.00546 | -109.3567 |
| *C. scutulatus* | 31.85 | -112.87 |
| *C. scutulatus* | 31.86 | -113.62 |
| *C. scutulatus* | 18.454 | -97.386 |
| *C. scutulatus* | 19.478 | -97.372 |
| *C. scutulatus* | 19.486 | -97.366 |
| *C. scutulatus* | 35.81859 | -111.2536 |
| *C. scutulatus* | 35.12537 | -118.0744 |
| *C. scutulatus* | 35.05131 | -118.1995 |
| *C. scutulatus* | 34.05628 | -116.3954 |
| *C. scutulatus* | 34.11041 | -116.3704 |
| *C. scutulatus* | 34.134968 | -116.1582 |
| *C. scutulatus* | 34.56023 | -117.1552 |
| *C. scutulatus* | 34.443813 | -116.9818 |
| *C. scutulatus* | 34.5646 | -116.9584 |
| *C. scutulatus* | 34.66712 | -117.1109 |
| *C. scutulatus* | 34.84146 | -117.1592 |
| *C. scutulatus* | 34.76524 | -115.6515 |
| *C. scutulatus* | 32.02927 | -109.0346 |
| *C. scutulatus* | 29.38788 | -103.1475 |
| *C. scutulatus* | 29.19226 | -103.0053 |
| *C. scutulatus* | 29.23561 | -103.0766 |
| *C. scutulatus* | 29.25195 | -103.0999 |
| *C. scutulatus* | 29.30532 | -103.1776 |
| *C. scutulatus* | 25.83 | -104.73 |
| *C. scutulatus* | 31.51 | -106.25 |
| *C. scutulatus* | 30.38 | -106.53 |
| *C. scutulatus* | 31.77 | -113.01 |
| *C. scutulatus* | 31.97 | -113.18 |
| *C. scutulatus* | 31.74 | -113.31 |
| *C. scutulatus* | 30.96 | -112.35 |
| *C. scutulatus* | 29.66829 | -104.5005 |
| *C. scutulatus* | 33.275967 | -112.1841 |
| *C. scutulatus* | 35.137184 | -117.9849 |
| *C. scutulatus* | 31.5373 | -104.8484 |
| *C. scutulatus* | 31.6539 | -104.8643 |
| *C. scutulatus* | 31.7981 | -104.8492 |
| *C. scutulatus* | 23.99 | -104.74 |
| *C. scutulatus* | 26.92 | -105.74 |
| *C. scutulatus* | 34.7876 | -117.4821 |
| *C. scutulatus* | 31.3 | -112.56 |
| *C. scutulatus* | 33.58203 | -111.6524 |
| *C. scutulatus* | 34.515118 | -117.1544 |
| *C. scutulatus* | 35.23012 | -115.5078 |
| *C. scutulatus* | 31.0686 | -105.5699 |
| *C. scutulatus* | 34.55195 | -117.9456 |
| *C. scutulatus* | 32.052982 | -109.0306 |
| *C. scutulatus* | 35.159664 | -115.8014 |
| *C. scutulatus* | 34.69 | -117.7101 |
| *C. scutulatus* | 32.397 | -110.3062 |
| *C. scutulatus* | 34.54778 | -117.5217 |
| *C. scutulatus* | 34.498577 | -117.7607 |
| *C. scutulatus* | 34.445526 | -117.8529 |
| *C. scutulatus* | 34.6954 | -118.1357 |
| *C. scutulatus* | 35.54631 | -118.1611 |
| *C. scutulatus* | 35.471184 | -117.9615 |
| *C. scutulatus* | 34.718803 | -118.0065 |
| *C. scutulatus* | 35.77454 | -117.7433 |
| *C. scutulatus* | 34.43101 | -117.3001 |
| *C. scutulatus* | 35.263096 | -117.6125 |
| *C. scutulatus* | 34.611004 | -116.986 |
| *C. scutulatus* | 34.325863 | -117.4274 |
| *C. scutulatus* | 35.313625 | -115.2579 |
| *C. scutulatus* | 31.18424 | -105.3992 |
| *C. scutulatus* | 37.222847 | -115.0794 |
| *C. scutulatus* | 31.810791 | -109.0476 |
| *C. scutulatus* | 34.85632 | -118.2315 |
| *C. scutulatus* | 34.98055 | -118.1611 |
| *C. scutulatus* | 35.60161 | -117.902 |
| *C. scutulatus* | 34.6954 | -117.7493 |
| *C. scutulatus* | 32.19 | -113.98 |
| *C. scutulatus* | 35.42691 | -117.7751 |
| *C. scutulatus* | 35.34082 | -117.4216 |
| *C. scutulatus* | 35.47214 | -115.8397 |
| *C. scutulatus* | 35.48493 | -117.6245 |
| *C. scutulatus* | 25.23 | -101.09 |
| *C. scutulatus* | 35.12562 | -118.1611 |
| *C. scutulatus* | 35.5161 | -117.5787 |
| *C. scutulatus* | 30.74 | -112.25 |
| *C. scutulatus* | 31.61 | -112.11 |
| *C. scutulatus* | 32.16 | -113.95 |
| *C. scutulatus* | 24.760353 | -99.71027 |
| *C. scutulatus* | 20.72038 | -99.81004 |
| *C. scutulatus* | 35.92465 | -115.6655 |
| *C. scutulatus* | 23.674 | -100.179 |
| *C. scutulatus* | 31.91 | -109.03 |
| *C. scutulatus* | 32 | -109 |
| *C. scutulatus* | 31.94 | -108.89 |
| *C. scutulatus* | 31.94 | -108.95 |
| *C. scutulatus* | 31.99 | -109.04 |
| *C. scutulatus* | 32.23 | -108.95 |
| *C. scutulatus* | 31.95 | -109.04 |
| *C. scutulatus* | 32 | -109.04 |
| *C. scutulatus* | 34.879158 | -118.2963 |
| *C. scutulatus* | 33.1359 | -112.6739 |
| *C. scutulatus* | 33.4643 | -113.1162 |
| *C. scutulatus* | 30.33689 | -104.0994 |
| *C. scutulatus* | 30.308 | -103.8417 |
| *C. scutulatus* | 37.50091 | -115.1944 |
| *C. scutulatus* | 23.99652 | -103.029 |
| *C. scutulatus* | 36.175 | -115.3511 |
| *C. scutulatus* | 35.5509 | -114.3227 |
| *C. scutulatus* | 34.579964 | -118.0384 |
| *C. scutulatus* | 35.49669 | -115.7029 |
| *C. scutulatus* | 34.877537 | -115.408 |
| *C. scutulatus* | 35.31369 | -117.984 |
| *C. scutulatus* | 34.70281 | -116.9775 |
| *C. scutulatus* | 34.223263 | -116.4388 |
| *C. scutulatus* | 32.068 | -112.0389 |
| *C. scutulatus* | 34.614483 | -117.2174 |
| *C. scutulatus* | 35.007282 | -117.1983 |
| *C. scutulatus* | 35.190464 | -117.9845 |
| *C. scutulatus* | 35.013725 | -117.9329 |
| *C. scutulatus* | 35.014206 | -117.9159 |
| *C. scutulatus* | 35.212868 | -117.9844 |
| *C. scutulatus* | 35.1724 | -117.8314 |
| *C. scutulatus* | 35.198547 | -117.7666 |
| *C. scutulatus* | 34.72105 | -115.6776 |
| *C. scutulatus* | 34.996334 | -115.653 |
| *C. scutulatus* | 32.0492 | -110.7114 |
| *C. scutulatus* | 31.8425 | -109.1408 |
| *C. scutulatus* | 32.015266 | -109.0353 |
| *C. scutulatus* | 34.776104 | -118.5682 |
| *C. scutulatus* | 31.5802 | -109.2533 |
| *C. scutulatus* | 31.5421 | -109.2848 |
| *C. scutulatus* | 31.7224 | -109.1099 |
| *C. scutulatus* | 34.8283 | -115.6199 |
| *C. scutulatus* | 34.64884 | -115.6744 |
| *C. scutulatus* | 34.95799 | -115.4563 |
| *C. scutulatus* | 31.5583 | -109.2641 |
| *C. scutulatus* | 31.5546 | -109.2665 |
| *C. scutulatus* | 31.8761 | -109.5823 |
| *C. scutulatus* | 31.6116 | -109.6686 |
| *C. scutulatus* | 31.8707 | -109.048 |
| *C. scutulatus* | 31.7509 | -109.5447 |
| *C. scutulatus* | 31.9428 | -111.9248 |
| *C. scutulatus* | 31.836218 | -109.0307 |
| *C. scutulatus* | 31.931692 | -108.9874 |
| *C. scutulatus* | 31.870531 | -109.0351 |
| *C. scutulatus* | 31.89153 | -109.0353 |
| *C. scutulatus* | 31.6788 | -109.14 |
| *C. scutulatus* | 31.8417 | -109.0484 |
| *C. scutulatus* | 31.9136 | -109.0609 |
| *C. scutulatus* | 31.6045 | -109.2267 |
| *C. scutulatus* | 31.8707 | -109.041 |
| *C. scutulatus* | 31.6343 | -109.1907 |
| *C. scutulatus* | 35.154995 | -117.9841 |
| *C. scutulatus* | 31.8822 | -109.0676 |
| *C. scutulatus* | 31.6087 | -109.2221 |
| *C. scutulatus* | 31.9191 | -109.0118 |
| *C. scutulatus* | 31.9242 | -109.0042 |
| *C. scutulatus* | 31.9325 | -108.9657 |
| *C. scutulatus* | 35.19807 | -117.8679 |
| *C. scutulatus* | 31.36464 | -111.3211 |
| *C. scutulatus* | 32.380806 | -111.1658 |
| *C. scutulatus* | 33.039 | -112.3371 |
| *C. scutulatus* | 33.84514 | -113.6783 |
| *C. scutulatus* | 33.94286 | -112.9528 |
| *C. scutulatus* | 33.86497 | -113.4079 |
| *C. scutulatus* | 33.88172 | -113.3606 |
| *C. scutulatus* | 34.04828 | -113.9086 |
| *C. scutulatus* | 33.423 | -113.4962 |
| *C. scutulatus* | 33.39072 | -113.5257 |
| *C. scutulatus* | 34.04771 | -113.5634 |
| *C. scutulatus* | 34.167057 | -113.2934 |
| *C. scutulatus* | 34.150055 | -113.5043 |
| *C. scutulatus* | 34.513138 | -116.8432 |
| *C. scutulatus* | 34.53075 | -116.8637 |
| *C. scutulatus* | 34.66464 | -116.6991 |
| *C. scutulatus* | 34.64111 | -116.7244 |
| *C. scutulatus* | 35.648067 | -117.7408 |
| *C. scutulatus* | 35.26146 | -115.4874 |
| *C. scutulatus* | 35.17113 | -115.4238 |
| *C. scutulatus* | 35.20699 | -115.4608 |
| *C. scutulatus* | 34.973045 | -118.3056 |
| *C. scutulatus* | 34.50912 | -117.6455 |
| *C. scutulatus* | 33.4483 | -112.0733 |
| *C. scutulatus* | 35.367027 | -117.5983 |
| *C. scutulatus* | 31.4811 | -110.257 |
| *C. scutulatus* | 31.3799 | -110.0416 |
| *C. scutulatus* | 31.7221 | -110.2944 |
| *C. scutulatus* | 35.06301 | -117.5594 |
| *C. scutulatus* | 34.57945 | -117.7102 |
| *C. scutulatus* | 35.30572 | -114.9189 |
| *C. scutulatus* | 36.25327 | -114.3023 |
| *C. scutulatus* | 32.4367 | -111.2247 |
| *C. scutulatus* | 35.46528 | -114.8657 |
| *C. scutulatus* | 36.28028 | -114.1997 |
| *C. scutulatus* | 36.52444 | -114.1294 |
| *C. scutulatus* | 35.1894 | -113.8755 |
| *C. scutulatus* | 32.3281 | -112.86 |
| *C. scutulatus* | 31.8726 | -109.0927 |
| *C. scutulatus* | 31.937386 | -109.0285 |
| *C. scutulatus* | 31.9136 | -109.1366 |
| *C. scutulatus* | 31.8907 | -109.0811 |
| *C. scutulatus* | 29.3226 | -106.45 |
| *C. scutulatus* | 29.82389 | -106.7359 |
| *C. scutulatus* | 29.55139 | -106.3292 |
| *C. scutulatus* | 29.480204 | -106.4017 |
| *C. scutulatus* | 31.6885 | -110.6101 |
| *C. scutulatus* | 34.7747 | -118.4659 |
| *C. scutulatus* | 32.0011 | -109.6128 |
| *C. scutulatus* | 32.0238 | -109.539 |
| *C. scutulatus* | 32.0351 | -109.8314 |
| *C. scutulatus* | 32.2465 | -109.8279 |
| *C. scutulatus* | 31.91303 | -112.9285 |
| *C. scutulatus* | 29.556364 | -106.6542 |
| *C. scutulatus* | 34.913826 | -114.9937 |
| *C. scutulatus* | 34.0526 | -112.8158 |
| *C. scutulatus* | 34.1771 | -113.0286 |
| *C. scutulatus* | 34.0712 | -112.875 |
| *C. scutulatus* | 24.05 | -103.24 |
| *C. scutulatus* | 24.02 | -104.59 |
| *C. scutulatus* | 27.26 | -107.01 |
| *C. scutulatus* | 26.83 | -106.79 |
| *C. scutulatus* | 29.59 | -106.38 |
| *C. scutulatus* | 29.8 | -106.39 |
| *C. scutulatus* | 29.27 | -107 |
| *C. scutulatus* | 31.91 | -112.96 |
| *C. scutulatus* | 19.50194 | -97.3475 |
| *C. scutulatus* | 20.27056 | -97.84722 |
| *C. scutulatus* | 31.55884 | -109.5378 |
| *C. scutulatus* | 32.932407 | -112.804 |
| *C. scutulatus* | 32.267395 | -112.739 |
| *C. scutulatus* | 29.08 | -105.59 |
| *C. scutulatus* | 25.83 | -102.96 |
| *C. scutulatus* | 25.88 | -102.91 |
| *C. scutulatus* | 25.18 | -103.72 |
| *C. scutulatus* | 25.87 | -103.7 |
| *C. scutulatus* | 35.06485 | -114.2249 |
| *C. scutulatus* | 35.19401 | -114.0656 |
| *C. scutulatus* | 35.541164 | -113.2663 |
| *C. scutulatus* | 18.3 | -97.5 |
| *C. scutulatus* | 31.71415 | -109.1135 |
| *C. scutulatus* | 31.58 | -109.25 |
| *C. scutulatus* | 35.268295 | -115.9861 |
| *C. scutulatus* | 35.132263 | -114.1177 |
| *C. scutulatus* | 31.59 | -105.3671 |
| *C. scutulatus* | 34.71644 | -115.2741 |
| *C. scutulatus* | 33.125793 | -115.5016 |
| *C. scutulatus* | 30.16652 | -103.5812 |
| *C. scutulatus* | 32.041668 | -112.8747 |
| *C. scutulatus* | 34.85086 | -114.9497 |
| *C. scutulatus* | 34.54002 | -112.4685 |
| *C. scutulatus* | 35.09374 | -115.4141 |
| *C. scutulatus* | 34.42697 | -117.2997 |
| *C. scutulatus* | 34.58138 | -118.1111 |
| *C. scutulatus* | 34.44378 | -116.9451 |
| *C. scutulatus* | 34.71992 | -118.4253 |
| *C. scutulatus* | 33.5806 | -112.2374 |
| *C. scutulatus* | 34.52908 | -117.3094 |
| *C. scutulatus* | 33.77529 | -111.7165 |
| *C. scutulatus* | 31.52197 | -110.9336 |
| *C. scutulatus* | 34.99475 | -118.0007 |
| *C. scutulatus* | 34.9954 | -117.5847 |
| *C. scutulatus* | 34.30565 | -117.0637 |
| *C. scutulatus* | 34.51829 | -117.995 |
| *C. scutulatus* | 35.18279 | -111.112 |
| *C. scutulatus* | 34.83938 | -114.6091 |
| *C. scutulatus* | 34.61544 | -117.7712 |
| *C. scutulatus* | 34.31728 | -112.9224 |
| *C. scutulatus* | 34.98141 | -112.3768 |
| *C. scutulatus* | 35.10778 | -117.6511 |
| *C. scutulatus* | 35.36889 | -117.3855 |
| *C. scutulatus* | 34.89652 | -117.0326 |
| *C. scutulatus* | 34.60697 | -112.4041 |
| *C. scutulatus* | 34.41836 | -112.9171 |
| *C. scutulatus* | 31.23 | -111.7 |
| *C. scutulatus* | 33.42676 | -111.8252 |
| *C. scutulatus* | 35.05308 | -118.1434 |
| *C. scutulatus* | 34.5794 | -117.4177 |
| *C. scutulatus* | 34.91151 | -117.8388 |
| *C. scutulatus* | 35.31472 | -117.6065 |
| *C. scutulatus* | 35.0303 | -118.0904 |
| *C. scutulatus* | 34.91203 | -117.8153 |
| *C. scutulatus* | 34.41752 | -112.7121 |
| *C. scutulatus* | 35.00193 | -117.6493 |
| *C. scutulatus* | 34.97626 | -117.7268 |
| *C. scutulatus* | 34.1193 | -112.8449 |
| *C. scutulatus* | 33.95153 | -112.7296 |
| *C. scutulatus* | 34.75713 | -112.4677 |
| *C. scutulatus* | 34.64647 | -118.288 |
| *C. scutulatus* | 34.63331 | -118.131 |
| *C. scutulatus* | 34.99062 | -117.5232 |
| *C. scutulatus* | 34.82308 | -112.4435 |
| *C. scutulatus* | 35.03472 | -117.5526 |
| *C. scutulatus* | 34.50748 | -117.1985 |
| *C. scutulatus* | 34.9979 | -118.154 |
| *C. scutulatus* | 35.25853 | -117.7546 |
| *C. scutulatus* | 34.46779 | -117.3987 |
| *C. scutulatus* | 34.91228 | -117.1877 |
| *C. scutulatus* | 31.71667 | -113.2167 |
| *C. scutulatus* | 34.89652 | -116.8565 |
| *C. scutulatus* | 32.64971 | -112.8616 |
| *C. scutulatus* | 34.76293 | -117.4738 |
| *C. scutulatus* | 32.98044 | -111.463 |
| *C. scutulatus* | 35.06432 | -118.1766 |
| *C. scutulatus* | 34.67252 | -118.4316 |
| *C. scutulatus* | 36.00014 | -115.1539 |
| *C. scutulatus* | 34.61234 | -117.3407 |
| *C. scutulatus* | 35.03557 | -117.198 |
| *C. scutulatus* | 31.86139 | -113.4831 |
| *C. scutulatus* | 28.55 | -106.1667 |
| *C. scutulatus* | 23.16 | -103.16 |
| *C. scutulatus* | 22.4 | -101.16 |
| *C. scutulatus* | 33.27596 | -112.1841 |
| *C. scutulatus* | 24.57 | -100.02 |
| *C. scutulatus* | 24.51 | -99.99 |
| *C. scutulatus* | 32.6513 | -112.8613 |
| *C. scutulatus* | 23.58 | -99.75 |
| *C. scutulatus* | 34.77488 | -118.5002 |
| *C. scutulatus* | 34.00169 | -112.9063 |
| *C. scutulatus* | 30.8904 | -102.883 |
| *C. scutulatus* | 33.44834 | -112.3856 |
| *C. scutulatus* | 22.65 | -100.68 |
| *C. scutulatus* | 28.28 | -106.48 |
| *C. scutulatus* | 28.27 | -106.48 |
| *C. scutulatus* | 24.72 | -100.21 |
| *C. scutulatus* | 22.23 | -102.32 |
| *C. scutulatus* | 25.83 | -103.98 |
| *C. scutulatus* | 24.71 | -100.11 |
| *C. scutulatus* | 25.33 | -100.76 |
| *C. scutulatus* | 24.98 | -101.11 |
| *C. scutulatus* | 24.45 | -101.37 |
| *C. scutulatus* | 23.74 | -100.18 |
| *C. scutulatus* | 24.72 | -99.98 |
| *C. scutulatus* | 26.03 | -107.37 |
| *C. scutulatus* | 24.676 | -100.3 |
| *C. scutulatus* | 25.38 | -102.86 |
| *C. scutulatus* | 25.44 | -102.91 |
| *C. scutulatus* | 29.42 | -105.19 |
| *C. scutulatus* | 26.55 | -106.2 |
| *C. scutulatus* | 18.617 | -97.602 |
| *C. scutulatus* | 31.71 | -113.05 |
| *C. scutulatus* | 25.83 | -104.39 |
| *C. scutulatus* | 22.1 | -102.24 |
| *C. scutulatus* | 27.68 | -100.22 |
| *C. scutulatus* | 27.64 | -100.21 |
| *C. scutulatus* | 22.6 | -102.77 |
| *C. scutulatus* | 25.97 | -103.44 |
| *C. scutulatus* | 28.62 | -106.11 |
| *C. scutulatus* | 25.84 | -106.99 |
| *C. scutulatus* | 25.98 | -107.17 |
| *C. scutulatus* | 29.8 | -107.42 |
| *C. scutulatus* | 25.65 | -102.94 |
| *C. scutulatus* | 30.5 | -106.44 |
| *C. scutulatus* | 25.86 | -107.01 |
| *C. scutulatus* | 25.85 | -107.01 |
| *C. scutulatus* | 31.68 | -112.87 |
| *C. scutulatus* | 20.694 | -99.815 |
| *C. scutulatus* | 23.8 | -100.64 |
| *C. scutulatus* | 25.49 | -101 |
| *C. scutulatus* | 23.13 | -101.2 |
| *C. scutulatus* | 23.13 | -101.1156 |
| *C. scutulatus* | 20.69333 | -99.81333 |
| *C. scutulatus* | 35.75 | -114.75 |
| *C. scutulatus* | 35.7166 | -114.833 |
| *C. scutulatus* | 18.436 | -97.367 |
| *C. scutulatus* | 34.67437 | -117.1954 |
| *C. scutulatus* | 35.027836 | -115.5086 |
| *C. scutulatus* | 31.6875 | -109.1347 |
| *C. scutulatus* | 31.42901 | -104.847 |
| *C. scutulatus* | 31.41715 | -105.3494 |
| *C. scutulatus* | 29.99298 | -103.5715 |
| *C. scutulatus* | 32.17733 | -111.1321 |
| *C. scutulatus* | 32.17513 | -108.9467 |
| *C. scutulatus* | 31.14665 | -105.2592 |
| *C. scutulatus* | 31.97019 | -109.0359 |
| *C. scutulatus* | 31.82784 | -105.875 |
| *C. scutulatus* | 31.91856 | -106.0437 |
| *C. scutulatus* | 34.64624 | -118.0079 |
| *C. scutulatus* | 29.69387 | -103.5855 |
| *C. scutulatus* | 30.81084 | -104.1586 |
| *C. scutulatus* | 30.93611 | -104.9061 |
| *C. scutulatus* | 31.26825 | -104.8433 |
| *C. scutulatus* | 34.5825 | -113.503 |
| *C. scutulatus* | 31.13833 | -104.8058 |
| *C. scutulatus* | 33.91495 | -113.5446 |
| *C. scutulatus* | 31.761 | -105.3652 |
| *C. scutulatus* | 32.0033 | -111.2652 |
| *C. scutulatus* | 32.1252 | -111.1953 |
| *C. scutulatus* | 32.19522 | -109.7157 |
| *C. scutulatus* | 32.68442 | -108.9855 |
| *C. scutulatus* | 31.82875 | -105.8739 |
| *C. scutulatus* | 31.20254 | -104.5966 |
| *C. scutulatus* | 31.15382 | -104.5999 |
| *C. scutulatus* | 31.75434 | -105.2007 |
| *C. scutulatus* | 31.18249 | -104.8487 |
| *C. scutulatus* | 31.23742 | -104.8559 |
| *C. scutulatus* | 32.362164 | -105.0899 |
| *C. scutulatus* | 32.24933 | -110.7002 |
| *C. scutulatus* | 31.70938 | -105.3712 |
| *C. scutulatus* | 32.089054 | -105.0931 |
| *C. scutulatus* | 30.92923 | -104.9241 |
| *C. scutulatus* | 31.1544 | -104.8272 |
| *C. scutulatus* | 32.9622 | -112.986 |
| *C. scutulatus* | 32.30631 | -111.2176 |
| *C. scutulatus* | 31.93747 | -111.3901 |
| *C. scutulatus* | 31.42511 | -109.4874 |
| *C. scutulatus* | 31.53559 | -109.2913 |
| *C. scutulatus* | 31.12071 | -105.6312 |
| *C. scutulatus* | 32.1803 | -111.218 |
| *C. scutulatus* | 31.64755 | -109.1662 |
| *C. scutulatus* | 31.81153 | -105.6555 |
| *C. scutulatus* | 33.43481 | -114.7322 |
| *C. scutulatus* | 31.74655 | -105.2652 |
| *C. scutulatus* | 31.65618 | -109.1562 |
| *C. scutulatus* | 32.103893 | -105.663 |
| *C. scutulatus* | 31.59857 | -109.2345 |
| *C. scutulatus* | 31.68768 | -109.1347 |
| *C. scutulatus* | 31.85718 | -109.0315 |
| *C. scutulatus* | 31.62697 | -109.2046 |
| *C. scutulatus* | 31.58943 | -109.2443 |
| *C. scutulatus* | 33.7453 | -112.9206 |
| *C. scutulatus* | 31.77304 | -109.0746 |
| *C. scutulatus* | 31.687 | -111.4872 |
| *C. scutulatus* | 31.93708 | -105.9524 |
| *C. scutulatus* | 32.71639 | -111.9344 |
| *C. scutulatus* | 33.874 | -112.0672 |
| *C. scutulatus* | 31.63801 | -105.3708 |
| *C. scutulatus* | 31.7695 | -105.201 |
| *C. scutulatus* | 32.59047 | -110.6058 |
| *C. scutulatus* | 31.44615 | -105.3489 |
| *C. scutulatus* | 32.37773 | -111.4372 |
| *C. scutulatus* | 32.59322 | -109.6917 |
| *C. scutulatus* | 31.89536 | -106.0478 |
| *C. scutulatus* | 31.9499 | -105.9453 |
| *C. scutulatus* | 31.67561 | -110.5574 |
| *C. scutulatus* | 31.58833 | -110.5612 |
| *C. scutulatus* | 34.04108 | -113.0702 |
| *C. scutulatus* | 33.98617 | -113.1368 |
| *C. scutulatus* | 33.94292 | -113.0266 |
| *C. scutulatus* | 34.04337 | -113.0662 |
| *C. scutulatus* | 33.90997 | -113.5448 |
| *C. scutulatus* | 31.68628 | -110.6189 |
| *C. scutulatus* | 31.30027 | -110.5949 |
| *C. scutulatus* | 32.11437 | -108.9442 |
| *C. scutulatus* | 30.97829 | -104.8803 |
| *C. scutulatus* | 34.0391 | -113.0727 |
| *C. scutulatus* | 32.51408 | -110.1479 |
| *C. scutulatus* | 32.07257 | -109.5134 |
| *C. scutulatus* | 31.99715 | -109.3887 |
| *C. scutulatus* | 32.40282 | -110.1648 |
| *C. scutulatus* | 31.9516 | -109.477 |
| *C. scutulatus* | 30.51122 | -104.4 |
| *C. scutulatus* | 32.084 | -109.9032 |
| *C. scutulatus* | 30.18448 | -104.0776 |
| *C. scutulatus* | 32.03593 | -109.8807 |
| *C. scutulatus* | 33.46352 | -112.9553 |
| *C. scutulatus* | 32.86528 | -112.4149 |
| *C. scutulatus* | 32.51818 | -109.9815 |
| *C. scutulatus* | 32.59448 | -109.8992 |
| *C. scutulatus* | 30.19103 | -104.2741 |
| *C. scutulatus* | 30.79707 | -104.7438 |
| *C. scutulatus* | 30.27345 | -103.8551 |
| *C. scutulatus* | 30.54125 | -104.0899 |
| *C. scutulatus* | 30.93333 | -105.0042 |
| *C. scutulatus* | 34.72358 | -118.3852 |
| *C. scutulatus* | 30.48875 | -103.9585 |
| *C. scutulatus* | 32.49945 | -110.0323 |
| *C. scutulatus* | 33.49402 | -112.6776 |
| *C. scutulatus* | 34.77142 | -118.4222 |
| *C. scutulatus* | 34.70697 | -118.3626 |
| *C. scutulatus* | 30.87935 | -104.7868 |
| *C. scutulatus* | 29.64698 | -103.0809 |
| *C. scutulatus* | 30.96022 | -104.8155 |
| *C. scutulatus* | 30.66273 | -104.5934 |
| *C. scutulatus* | 30.15727 | -104.3685 |
| *C. scutulatus* | 29.7381 | -103.5614 |
| *C. scutulatus* | 35.38563 | -114.0366 |
| *C. scutulatus* | 32.99097 | -109.1782 |
| *C. scutulatus* | 32.70543 | -109.0586 |
| *C. scutulatus* | 32.91657 | -113.5413 |
| *C. scutulatus* | 29.48817 | -103.5313 |
| *C. scutulatus* | 35.72993 | -114.1232 |
| *C. scutulatus* | 31.82582 | -109.0389 |
| *C. scutulatus* | 31.77333 | -109.6908 |
| *C. scutulatus* | 31.81682 | -109.0452 |
| *C. scutulatus* | 31.74727 | -105.1803 |
| *C. scutulatus* | 32.51275 | -111.3212 |
| *C. scutulatus* | 29.33097 | -103.5341 |
| *C. scutulatus* | 32.30507 | -109.8758 |
| *C. scutulatus* | 30.29498 | -103.1368 |
| *C. scutulatus* | 33.06658 | -109.8943 |
| *C. scutulatus* | 34.14757 | -112.8897 |
| *C. scutulatus* | 32.20585 | -112.5666 |
| *C. scutulatus* | 33.96738 | -114.0388 |
| *C. scutulatus* | 33.566666 | -114.2167 |
| *C. scutulatus* | 33.2981 | -114.2214 |
| *C. scutulatus* | 34.69622 | -112.5052 |
| *C. scutulatus* | 29.84147 | -103.2305 |
| *C. scutulatus* | 30.39632 | -104.2567 |
| *C. scutulatus* | 29.7661 | -103.1646 |
| *C. scutulatus* | 30.29545 | -103.1359 |
| *C. scutulatus* | 33.84752 | -112.6132 |
| *C. scutulatus* | 33.03392 | -112.674 |
| *C. scutulatus* | 31.9326 | -105.9527 |
| *C. scutulatus* | 32.07527 | -109.8987 |
| *C. scutulatus* | 32.96573 | -109.9 |
| *C. scutulatus* | 31.97365 | -109.8494 |
| *C. scutulatus* | 34.70393 | -118.2743 |
| *C. scutulatus* | 35.84922 | -114.0871 |
| *C. scutulatus* | 32.89742 | -109.7694 |
| *C. scutulatus* | 29.66662 | -103.1193 |
| *C. scutulatus* | 33.88223 | -114.0327 |
| *C. scutulatus* | 31.55133 | -110.2765 |
| *C. scutulatus* | 32.38042 | -111.3141 |
| *C. scutulatus* | 31.87172 | -106.0489 |
| *C. scutulatus* | 30.26065 | -104.0458 |
| *C. scutulatus* | 31.77246 | -106.0299 |
| *C. scutulatus* | 30.29 | -106.78 |
| *C. scutulatus* | 28.43 | -105.69 |
| *C. scutulatus* | 29.91 | -106.4 |
| *C. scutulatus* | 25.11 | -103.36 |
| *C. scutulatus* | 28.43 | -106.21 |
| *C. scutulatus* | 24.44 | -103.49 |
| *C. scutulatus* | 25.86 | -104.82 |
| *C. scutulatus* | 27.06667 | -105.1833 |
| *C. scutulatus* | 30.20595 | -103.226 |
| *C. scutulatus* | 31.83767 | -105.8435 |
| *C. scutulatus* | 32.3489 | -105.8547 |
| *C. scutulatus* | 31.57572 | -110.2145 |
| *C. scutulatus* | 31.844158 | -105.7764 |
| *C. scutulatus* | 31.70267 | -105.4405 |
| *C. scutulatus* | 31.80283 | -105.6065 |
| *C. scutulatus* | 31.70026 | -105.4609 |
| *C. scutulatus* | 33.233997 | -112.7794 |
| *C. scutulatus* | 26.16 | -105.04 |
| *C. scutulatus* | 31.04143 | -104.8319 |
| *C. scutulatus* | 31.53073 | -105.3535 |
| *C. scutulatus* | 30.27 | -106.49 |
| *C. scutulatus* | 25.44 | -103.68 |
| *C. scutulatus* | 28.3 | -105.82 |
| *C. scutulatus* | 29.85 | -106.39 |
| *C. scutulatus* | 24.76 | -103.57 |
| *C. scutulatus* | 25.3 | -103.58 |
| *C. scutulatus* | 28.46 | -106.23 |
| *C. scutulatus* | 24.39 | -103.47 |
| *C. scutulatus* | 25.93 | -104.75 |
| *C. scutulatus* | 27.13 | -105.25 |
| *C. scutulatus* | 26.18 | -105.07 |
| *C. scutulatus* | 31.6794 | -110.6547 |
| *C. scutulatus* | 31.88389 | -109.095 |
| *C. tigris* | 30.72 | -112.16 |
| *C. tigris* | 29.03 | -110.96 |
| *C. tigris* | 32.97422 | -110.7606 |
| *C. tigris* | 26.77 | -108.7 |
| *C. tigris* | 27.62 | -110.23 |
| *C. tigris* | 33.735428 | -111.5522 |
| *C. tigris* | 29.983334 | -111.1167 |
| *C. tigris* | 28.64 | -111.01 |
| *C. tigris* | 28.24 | -111.02 |
| *C. tigris* | 27.19 | -109.55 |
| *C. tigris* | 30.96 | -112.37 |
| *C. tigris* | 27.07 | -109.32 |
| *C. tigris* | 30.36 | -109.65 |
| *C. tigris* | 27.016666 | -108.9333 |
| *C. tigris* | 32.336388 | -110.9103 |
| *C. tigris* | 28.54 | -111.05 |
| *C. tigris* | 33.458122 | -112.074 |
| *C. tigris* | 31.43 | -112.65 |
| *C. tigris* | 29.63 | -111.03 |
| *C. tigris* | 29.1 | -110.92 |
| *C. tigris* | 28.82 | -110.96 |
| *C. tigris* | 28.49 | -111.04 |
| *C. tigris* | 28.54 | -111.04 |
| *C. tigris* | 28.41 | -111.04 |
| *C. tigris* | 31.6855 | -110.8771 |
| *C. tigris* | 32.385 | -110.7926 |
| *C. tigris* | 32.2399 | -111.1572 |
| *C. tigris* | 32.2481 | -111.1935 |
| *C. tigris* | 31.9535 | -111.9409 |
| *C. tigris* | 32.2748 | -111.1987 |
| *C. tigris* | 31.97133 | -111.0937 |
| *C. tigris* | 30.549887 | -112.4891 |
| *C. tigris* | 29.239357 | -110.9357 |
| *C. tigris* | 29.2281 | -110.9325 |
| *C. tigris* | 32.2217 | -110.9258 |
| *C. tigris* | 27.075836 | -109.3016 |
| *C. tigris* | 32.0173 | -111.6233 |
| *C. tigris* | 31.9481 | -111.9328 |
| *C. tigris* | 28.816458 | -110.9583 |
| *C. tigris* | 32.0163 | -111.6289 |
| *C. tigris* | 32.2151 | -111.1672 |
| *C. tigris* | 30.67 | -112.55 |
| *C. tigris* | 29.28 | -110.96 |
| *C. tigris* | 29.27 | -110.96 |
| *C. tigris* | 27.08 | -109.3 |
| *C. tigris* | 28.75 | -110.94 |
| *C. tigris* | 31.937595 | -111.9205 |
| *C. tigris* | 28.966667 | -110.95 |
| *C. tigris* | 28.636677 | -110.1998 |
| *C. tigris* | 29.26 | -110.66 |
| *C. tigris* | 33.45564 | -110.9273 |
| *C. tigris* | 33.26757 | -112.2804 |
| *C. tigris* | 28.05 | -110.95 |
| *C. tigris* | 29.5 | -110.19 |
| *C. tigris* | 29.76 | -109.81 |
| *C. tigris* | 31.43426 | -111.1815 |
| *C. tigris* | 33.54726 | -112.0207 |
| *C. tigris* | 27.89 | -110.62 |
| *C. tigris* | 27.96 | -110.9 |
| *C. tigris* | 29.36 | -110.96 |
| *C. tigris* | 28.01 | -111.03 |
| *C. tigris* | 27.02 | -109.07 |
| *C. tigris* | 27.97 | -110.86 |
| *C. tigris* | 29.01 | -111.02 |
| *C. tigris* | 30.73 | -109.34 |
| *C. tigris* | 29 | -110.85 |
| *C. tigris* | 30.5 | -109.47 |
| *C. tigris* | 32.24318 | -111.1082 |
| *C. tigris* | 32.21972 | -111.1002 |
| *C. tigris* | 32.19098 | -111.1013 |
| *C. tigris* | 33.5915 | -112.072 |
| *C. tigris* | 32.21635 | -111.0053 |
| *C. viridis* | 37.30981 | -109.2986 |
| *C. viridis* | 37.58423 | -103.3281 |
| *C. viridis* | 37.73057 | -103.423 |
| *C. viridis* | 32.5215 | -108.0048 |
| *C. viridis* | 37.31722 | -104.7514 |
| *C. viridis* | 35.7737 | -102.1523 |
| *C. viridis* | 43.84631 | -102.401 |
| *C. viridis* | 43.58132 | -103.4743 |
| *C. viridis* | 37.16392 | -102.8499 |
| *C. viridis* | 36.37451 | -111.2536 |
| *C. viridis* | 35.94912 | -111.2536 |
| *C. viridis* | 37.02616 | -98.92769 |
| *C. viridis* | 40.378334 | -100.2186 |
| *C. viridis* | 32.129223 | -108.3162 |
| *C. viridis* | 31.783848 | -106.8649 |
| *C. viridis* | 31.827044 | -107.2978 |
| *C. viridis* | 32.842773 | -104.5082 |
| *C. viridis* | 38.44639 | -102.4392 |
| *C. viridis* | 35.353916 | -109.166 |
| *C. viridis* | 37.280277 | -99.32777 |
| *C. viridis* | 33.66096 | -106.4856 |
| *C. viridis* | 33.486595 | -106.5726 |
| *C. viridis* | 33.629093 | -107.0147 |
| *C. viridis* | 33.555187 | -106.3719 |
| *C. viridis* | 34.43086 | -106.0682 |
| *C. viridis* | 34.973167 | -104.9966 |
| *C. viridis* | 32.690678 | -106.7861 |
| *C. viridis* | 35.168137 | -111.4654 |
| *C. viridis* | 44.11 | -103.07 |
| *C. viridis* | 31.79192 | -107.6228 |
| *C. viridis* | 33.654247 | -105.8824 |
| *C. viridis* | 38.261024 | -104.8125 |
| *C. viridis* | 37.39251 | -99.79434 |
| *C. viridis* | 38.01473 | -99.70782 |
| *C. viridis* | 37.43141 | -101.1358 |
| *C. viridis* | 38.72903 | -100.6151 |
| *C. viridis* | 38.85931 | -100.726 |
| *C. viridis* | 38.68181 | -101.0364 |
| *C. viridis* | 39.10286 | -99.32322 |
| *C. viridis* | 37.83045 | -100.6832 |
| *C. viridis* | 37.86745 | -101.0113 |
| *C. viridis* | 37.92259 | -101.2547 |
| *C. viridis* | 39.11853 | -99.15834 |
| *C. viridis* | 38.9802 | -99.0945 |
| *C. viridis* | 39.103 | -99.21901 |
| *C. viridis* | 31.6729 | -110.8853 |
| *C. viridis* | 39.4329 | -99.9719 |
| *C. viridis* | 39.114 | -99.0758 |
| *C. viridis* | 38.74015 | -100.084 |
| *C. viridis* | 37.47082 | -102.3414 |
| *C. viridis* | 38.82634 | -100.3916 |
| *C. viridis* | 32.72829 | -108.4412 |
| *C. viridis* | 38.9559 | -101.9612 |
| *C. viridis* | 37.4455 | -101.3968 |
| *C. viridis* | 31.86583 | -111.4561 |
| *C. viridis* | 39.2182 | -100.1111 |
| *C. viridis* | 38.8119 | -99.6512 |
| *C. viridis* | 37.5601 | -101.3618 |
| *C. viridis* | 38.7231 | -99.3231 |
| *C. viridis* | 39.1517 | -101.613 |
| *C. viridis* | 33.22003 | -111.3867 |
| *C. viridis* | 32.889256 | -110.9258 |
| *C. viridis* | 39.4229 | -99.1927 |
| *C. viridis* | 38.772 | -99.8127 |
| *C. viridis* | 37.1771 | -101.8986 |
| *C. viridis* | 37.38 | -99.6035 |
| *C. viridis* | 38.7182 | -99.5536 |
| *C. viridis* | 38.6975 | -101.9276 |
| *C. viridis* | 39.1817 | -101.7886 |
| *C. viridis* | 37.816 | -100.3455 |
| *C. viridis* | 40.833447 | -107.3814 |
| *C. viridis* | 32.642574 | -108.7375 |
| *C. viridis* | 37.3152 | -98.9175 |
| *C. viridis* | 37.1847 | -101.4749 |
| *C. viridis* | 37.6292 | -101.9253 |
| *C. viridis* | 38.1285 | -101.7606 |
| *C. viridis* | 31.41694 | -108.9292 |
| *C. viridis* | 39.3365 | -99.6122 |
| *C. viridis* | 38.6391 | -99.9218 |
| *C. viridis* | 39.0888 | -99.4658 |
| *C. viridis* | 38.8908 | -99.4291 |
| *C. viridis* | 38.06004 | -100.574 |
| *C. viridis* | 37.9799 | -101.544 |
| *C. viridis* | 43.6584 | -103.4118 |
| *C. viridis* | 37.55691 | -99.07226 |
| *C. viridis* | 43.71165 | -103.3591 |
| *C. viridis* | 38.63987 | -99.80084 |
| *C. viridis* | 38.10054 | -100.0819 |
| *C. viridis* | 37.48962 | -100.2756 |
| *C. viridis* | 37.14069 | -100.4543 |
| *C. viridis* | 39.90791 | -101.4483 |
| *C. viridis* | 39.96836 | -101.5417 |
| *C. viridis* | 39.61644 | -101.3275 |
| *C. viridis* | 39.76283 | -101.1205 |
| *C. viridis* | 38.79988 | -101.224 |
| *C. viridis* | 38.8645 | -101.3708 |
| *C. viridis* | 39.55396 | -101.5577 |
| *C. viridis* | 38.78615 | -100.8763 |
| *C. viridis* | 38.68553 | -100.3315 |
| *C. viridis* | 38.71276 | -100.2368 |
| *C. viridis* | 37.69855 | -102.0163 |
| *C. viridis* | 38.86047 | -101.5958 |
| *C. viridis* | 37.19537 | -99.9722 |
| *C. viridis* | 37.14157 | -99.63399 |
| *C. viridis* | 37.15082 | -98.96064 |
| *C. viridis* | 39.29306 | -103.0667 |
| *C. viridis* | 40.75278 | -104.0028 |
| *C. viridis* | 32.13 | -108.42 |
| *C. viridis* | 34.35 | -107.11 |
| *C. viridis* | 35.33 | -104.06 |
| *C. viridis* | 32.52 | -108.72 |
| *C. viridis* | 33.42 | -104.11 |
| *C. viridis* | 34.78 | -106.94 |
| *C. viridis* | 35.24 | -106.67 |
| *C. viridis* | 36.12 | -106.81 |
| *C. viridis* | 35.08 | -106.19 |
| *C. viridis* | 33.26 | -104.32 |
| *C. viridis* | 31.35 | -108.51 |
| *C. viridis* | 36.34 | -104.55 |
| *C. viridis* | 36.79 | -107.99 |
| *C. viridis* | 34.12 | -104.83 |
| *C. viridis* | 33.48 | -105.65 |
| *C. viridis* | 33.93 | -108.05 |
| *C. viridis* | 33.54 | -105.49 |
| *C. viridis* | 36 | -107.9 |
| *C. viridis* | 35.01 | -107.04 |
| *C. viridis* | 34.37 | -104.99 |
| *C. viridis* | 32.5 | -107.5 |
| *C. viridis* | 36.04 | -107.62 |
| *C. viridis* | 35.04 | -107.42 |
| *C. viridis* | 34.6 | -106.7 |
| *C. viridis* | 31.36 | -108.94 |
| *C. viridis* | 35.19 | -103.76 |
| *C. viridis* | 37 | -107.6 |
| *C. viridis* | 36.94 | -103.67 |
| *C. viridis* | 33.3 | -107.5 |
| *C. viridis* | 35.83 | -106.24 |
| *C. viridis* | 34.42 | -103.49 |
| *C. viridis* | 34.42 | -103.52 |
| *C. viridis* | 32.38 | -103.72 |
| *C. viridis* | 34.32 | -106.96 |
| *C. viridis* | 35.44 | -104.63 |
| *C. viridis* | 36.88 | -104.93 |
| *C. viridis* | 34.3 | -106.9 |
| *C. viridis* | 36.95 | -105.16 |
| *C. viridis* | 35.9 | -107.5 |
| *C. viridis* | 32.23 | -108.08 |
| *C. viridis* | 36.4 | -106.9 |
| *C. viridis* | 31.87 | -108.34 |
| *C. viridis* | 35.76 | -105.93 |
| *C. viridis* | 32.4 | -103.5 |
| *C. viridis* | 35.76 | -106.94 |
| *C. viridis* | 36.39 | -103.32 |
| *C. viridis* | 31.45 | -108.55 |
| *C. viridis* | 34.52 | -104.87 |
| *C. viridis* | 34.68 | -105.67 |
| *C. viridis* | 34.13 | -103.51 |
| *C. viridis* | 35.62 | -106.32 |
| *C. viridis* | 34.35 | -106.24 |
| *C. viridis* | 34.39 | -103.74 |
| *C. viridis* | 32 | -105 |
| *C. viridis* | 31.95 | -108.33 |
| *C. viridis* | 34.52 | -106.61 |
| *C. viridis* | 33.39 | -104.52 |
| *C. viridis* | 36.71 | -104.54 |
| *C. viridis* | 31.94 | -107.68 |
| *C. viridis* | 34.31 | -104.19 |
| *C. viridis* | 35.03 | -106.26 |
| *C. viridis* | 32.9 | -107.6 |
| *C. viridis* | 34 | -104 |
| *C. viridis* | 32.03 | -103.19 |
| *C. viridis* | 32.47 | -103.12 |
| *C. viridis* | 32.06 | -103.64 |
| *C. viridis* | 33 | -106 |
| *C. viridis* | 33.74 | -109.03 |
| *C. viridis* | 35.35 | -106.19 |
| *C. viridis* | 32.54 | -103.06 |
| *C. viridis* | 32.94 | -108.97 |
| *C. viridis* | 33.83 | -103.01 |
| *C. viridis* | 33.08 | -104.13 |
| *C. viridis* | 32.98 | -103.98 |
| *C. viridis* | 33.61 | -107.15 |
| *C. viridis* | 33.77 | -106.98 |
| *C. viridis* | 34.16 | -105.08 |
| *C. viridis* | 32.78 | -106.18 |
| *C. viridis* | 33.91 | -108.49 |
| *C. viridis* | 33.61 | -108.76 |
| *C. viridis* | 34.1 | -107.6 |
| *C. viridis* | 33.46 | -106.96 |
| *C. viridis* | 36 | -103 |
| *C. viridis* | 34.8 | -105.8 |
| *C. viridis* | 32.01 | -108.85 |
| *C. viridis* | 33.1 | -103.8 |
| *C. viridis* | 34.94 | -106.66 |
| *C. viridis* | 33.6 | -103.3 |
| *C. viridis* | 33.45 | -103.79 |
| *C. viridis* | 32.48 | -108.58 |
| *C. viridis* | 32.82 | -103.98 |
| *C. viridis* | 33 | -107 |
| *C. viridis* | 32.65 | -104.22 |
| *C. viridis* | 31.8 | -108.4 |
| *C. viridis* | 31.38 | -108.66 |
| *C. viridis* | 35.7 | -104.2 |
| *C. viridis* | 33.2 | -103.8 |
| *C. viridis* | 35.39 | -103.42 |
| *C. viridis* | 34.63 | -107.38 |
| *C. viridis* | 34.24 | -107.48 |
| *C. viridis* | 31.34 | -108.66 |
| *C. viridis* | 33.15 | -107.38 |
| *C. viridis* | 34.84 | -106.75 |
| *C. viridis* | 33.31 | -103.62 |
| *C. viridis* | 36.95 | -107.53 |
| *C. viridis* | 33.53 | -103.09 |
| *C. viridis* | 31.63 | -108.79 |
| *C. viridis* | 34.67 | -106.53 |
| *C. viridis* | 34.94 | -106.46 |
| *C. viridis* | 35.08 | -107.09 |
| *C. viridis* | 35.6 | -106.8 |
| *C. viridis* | 36.82 | -108.23 |
| *C. viridis* | 34.04 | -108.96 |
| *C. viridis* | 34.52 | -108.14 |
| *C. viridis* | 33.92 | -106.54 |
| *C. viridis* | 34.07 | -107.84 |
| *C. viridis* | 33.05 | -104.69 |
| *C. viridis* | 35.2 | -107.79 |
| *C. viridis* | 35.3 | -106.72 |
| *C. viridis* | 35.2 | -106.64 |
| *C. viridis* | 35.35 | -105.94 |
| *C. viridis* | 33.95 | -107.5 |
| *C. viridis* | 32.4 | -108.73 |
| *C. viridis* | 35.53 | -108.74 |
| *C. viridis* | 35.32 | -108.22 |
| *C. viridis* | 32.55 | -105.63 |
| *C. viridis* | 33.88 | -108.3 |
| *C. viridis* | 34.53 | -108.6 |
| *C. viridis* | 35.23 | -106.81 |
| *C. viridis* | 35.84 | -103.16 |
| *C. viridis* | 32.61 | -107.89 |
| *C. viridis* | 36.41 | -105.05 |
| *C. viridis* | 32 | -105.11 |
| *C. viridis* | 34.13 | -105.2 |
| *C. viridis* | 35.96 | -107.07 |
| *C. viridis* | 34.26 | -104 |
| *C. viridis* | 35.2 | -106.57 |
| *C. viridis* | 34.34 | -105.66 |
| *C. viridis* | 36.17 | -107.88 |
| *C. viridis* | 33.74 | -103.03 |
| *C. viridis* | 32.16 | -103.45 |
| *C. viridis* | 33.68 | -103.34 |
| *C. viridis* | 33.97 | -103.34 |
| *C. viridis* | 35 | -108.08 |
| *C. viridis* | 32.35023 | -108.8803 |
| *C. viridis* | 33.07522 | -106.1547 |
| *C. viridis* | 30.205 | -103.112 |
| *C. viridis* | 36.6517 | -110.4294 |
| *C. viridis* | 37.01944 | -110.7906 |
| *C. viridis* | 37.090942 | -108.731 |
| *C. viridis* | 33.918327 | -106.8287 |
| *C. viridis* | 42.44291 | -107.2176 |
| *C. viridis* | 37.04934 | -110.9835 |
| *C. viridis* | 37.02738 | -111.2408 |
| *C. viridis* | 36.053055 | -108.0283 |
| *C. viridis* | 35.3744 | -109.028 |
| *C. viridis* | 31.9773 | -108.4948 |
| *C. viridis* | 32.8031 | -110.0475 |
| *C. viridis* | 31.65103 | -108.3639 |
| *C. viridis* | 48.56396 | -109.7901 |
| *C. viridis* | 36.543335 | -106.3167 |
| *C. viridis* | 33.6422 | -109.3233 |
| *C. viridis* | 40.469067 | -108.445 |
| *C. viridis* | 40.47299 | -108.311 |
| *C. viridis* | 39.670216 | -103.2255 |
| *C. viridis* | 46.088223 | -113.9815 |
| *C. viridis* | 46.026196 | -106.3863 |
| *C. viridis* | 36.769 | -99.113 |
| *C. viridis* | 37.00908 | -100.3392 |
| *C. viridis* | 43.9833 | -102.7667 |
| *C. viridis* | 32.07 | -106.455 |
| *C. viridis* | 45.292545 | -106.189 |
| *C. viridis* | 47.19849 | -104.6545 |
| *C. viridis* | 37.511448 | -104.7087 |
| *C. viridis* | 33.6181 | -110.3958 |
| *C. viridis* | 34.6921 | -109.7262 |
| *C. viridis* | 33.8475 | -110.9789 |
| *C. viridis* | 36.00578 | -108.7644 |
| *C. viridis* | 32.6485 | -109.8244 |
| *C. viridis* | 32.66 | -110.2708 |
| *C. viridis* | 40.48902 | -107.4011 |
| *C. viridis* | 36.8588 | -111.5048 |
| *C. viridis* | 40.454178 | -108.164 |
| *C. viridis* | 36.7217 | -111.9042 |
| *C. viridis* | 35.3699 | -110.918 |
| *C. viridis* | 34.6084 | -109.2364 |
| *C. viridis* | 34.5439 | -109.5375 |
| *C. viridis* | 30.73417 | -107.0569 |
| *C. viridis* | 30.91139 | -108.36 |
| *C. viridis* | 35.74735 | -99.81611 |
| *C. viridis* | 45.08847 | -108.5745 |
| *C. viridis* | 45.13311 | -108.7632 |
| *C. viridis* | 45.256638 | -108.9332 |
| *C. viridis* | 45.318474 | -108.8016 |
| *C. viridis* | 45.108692 | -108.8375 |
| *C. viridis* | 45.064278 | -109.0275 |
| *C. viridis* | 45.905083 | -108.4439 |
| *C. viridis* | 35.85091 | -99.88985 |
| *C. viridis* | 35.624653 | -105.1855 |
| *C. viridis* | 34.66583 | -98.48612 |
| *C. viridis* | 34.78227 | -98.36963 |
| *C. viridis* | 36.762123 | -101.6637 |
| *C. viridis* | 36.511425 | -103.049 |
| *C. viridis* | 36.90372 | -102.9754 |
| *C. viridis* | 36.605606 | -102.7078 |
| *C. viridis* | 34.980854 | -99.86878 |
| *C. viridis* | 38.043724 | -102.6203 |
| *C. viridis* | 36.57773 | -101.3869 |
| *C. viridis* | 36.62795 | -102.5132 |
| *C. viridis* | 36.605534 | -99.31818 |
| *C. viridis* | 37.37 | -105.75 |
| *C. viridis* | 47.73563 | -110.4771 |
| *C. viridis* | 32.707577 | -110.1022 |
| *C. viridis* | 37.105026 | -102.5796 |
| *C. viridis* | 37.4333 | -100.9833 |
| *C. viridis* | 47.60107 | -110.2663 |
| *C. viridis* | 37.0312 | -101.7422 |
| *C. viridis* | 47.460594 | -110.1132 |
| *C. viridis* | 49.694736 | -114.0344 |
| *C. viridis* | 49.453957 | -110.8099 |
| *C. viridis* | 35.27842 | -112.191 |
| *C. viridis* | 35.02422 | -110.6974 |
| *C. viridis* | 35.86144 | -112.2485 |
| *C. viridis* | 44.42 | -103.71 |
| *C. viridis* | 35.28304 | -112.6387 |
| *C. viridis* | 35.06373 | -109.7705 |
| *C. viridis* | 37.11 | -110.06 |
| *C. viridis* | 36.08331 | -112.1341 |
| *C. viridis* | 35.8764 | -110.6404 |
| *C. viridis* | 35.19811 | -111.6513 |
| *C. viridis* | 49 | -110.5931 |
| *C. viridis* | 49.190277 | -110.4722 |
| *C. viridis* | 49.7 | -112.8167 |
| *C. viridis* | 50.95 | -110 |
| *C. viridis* | 49.906944 | -111.0508 |
| *C. viridis* | 34 | -112 |
| *C. viridis* | 53.523613 | -113.5264 |
| *C. viridis* | 50.16111 | -110.5 |
| *C. viridis* | 50.643055 | -110.0272 |
| *C. viridis* | 50.34722 | -111.8217 |
| *C. viridis* | 49 | -110.6639 |
| *C. viridis* | 49 | -110.5486 |
| *C. viridis* | 51.408333 | -112.6445 |
| *C. viridis* | 49.933334 | -112.6833 |
| *C. viridis* | 50.083332 | -110.7833 |
| *C. viridis* | 49.931946 | -111.4911 |
| *C. viridis* | 49.738888 | -111.9889 |
| *C. viridis* | 42.66016 | -100.8428 |
| *C. viridis* | 38.89722 | -100.1131 |
| *C. viridis* | 34.267 | -106.083 |
| *C. viridis* | 34.71667 | -98.70721 |
| *C. viridis* | 46.47385 | -100.5948 |
| *C. viridis* | 42.709167 | -102.0097 |
| *C. viridis* | 42.548054 | -100.8161 |
| *C. viridis* | 40.643055 | -99.00584 |
| *C. viridis* | 36.75571 | -98.35437 |
| *C. viridis* | 41.141666 | -102.9814 |
| *C. viridis* | 32.526234 | -103.8472 |
| *C. viridis* | 32.63983 | -108.7403 |
| *C. viridis* | 32.232483 | -107.2635 |
| *C. viridis* | 31.82693 | -106.4212 |
| *C. viridis* | 31.82709 | -105.887 |
| *C. viridis* | 31.67511 | -105.3709 |
| *C. viridis* | 31.810404 | -107.8155 |
| *C. viridis* | 32.242214 | -107.4597 |
| *C. viridis* | 31.80817 | -105.0766 |
| *C. viridis* | 35.52411 | -110.446 |
| *C. viridis* | 33.042404 | -104.4378 |
| *C. viridis* | 31.11338 | -104.6434 |
| *C. viridis* | 32.0467 | -105.1022 |
| *C. viridis* | 31.89923 | -106.0483 |
| *C. viridis* | 30.53385 | -104.3543 |
| *C. viridis* | 36.37705 | -104.7367 |
| *C. viridis* | 35.680206 | -108.7186 |
| *C. viridis* | 31.35011 | -105.9286 |
| *C. viridis* | 36.749027 | -105.6725 |
| *C. viridis* | 37.27015 | -101.5948 |
| *C. viridis* | 36.26611 | -101.8517 |
| *C. viridis* | 32.27945 | -106.9868 |
| *C. viridis* | 31.807825 | -107.2652 |
| *C. viridis* | 31.8424 | -106.5699 |
| *C. viridis* | 31.51935 | -106.131 |
| *C. viridis* | 31.821318 | -106.732 |
| *C. viridis* | 32.4109 | -107.068 |
| *C. viridis* | 31.837 | -108.1022 |
| *C. viridis* | 31.78475 | -106.5851 |
| *C. viridis* | 31.80487 | -107.0262 |
| *C. viridis* | 32.72505 | -107.5726 |
| *C. viridis* | 32.07622 | -105.7459 |
| *C. viridis* | 31.56558 | -108.8747 |
| *C. viridis* | 31.76343 | -104.9389 |
| *C. viridis* | 31.54415 | -103.8222 |
| *C. viridis* | 30.66695 | -104.5989 |
| *C. viridis* | 33.25453 | -107.3281 |
| *C. viridis* | 34.865 | -102.0496 |
| *C. viridis* | 34.80743 | -101.8126 |
| *C. viridis* | 31.59357 | -106.0214 |
| *C. viridis* | 31.52392 | -106.1231 |
| *C. viridis* | 31.76993 | -106.593 |
| *C. viridis* | 32.339436 | -106.4205 |
| *C. viridis* | 36.58294 | -98.9678 |
| *C. viridis* | 31.48475 | -106.5851 |
| *C. viridis* | 31.47107 | -105.4795 |
| *C. viridis* | 34.565266 | -105.2791 |
| *C. viridis* | 33.16255 | -108.8133 |
| *C. viridis* | 31.78475 | -106.5851 |
| *C. viridis* | 31.783043 | -106.9779 |
| *C. viridis* | 31.69457 | -105.9978 |
| *C. viridis* | 31.76993 | -106.4988 |
| *C. viridis* | 31.76993 | -106.7988 |
| *C. viridis* | 31.90794 | -106.6564 |
| *C. viridis* | 32.34879 | -106.1015 |
| *C. viridis* | 31.89927 | -106.4208 |
| *C. viridis* | 31.81273 | -105.6609 |
| *C. viridis* | 31.93 | -109.01 |
| *C. viridis* | 32.64 | -108.74 |
| *C. viridis* | 32.09 | -104.07 |
| *C. viridis* | 32.028275 | -104.0406 |
| *C. viridis* | 32.66 | -108.11 |
| *C. viridis* | 32.32 | -107.79 |
| *C. viridis* | 31.7511 | -103.0833 |
| *C. viridis* | 36.255 | -106.9424 |
| *C. willardi* | 23.398 | -104.22 |
| *C. willardi* | 23.387 | -104.251 |
| *C. willardi* | 23.437 | -104.279 |
| *C. willardi* | 23.88 | -105.15 |
| *C. willardi* | 23.81 | -105.16 |
| *C. willardi* | 23.87 | -105.21 |
| *C. willardi* | 23.85 | -105.31 |
| *C. willardi* | 24.09556 | -105.5492 |
| *C. willardi* | 29.392097 | -106.8987 |
| *C. willardi* | 29.05 | -107.9 |
| *C. willardi* | 28.10361 | -108.1108 |
| *C. willardi* | 28.1 | -108.38 |
| *C. willardi* | 30.014645 | -108.4168 |
| *C. willardi* | 31.528719 | -108.7474 |
| *C. willardi* | 31.59 | -108.77 |
| *C. willardi* | 31.58 | -108.78 |
| *C. willardi* | 31.59 | -108.78 |
| *C. willardi* | 31.57 | -108.78 |
| *C. willardi* | 31.5 | -109.05 |
| *C. willardi* | 31.5 | -109.1 |
| *C. willardi* | 28.15 | -109.34 |
| *C. willardi* | 31.9297 | -109.3817 |
| *C. willardi* | 30.37 | -109.69 |
| *C. willardi* | 30.97471 | -110.0194 |
| *C. willardi* | 30.89 | -110.03 |
| *C. willardi* | 29.81 | -110.33 |
| *C. willardi* | 31.44584 | -110.3856 |
| *C. willardi* | 31.4889 | -110.4075 |
| *C. willardi* | 31.41955 | -110.4255 |
| *C. willardi* | 31.4167 | -110.4306 |
| *C. willardi* | 31.5222 | -110.5014 |
| *C. willardi* | 30.04 | -110.8 |
| *C. willardi* | 31.616268 | -110.8083 |
