# Supplementary figures and images for: Does size matter? An analysis of the niche width and vulnerability to climate change of fourteen species of the genus Crotalus from North America
Source: PeerJ. 2022 Apr 5;10:e13154. doi: 10.7717/peerj.13154 (PMC8992643; doi:10.7717/peerj.13154)

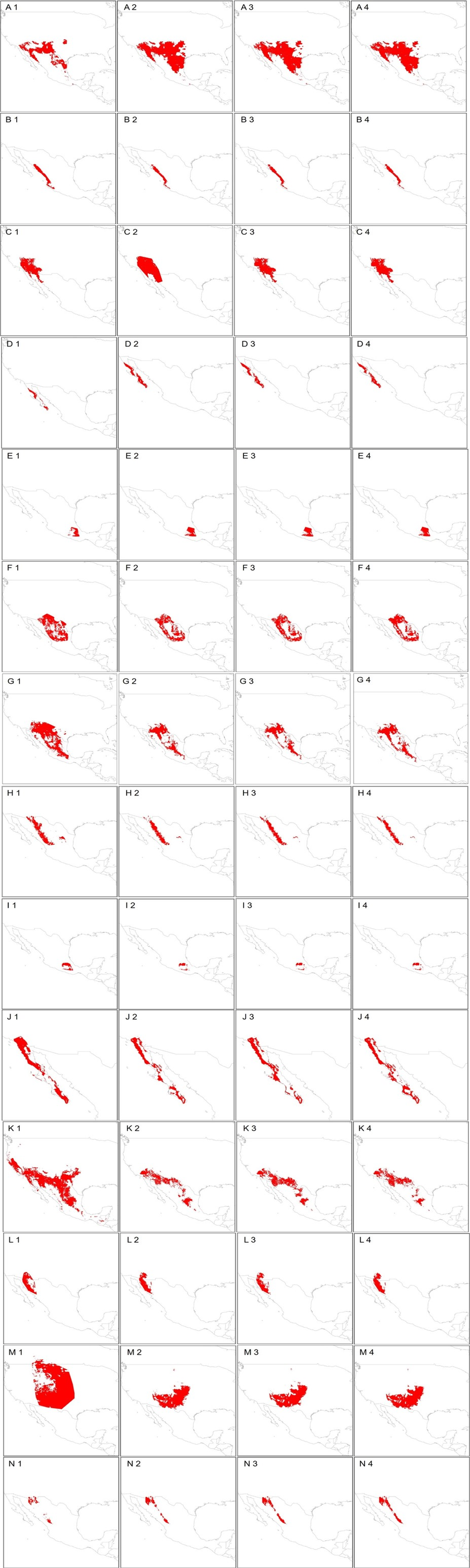

Supplement: Supplemental Information 2 — The models correspond to the current climatic suitability (1) and for climate change models BCC-CSM2-MR (2), CNRM-CM6-1(3), and IPSL-CM6A-LR (4) for the period 2021-2040. [file peerj-10-13154-s002.png]
